# Asb10 accelerates pathological cardiac remodeling by stabilizing HSP70

**DOI:** 10.1038/s41419-025-07735-5

**Published:** 2025-05-22

**Authors:** Ke Lin, Wenjie Wei, Songzan Chen, Yingchao Gong, Xingchen Wang, Meihui Wang, Ran Li, Yanbo Zhao, Shengjie Xu, Chongying Jin, Chenyang Jiang, Guosheng Fu, Qinfeng Li

**Affiliations:** 1 Department of Cardiology, Sir Run Run Shaw Hospital, Zhejiang University School of Medicine, Hangzhou, China; 2 Key Laboratory of Cardiovascular Intervention and Precision Medicine of Zhejiang Province, Hangzhou, China; 3 Engineering Research Center for Cardiovascular Innovative Devices of Zhejiang Province, Hangzhou, China; 4 Department of Nephrology, Sir Run Run Shaw Hospital, Zhejiang University School of Medicine, Hangzhou, China

**Keywords:** Heart failure, Hypertension

## Abstract

Cardiac hypertrophy is a pivotal risk factor for heart failure. Hypertension-induced pressure overload triggers left ventricular hypertrophy and leads to heart failure. Although the precise mechanisms remain incompletely elucidated, recent studies highlighted the role of ubiquitin-proteasome system in this process. As a heart tissue-enriched E3 ligase, the function of Asb10 in cardiac hypertrophy remains unknown. Here, we aimed to dissect the role of Asb10 in the pathogenesis of cardiac hypertrophy and heart failure. Through integrated bioinformatic screening of GEO datasets and experimental verifications, we identified Asb10 as the downregulated gene in cardiac hypertrophy. Adenoviral overexpression of Asb10 exacerbated hypertrophic growth in NRVMs treated with phenylephrine or endothelin-1. Mechanistically, immunoprecipitation–mass spectrometry and co-immunoprecipitation assays revealed that Asb10 binds HSP70 and competitively blocks STUB1-mediated ubiquitination and degradation of HSP70, thereby stabilizing HSP70. Pharmacological or small interfering RNA-induced inhibition of HSP70 partially reversed Asb10 overexpression-induced hypertrophic growth in NRVMs. In vivo, mice administrated with AAV9-*Asb10* exhibited worse cardiac function and more severe interstitial fibrosis following TAC surgery, while mice injected with AAV9-*shAsb10* showed improved outcomes. Furthermore, we observed that the effects of Asb10 on cardiac hypertrophy were attributed to the elevation of HSP70, cardiac inflammation, and activation of pHDAC2^S394^. Collectively, these findings demonstrate that Asb10 stabilizes HSP70 via competitively inhibiting STUB1-mediated ubiquitin-dependent degradation, thereby exacerbating cardiac hypertrophy, highlighting the role of Asb10 in hemodynamic stress-induced cardiac hypertrophy and heart failure.

## Introduction

The primary function of the heart is to maintain perfusion of peripheral organs, matching their metabolic demands [1]. However, under stress conditions such as chronic hypertension and aortic stenosis, the heart is subjected to hemodynamic pressure overload. In response, the heart initially develops compensatory concentric hypertrophy to overcome the stress and maintain its pumping function. However, as the stress persists, the heart may progress to irreversible heart failure [2]. Currently, a wide range of drugs are clinically available, including β-adrenergic receptor blockers and renin-angiotensin-aldosterone system inhibitors, but their curative effects seem to be limited [3]. Hence, a better and more comprehensive understanding of the molecular mechanisms governing pathological cardiac hypertrophy is needed to identify new therapeutic targets.

The ubiquitin-proteasome system (UPS) is one of the major pathways for intracellular protein quality control and plays critical roles in both physiological and pathological processes, including cardiovascular pathologies [4, 5]. Cumulative evidence has established that targeting UPS, including the E3 ubiquitin ligases and de-ubiquitinating enzymes, could be a promising therapeutic strategy for pathological cardiac hypertrophy and heart failure [6, 7]. Ankyrin and SOCS Box-containing protein-10 (Asb10) is one of the 18 proteins that comprise the ASB family, and it contains seven ankyrin repeats and one SOCS box [8]. As part of the UPS, ASB proteins, including Asb1, Asb2 and Asb3, have been reported to play critical roles in ischemic heart disease (ICM), cardiac developmental biology and spontaneous hypertension [9–12]. However, few studies focused on Asb10’s role in cardiovascular research. Previously, Asb10 was identified as an open-angle glaucoma (POAG)-related protein that mainly regulates ubiquitin-mediated degradation pathways in human trabecular meshwork cells of POAG [13–15]. Asb10 was also reported to function as an effective E3 ligase, mediating the ubiquitination and subsequent degradation of TEM8 in breast cancer [16]. Interestingly, a high level of *Asb10* mRNA expression was observed in muscle tissues, including cardiac muscle and skeletal muscle [17]. As a cardiac tissue-enriched E3 ligase, whether Asb10 is associated with pathological cardiac hypertrophy and heart failure remains unknown.

The 70-kDa heat shock protein (HSP70) is a ubiquitous molecular chaperone that assists in protein folding and mediates the degradation of misfolded proteins [18]. As a sentinel chaperone, HSP70 undergoes upregulation in response to thermal stress, ischemia, and other cellular stresses to promote cell survival, and returns to normal levels through sequential ubiquitination mediated by STUB1 [19]. While extensive studies have revealed HSP70’s protective role in myocardial ischemia and ischemia-reperfusion injury [20], its function in pathological cardiac hypertrophy and remodeling appears to be entirely different. Long-term overexpression of HSP70 does not protect the heart against adverse cardiac remodeling [21], whereas inhibition of HSP70 suppresses the development of cardiac hypertrophy [22, 23]. Given that Asb10 was reported to interact with HSP70 in the development of POAG [15], the role of this interaction in cardiac hypertrophy and heart failure remains to be investigated.

In this study, we aimed to elucidate the role of Asb10 in pathological cardiac hypertrophy and heart failure by using gain- and loss-of-function approaches and further explore the underlying molecular mechanisms through high-throughput protein interaction analyses.

## Materials and methods

### Data collection and processing

All datasets used in this study are available in GEO according to the accession number. Cellular data were employed for the identification of differentially expressed genes (DEGs), with mouse and human data subsequently utilized for validation purposes.

For cellular hypertrophy data, four RNA-sequence datasets were selected, including GSE153763 (primary neonatal rat ventricular myocytes (NRVMs) treated with PBS or PE for 24 h in triplicate) [24], GSE155913 (NRVMs treated with PBS or PE for 24 h in duplicate) [25], GSE173737 (NRVMs treated with PBS or PE for 36 h in triplicate) [26], and GSE175884 (NRVMs treated with PBS or PE for 36 h in triplicate) [27].

For pathological cardiac hypertrophy data based on the transverse aortic constriction (TAC) model, two array datasets and one RNA-sequence dataset were selected, including GSE79536 (array data from left ventricular tissues of wild-type mice adopted with sham or TAC surgery for 3 weeks) [28], GSE136308 (array data from left ventricular tissues of wild-type mice adopted with sham or TAC surgery for 6 weeks) [29] and GSE180720 (RNA sequence data from isolated adult cardiomyocytes of wild-type mice adopted with sham or TAC surgery for 1 or 8 weeks) [30].

For data from patients with hypertrophic cardiomyopathy (HCM) or heart failure (HF), three RNA-sequence datasets were enrolled, including GSE180313 (containing left ventricular tissues from 7 donors and 13 HCM patients) [31], GSE135055 (containing left ventricular tissues from 13 male HF patients and 5 male healthy donors, 8 female HF patients were excluded to avoid gender-dependent differences) [32], and GSE161472 (containing left ventricular tissues from 4 female non-failure and 5 female HF patients, male data were excluded since poor separation between NF and HF group) [33].

R version 3.6.1 was used for DEG screening. All four datasets were the expression matrix of raw gene counts. We used the limma package to normalize the dataset and screen for DEGs [34]. Then, we merged these four datasets and used the sva package to correct the batch effect [35]. The principal component analysis (PCA) plot was utilized to show the group distribution before and after the correction. DEGs in merged datasets were also screened using the limma package and defined as fold change ≥ 2 and *adjust P. Val* < 0.05. Low-expression genes were defined as average gene expression < 0 after normalization.

For validation datasets, GEO2R (www.ncbi.nlm.nih.gov/geo/geo2r) was used following the instructions.

### General reagents and chemicals

Phenylephrine (PE, #P6126), endothelin-1 (ET-1, #05-23-3800), puromycin (#540222) and antibodies against FLAG (#SAB2702252), and α-actinin (#SAB4200813) were purchased from Sigma-Aldrich (St. Louis, Mo, USA). Cycloheximide (HY-12320), MG132 (HY-13259), chloroquine (HY-17589A), and pifithrin-μ (#HY-10940) were obtained from MedChemExpress (New Jersey, USA). Antibodies against MYH7 (#22280-1-AP), MYH6 (#22281-1-AP), ANP (#27426-1-AP), BNP (#13299-1-AP), COL1a1 (#67288-1-Ig), CTGF (#25474-1-AP), αSMA (#14395-1-AP), HRP linked GAPDH (#HRP-60004), HSP70 (#10995-1-AP), HSC70 (#10654-1-AP), HIF1AN (#10646-1-AP), and FLAG (#20543-1-AP) were purchased from ProteinTech (Wuhan, China). Antibody against Asb10 (#sc-100677) was purchased from Santa Cruz Biotechnology (CA, USA). Antibodies against phospho-HDAC2^S394^ (#ab75602), HDAC2 (#ab16032), HSP60 (#ab46798), HSP27 (#ab2790), and STUB1 (#ab134064) were purchased from Abcam (Cambridge, UK). Antibodies against LC3 (#3868), Myc (#2276), EGFP (#2555), Ub (#3936), and HA (#3724) were purchased from Cell Signaling Technology (Boston, USA). Horseradish peroxidase-conjugated secondary antibodies (#33101ES, #33201ES) were purchased from Yeason Biotechnology (Shanghai, China). Plasmids of Ub-HA, STUB1-EGFP, HSPA1-Myc, ASB10-FLAG (human), and their truncations were constructed by General Biotechnology (Chuzhou, China). Adenovirus-packed vector and Asb10 (Rattus, Ad-*Asb10*) were constructed by Genechem Biotechnology (Shanghai, China). Adeno-associated virus 9 (AAV9) packed vector and Asb10 with cTNT promoter (Mus, AAV9-cTNT-*Asb10*) and shAsb10 with cTNT promoter (Mus, AAV9-cTNT-*shAsb10*) were constructed by Obio Technology (Shanghai, China). Series of siRNA oligos targeting HSP70 and STUB1 were obtained from Genepharma Biotechnology (Shanghai, China).

### Cell culture and treatments

NRVMs were isolated from the left ventricles of 1 to 2-day-old Sprague-Dawley rats as previously described [36]. A 2-h pre-plating was used to remove fibroblasts, and NRVMs were then plated with the medium (DMEM/M199 = 3:1 with 10% FBS, 1% penicillin/streptomycin and 100 μM Brdu). After 24 h, the medium was changed to serum-free medium (DMEM/M199 = 3:1). NRVMs were then subjected to further treatments, including siRNA transfection, adenovirus infection, hypertrophy stimulation, and puromycin incubation.

HEK293T cells were obtained from ATCC (Manassas, VA, USA) and cultured in DMEM supplemented with 10% FBS, and 1% penicillin/streptomycin. Cells were transfected with plasmids and subjected to further treatments, including CHX, MG132, and CQ incubation. Transfection was performed using Lipofectamin^TM^ RNAiMAX (Thermo, CA, USA).

### Quantitative real-time PCR (qRT-PCR)

Total RNA was extracted from cells or cardiac tissues using an RNA isolation kit (cWbio, China). RNA was then quantified in NanoDrop2000 (Thermo, USA) and reverse transcribed into cDNA using Evo M-MLV RT Master Mix (Agbio, China) according to the instruction. Finally, 10 ng cDNA, 5 μL qPCR SYBR Mix (Yeason, China), 3.6 μL ddH_2_O, and 0.2 μL forward and reverse primers (the detailed sequences are shown in Table S1) were mixed in the plate. The reaction was then detected in QuantStudio^TM^ 6 Flex Real-Time PCR System (Thermo, USA).

### Immunoprecipitation-mass spectrometry assay

NRVMs were infected with adenovirus-packed vector or Asb10 for 30 h, and the lysates were then collected and incubated with anti-FLAG antibody and Protein A&G magnet beads overnight at 4 °C. Mass spectrometry analysis was performed by APT Biotechnology (Shanghai, China). The MS data was analyzed in MQ software, and the Asb10 interactome was ranked according to the Asb10/vector ratio. Raw data was deposited in the Integrated Proteome Resources (iProX) with the ID of PXD061568

(http://proteomecentral.proteomexchange.org/cgi/GetDataset?ID=PXD061568).

### Co-immunoprecipitation assay and immunoblotting

For co-immunoprecipitation assay, lysates were collected and then incubated with various antibodies (FLAG, Myc or HSP70) and Protein A&G magnet beads overnight at 4 °C. The beads were washed with 0.5% PBST for 5 times, resuspended in a loading buffer, and boiled at 100°C for 10 min. Samples were then used for western blot.

For immunoblotting, cell or tissue lysates were first collected and quantified using a BCA kit (Beyotime, Shanghai, China). Samples were then separated by SDS-PAGE gel and electrophoretically transferred to the PVDF membrane. After blocking with 5% non-fat milk for 2 h, the membranes were incubated with the primary antibody overnight at 4 °C. The next day, after washing with 0.1% TBST for 3 times, the membranes were incubated with HRP-conjugated secondary antibody for 1 hour at room temperature. Finally, the immunoreactive bands were visualized and captured on an Amersham Imager 600 system (GE Healthcare, England) using an Enhanced ECL kit (Abclonal, Wuhan, China).

### Randomization and blinding procedures

We used a random number table to perform randomization. Briefly, all animal experiments were conducted and analyzed in a blinded manner. Each mouse was initially assigned a temporary random number within a specified weight range. After random group allocation, mice were given permanent numerical designations in their cages. To minimize bias, cages were randomly selected from the pool of all cages. Data collection and analysis were carried out by two independent observers who were unaware of the group assignments or treatments.

### Inclusion and exclusion criteria

The criteria were established prior to the study. Briefly, mice in the TAC group were included when post-surgical vascular ultrasound confirmed consistent and significant aortic restriction. Mice were excluded if the aortic restriction was excessively severe or insufficient compared to other subjects, or if they died prematurely, preventing the collection of echocardiographic and histological data.

### Animals and treatments

All animal experimental procedures were approved by the Animal Care Committee of the Zhejiang University-affiliated Sir Run Run Shaw Hospital (SRRSH202402283) and adhered to the NIH guideline for the care and use of laboratory animals [37]. Male C57BL/6 mice of 8 weeks old were obtained from GemPharmatech (Nanjing, China) and maintained on a 12:12 h light–dark cycle in temperature-controlled mouse facilities. All animals had free access to the standard rodent diet (Xietong Biotechnology, Nanjing, China) and sterile water. The animals were acclimated to the SPF laboratory for 1 week before initiating the studies. Mice were kept at 5 per cage and were randomized into different groups. At the end of the study, mice were anesthetized with 1% sodium phenobarbital and then sacrificed. Heart tissues and serum were collected for further analysis.

To induce pathological cardiac hypertrophy, we performed transverse aortic constriction (TAC) surgery in mice following established methods [36]. Briefly, male mice matched for age and body weight were anesthetized with intraperitoneal 1% sodium phenobarbital. After confirming anesthesia by the absence of a toe-pinch reflex, the left chest was opened to expose the thoracic aorta. A 27-gauge needle threaded with 6-0 silk suture was used to ligate the thoracic aorta, and was quickly removed after ligation. Finally, the chest was closed. The animals were monitored during recovery from anesthesia and then returned to the animal facility for housing. Sham-operated mice underwent the same procedures without ligation.

In this study, four cohorts of mice were used as follows.

#### Cohort 1

Twenty-two to 25 g WT mice were randomly divided into 5 groups and received either sham or TAC surgery over different time intervals.

#### Cohort 2

Twenty to 22 g WT mice were randomly divided into 6 groups. 1) WT mice injected with saline (Saline), 2) WT mice injected with 5.0E + 11 v.g. AAV9-empty vector (AAV9-*EV*), 3) WT mice injected with 1.0E + 11 v.g. AAV9-*Asb10* (AAV9-1), 4) WT mice injected with 2.0E + 11 v.g. AAV9-*Asb10* (AAV9-2), 5) WT mice injected with 3.0E + 11 v.g. AAV9-*Asb10* (AAV9-3) and 6) WT mice injected with 5.0E + 11 v.g. AAV9-*Asb10* (AAV9-5). AAV9 infection was achieved via tail vein injection. Four weeks after injection, mice were sacrificed, and the optimal virus concentration was determined by assessing the protein and mRNA expression levels of Asb10.

#### Cohort 3

Twenty to 22 g WT mice were randomly divided into 4 groups. (1) WT mice injected with 5.0E + 11 v.g. AAV9-empty vector and received sham surgery (AAV9-*EV*+Sham), (2) WT mice injected with 5.0E + 11 v.g. AAV9-*Asb10* and received sham surgery (AAV9-*Asb10*+Sham), (3) WT mice injected with 5.0E + 11 v.g. AAV9-empty vector and received TAC surgery (AAV9-*EV* + TAC) and (4) WT mice injected with 5.0E + 11 v.g. AAV9-*Asb10* and received TAC surgery (AAV9-*Asb10* + TAC). AAV9 infection was achieved via tail vein injection. Four weeks after the infection, mice received either sham or TAC surgery.

#### Cohort 4

Twenty to 22 g-weight WT mice were randomly divided into four groups. (1) WT mice injected with 5.0E + 11 v.g. AAV9-empty vector and received sham surgery (AAV9-*EV*+Sham), (2) WT mice injected with 5.0E + 11 v.g. AAV9-*shAsb10* and received sham surgery (AAV9-*shAsb10*+Sham), (3) WT mice injected with 5.0E + 11 v.g. AAV9-empty vector and received TAC surgery (AAV9-*EV* + TAC) and (4) WT mice injected with 5.0E + 11 v.g. AAV9-*shAsb10* and received TAC surgery (AAV9-*shAsb10* + TAC). AAV9 infection was achieved via tail vein injection. Four weeks after the infection, three mice from each of groups (1) and (2) were sacrificed to assess knockdown efficiency, while the remaining mice underwent either sham or TAC surgery.

### Assessment of cardiac function

Cardiac function was assessed by non-invasive transthoracic echocardiography using a Vevo-3100 high-resolution imaging system (Fujifilm Visual Sonics, Japan). All mice were assessed at baseline and at the indicated time points after surgery. Briefly, the short-axis view of M-mode images at the level of papillary muscles was captured and analyzed to determine the parameters of cardiac function. The structural parameters consisted of left ventricular internal diameters and anterior and posterior wall diameters at systolic and diastolic phase (LVIDs/d, LVAWs/d, LVPWs/d). The functional parameters consisted of ejection fraction (EF %) and fractional shortening (FS %), which were calculated from the structural parameters. The formulas were shown as follows.

$${\rm{EF}}=\left[\left(\frac{7}{2.4+{\rm{LVIDd}}}\times {{\rm{LVIDd}}}^{3}\right)-\left(\frac{7}{2.4+{\rm{LVIDs}}}\times {{\rm{LVIDs}}}^{3}\right)\right]\div\left(\frac{7}{2.4+{\rm{LVIDd}}}\times {{\rm{LVIDd}}}^{3}\right)\times 100 \% .$$

$${\rm{FS}}=\left({\rm{LVIDd}}-{\rm{LVIDs}}\right)\div{\rm{LVIDd}}\times 100 \% .$$

### Histological analysis and immunofluorescence staining

Mouse hearts were fixed in 4% paraformaldehyde for 48 h at 4 °C, dehydrated, and embedded in paraffin or optimal cutting temperature compound (OCT). Paraffin sections of 5-μm were used for histology analysis. Hematoxylin-eosin staining (H&E), Sirius red staining, and Masson’s trichrome staining were conducted by the Department of Pathology at Sir Run Run Shaw Hospital.

For wheat germ agglutinin (WGA) staining, 7-μm frozen sections were used. For cell size staining, cell slides were used. After washing with PBS for 3 times, sections were permeabilized with 0.1% Triton-X100 for 10 min at room temperature and blocked with 5% normal goat serum/1% BSA. Slides were incubated with primary antibodies overnight at 4 °C and the next day with secondary antibodies for 1 h at room temperature. After washing for 5 times with PBS, the sections were counterstained with DAPI. Images were then captured using a Leica TCS SP8 confocal laser scanning microscope (Leica, Solms, Germany) or an automated fluorescence microscope (Olympus, Japan).

### ELISA

Expression of HSP70 in mouse serum was measured using a commercial ELISA kit (ABIN6956430) according to the manufactory’s instructions.

### Statistical analysis

The investigators were blinded to group allocation throughout the study, including outcome assessment and statistical analysis. For in vivo experiments, sample sizes were defined by a priori power calculation with G-Power 3.1.9 software (http://www.gpower.hhu.de/), ensuring a statistical power of 80% and α = 0.05 [38]. For in vitro experiments, the sample sizes were based on field norms and the numbers needed to achieve statistical significance. The exact group size (N) for each experiment is provided and ‘N’ refers to independent biological replicates, not technical replicates.

Data presented in this study is representative of at least 3 independent experiments and is expressed as Mean ± SEM. All statistical analysis were performed with GraphPad Prism 8.0 software (San Diego, CA, USA). Shapiro-Wilk normality test (*P* < 0.05) was used to determine the adherence to a normal (Gaussian) distribution of data. The Mann–Whitney *U* test was used to analyze two-group comparisons and Kruskal–Wallis followed by Dunn post hoc multiple comparisons test for multi-group comparisons when the data failed normality test. In our study, we assumed that the data follows Gaussian distribution by relying on the central limit theorem. For differences between two groups, a two-tailed student’s *t*-test was performed. For multiple group comparisons, one-way ANOVA or two-way ANOVA or three-way ANOVA followed by Tukey post-hoc test was used. *P* < 0.05 was considered statistically significant.

## Results

### Asb10 is identified as a top downregulated gene in cardiac hypertrophy

**Fig. 1:**
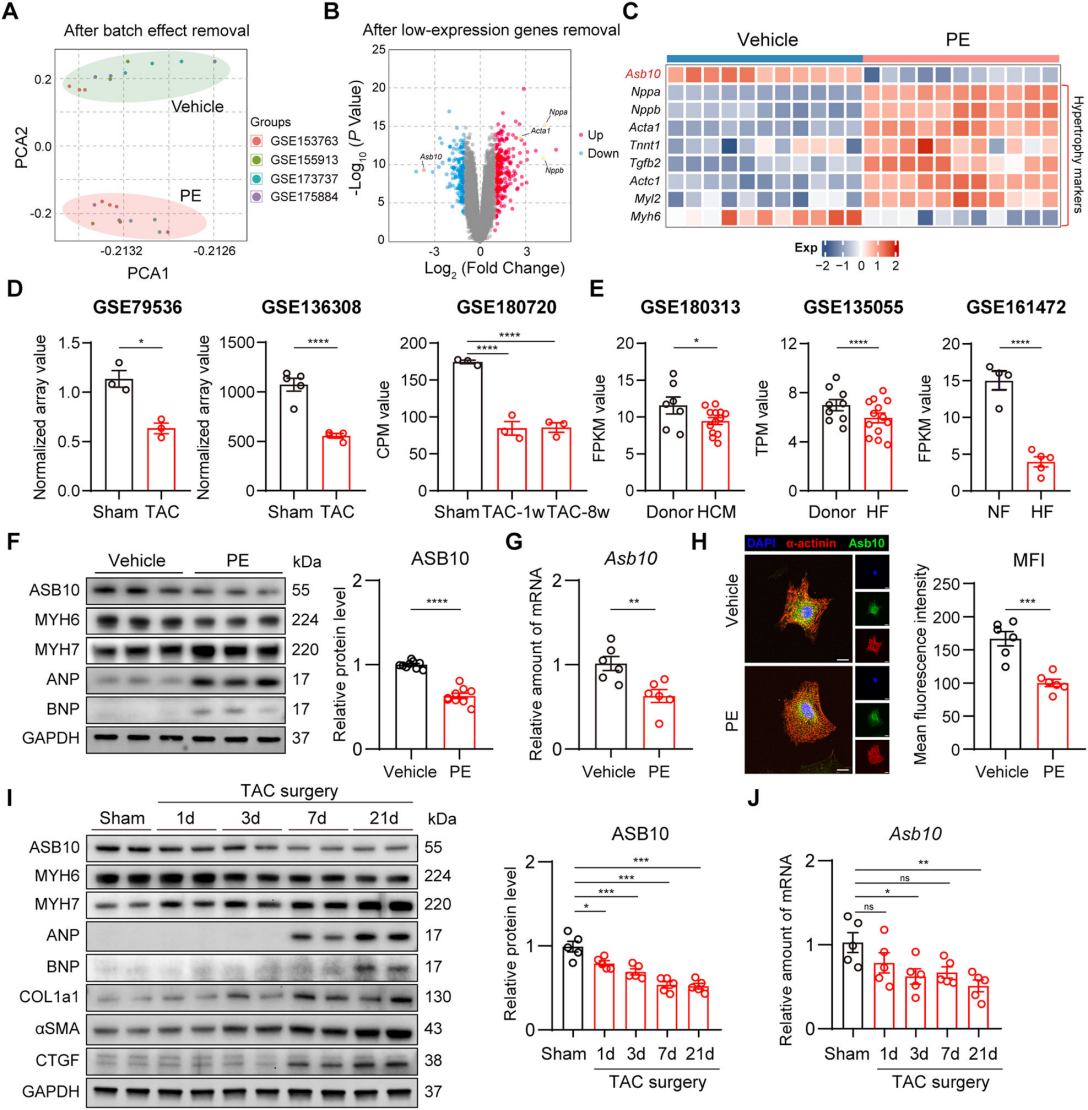
Asb10 is downregulated in pathological cardiac hypertrophy in vitro and in vivo. **A** Principal component analysis (PCA) of merged GEO datasets. *N* = 11. **B** Volcano plot of the merged GEO datasets. *N* = 11. **C** Heat map showing the relative expression of *Asb10* and hypertrophic markers in merged GEO datasets. *N* = 11. **D** Validation of the relative *Asb10* expression in cardiac tissue of sham and TAC mice from three GEO datasets, GSE79536, GSE136308 and GSE180720. *N* = 3 for GSE79536 and GSE180720, *N* = 5 (sham) and 4 (TAC) in GSE136308. **E** Validation of the relative *Asb10* expression in cardiac tissue of donors, hypertrophic cardiomyopathy and heart failure patients from three GEO datasets, GES180313, GSE135055, GSE161472. *N* = 7 (Donor) and 13 (HCM) in GSE180313, *N* = 9 (Donor) and 13 (HF) in GSE135055, *N* = 4 (NF) and 5 (HF) in GSE161472. **F** In vitro validation of the protein expression of ASB10 in NRVMs treated with 50 μM PE for 24 h as determined by western blot. The right panel showed the densitometric quantification. *N* = 9. **G** In vitro validation of the mRNA expression of *Asb10* in NRVMs treated with 50 μM PE for 24 h as determined by qPCR. *N* = 6. **H** Representative images of anti-ASB10 immunofluorescence staining in NRVMs treated with 50 μM PE for 24 h. Scale bar = 10 μm. The right panel showed the quantification. *N* = 6. **I** In vivo validation of the protein expression of ASB10 in heart tissue from mice treated with TAC surgery as determined by western blot. The right panel showed the densitometric quantification. *N* = 5. **J** In vivo validation of the mRNA expression of *Asb10* in heart tissues from mice treated with TAC surgery as determined by qPCR. *N* = 5. Limma power differential expression analysis (**B**–**E**) was conducted. Unpaired Student’s *t*-test (**F**, **G**) was conducted. One-way ANOVA followed by Turkey’s multiple comparisons test was conducted (**I**, **J**). Data were represented as Mean ± SEM. *P < 0.05, **P < 0.01, ***P < 0.001, ****P < 0.0001, ^ns^*P* > 0.05.

To determine the key genes involved in the progression of cardiac hypertrophy and heart failure, we first screened the GEO database for differentially expressed genes (DEGs) in phenylephrine (PE)-treated NRVMs. Subsequently, 4 datasets with identical experimental conditions and comparable significant readouts (Fig. S1A–D) were selected for further investigation. After removing the batch effect, 11 vehicle-treated samples and 11 PE-treated samples from 4 datasets were combined, and the two groups were well divided as shown by the PCA plot (Figs. S1E and 1A). In this merged dataset, PE-induced hypertrophy model demonstrated great quality control as most of the hypertrophic markers were significantly changed (Figs. S1F and 1B, C). *Asb10* was identified as the key gene for the following two reasons: 1) Asb10 is a heart-enriched protein (Fig. S2) with a known function as an E3 ligase [8, 16], 2) it was one of the most downregulated genes and was homogeneously downregulated in all 11 PE-treated samples (Fig. 1B, C). We then validated the expression of *Asb10* in GEO datasets acquired from mouse cardiac hypertrophic samples, human hypertrophic cardiomyopathy samples, and human dilated cardiomyopathy samples. The quality control of the validation datasets was defined as the significant changes of canonical cardiac hypertrophic and fibrotic biomarkers (Fig. S3A, B). Consistent with the in vitro data, we confirmed that *Asb10* was also downregulated in both mouse hypertrophic samples and human cardiomyopathy samples (Fig. 1D, E). Taken together, all these RNA-sequence data revealed that *Asb10* is significantly downregulated in pathological cardiac hypertrophy.

### Asb10 is downregulated in pathological cardiac hypertrophy in vitro and in vivo

We next aimed to verify whether *Asb10* was downregulated in our experimental cardiac hypertrophy models. Firstly, the specificity of Asb10 antibody was confirmed by western blot analysis of Asb10 overexpression protein samples (Fig. S4). For the in vitro cardiac hypertrophy model, NRVMs were isolated and stimulated with 50 μM PE for 24 h, as previously reported [^36^]. After treatment, cardiomyocytes were harvested for the detection of Asb10 expression. We showed that Asb10 was significantly decreased in response to PE-induced cardiac hypertrophy (Figs. 1F–H and S5A, B). In another cardiac hypertrophy model induced by ET-1 stimulation, the expression of Asb10 was also substantially downregulated (Fig. S5C, D). For the in vivo model, transverse aortic constriction (TAC) was conducted to trigger cardiac hypertrophy. Mice that received either sham or TAC surgery over 4 different time intervals were sacrificed, and the hearts were collected. The echocardiographic and biometric parameters indicated a progression from compensatory cardiac hypertrophy to heart failure over the time course (Fig. S6A, B). Consistent with the previous in vitro results, the expression of Asb10 showed a progressive decrease over the course of cardiac hypertrophy development (Figs. 1I, J and S6C, D). Collectively, these findings suggest that Asb10 is downregulated in pathological cardiac hypertrophy and heart failure.

### Overexpression of Asb10 promotes hypertrophic growth in NRVMs

**Fig. 2:**
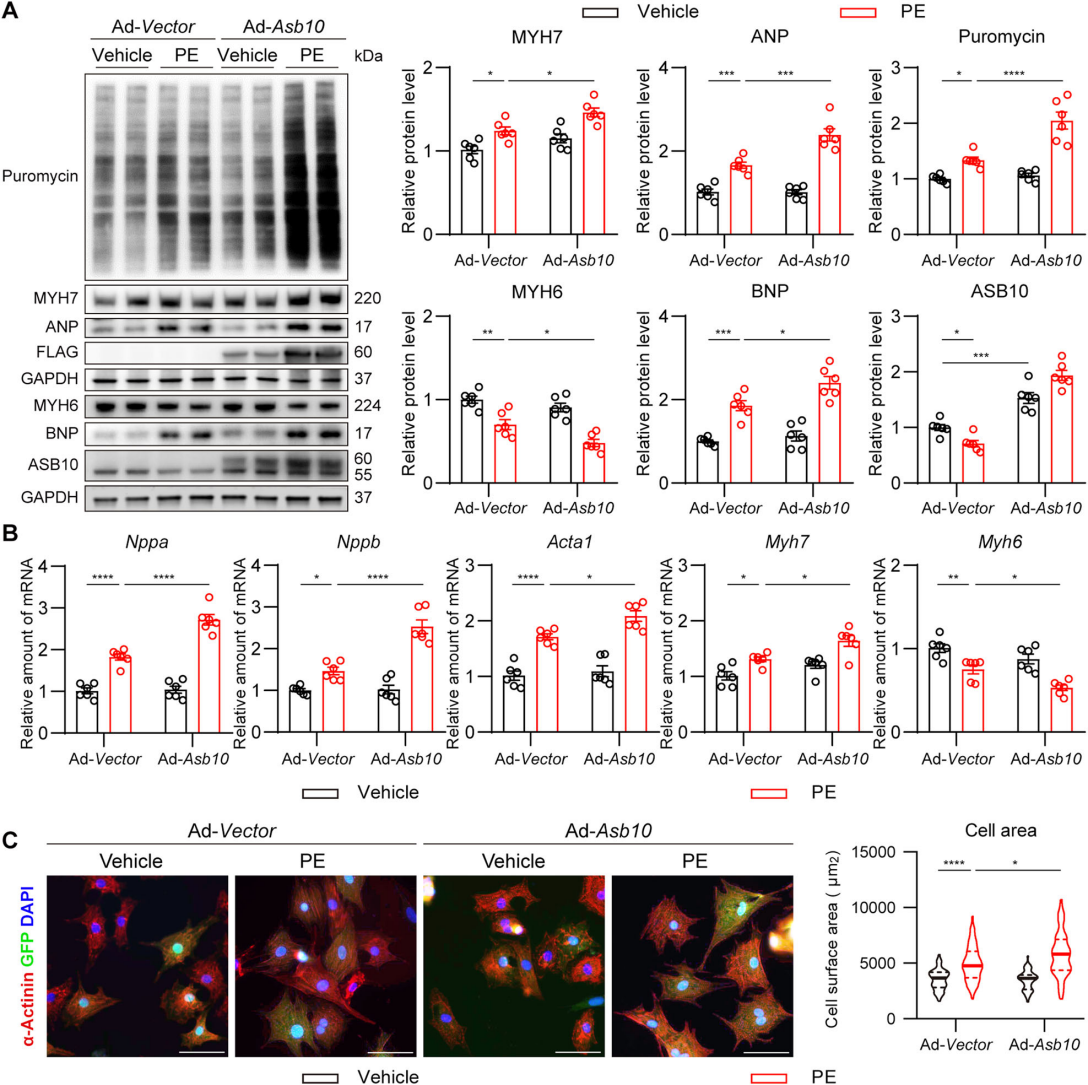
Overexpression of Asb10 deteriorates cardiac hypertrophy in NRVMs. NRVMs were infected with adenovirus-packed Asb10 or Vector for 30 h, then treated with 50 μM PE for 24 h, and cells were then collected for further detection. **A** Protein expression of hypertrophic markers with GAPDH as loading control as determined by western blot. The right panel showed the densitometric quantifications. *N* = 6. **B** Relative mRNA expressions of hypertrophic markers as determined by qPCR. *N* = 6. **C** Representative images of anti-α-actinin immunofluorescence staining showing cell size. The right panel showed the quantification. Scale bar = 30 μm. *N* = 56 (Ad-*Vector*+Vehicle), 54 (Ad-*Vector* + PE), 50 (Ad-*Asb10*+Vehicle), 53 (Ad-*Asb10* + PE). Two-way ANOVA followed by Turkey’s multiple comparisons test was conducted. Data were represented as Mean ± SEM. *P < 0.05, **P < 0.01, ***P < 0.001, ****P < 0.0001.

Since the expression of Asb10 is decreased during the progression of cardiac hypertrophy and heart failure, we asked whether overexpression of Asb10 could reverse the situation. Adenovirus-packed Asb10 (Ad-*Asb10*) was used to overexpress Asb10 in NRVMs, while the same concentration of Adenovirus (Ad-*Vector*) served as control. The optimal multiplicity of infection (MOI) was selected based on the western blot detection of FLAG-tag, and NRVMs infected with adenovirus of MOI = 10 showed a high infection rate indicated by the green fluorescence (Fig. S7). After being infected for 30 h, NRVMs were treated with PE to induce hypertrophy, and 0.5 μg/mL of puromycin was then added 2 h before the samples were collected as previously reported [39]. Unexpectedly, overexpression of Asb10 further increased the expression of puromycin-labeled proteins and cardiac hypertrophic markers after PE treatment, indicating more abundant protein synthesis and severe hypertrophic growth (Fig. 2A). This was further confirmed by qPCR measurement of hypertrophic markers and anti-α-actinin staining of cardiomyocytes (Fig. 2B, C). Besides, Asb10 overexpression also exacerbated hypertrophic growth triggered by ET-1, another commonly used drug for hypertrophic stimulation (Fig. S8). Altogether, these results indicate that Asb10 overexpression deteriorates hypertrophic growth in NRVMs.

### HSP70 is identified as a potential interacting protein of Asb10

**Fig. 3:**
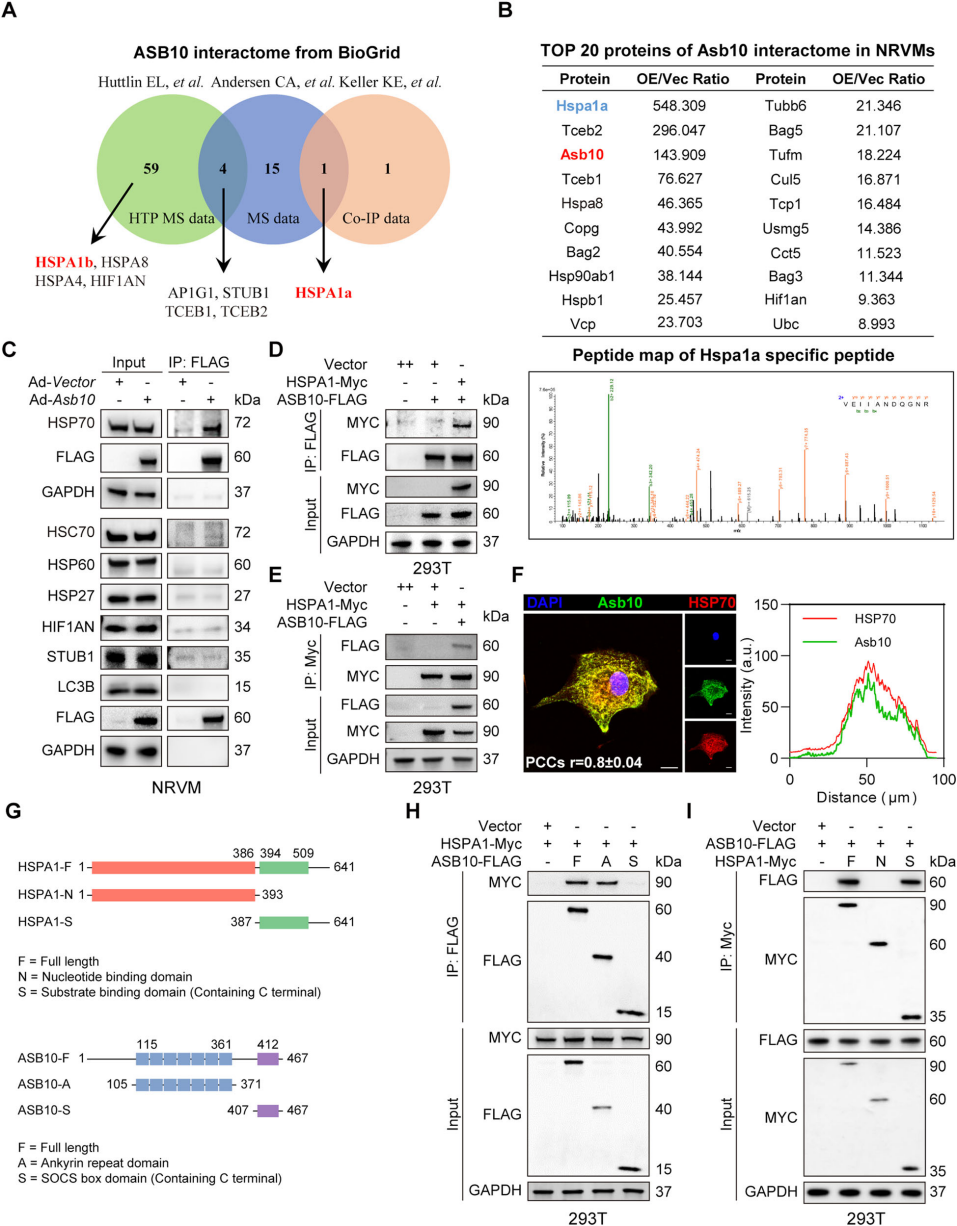
HSP70 is identified as a potential interacting protein of Asb10. **A** ASB10 interactome from BioGRID (www.thebiogrid.org). **B** Immunoprecipitation-mass spectrometry assay to screen the Asb10 interactome in NRVMs overexpressing Asb10. The top 20 interacting proteins were shown in the upper panel, and the peptide map of the HSPA1a peptide with the highest mapping score was shown in the lower panel. **C** Co-immunoprecipitation assay to validate the interacting proteins in NRVMs. *N* = 3. **D** Co-immunoprecipitation assay to validate the interaction between ASB10 and HSP70 in 293 T cells overexpressing ASB10-FLAG and HSPA1-Myc. *N* = 3. **E** Co-immunoprecipitation assay to validate the interaction between HSP70 and ASB10 in 293 T cells overexpressing HSPA1-Myc and ASB10-FLAG. *N* = 3. **F** Representative images of anti-Asb10 and anti-HSP70 immunofluorescence staining. The right panel showed the colocalization of Asb10 and HSP70. PCCs r value was shown to quantify the colocalization. Scale bar = 10 μm. *N* = 5. **G** Schematic illustration of the structure of HSPA1, ASB10 and truncated HSPA1, ASB10. **H** Co-immunoprecipitation assay to detect the binding domain of HSPA1 with ASB10 in 293 T cells overexpressing the full length of HSPA1 and truncated ASB10. *N* = 3. **I** Co-immunoprecipitation assay to detect the binding domain of ASB10 with HSPA1 in 293 T cells overexpressing the full length of ASB10 and truncated HSPA1. *N* = 3.

We next aimed to elucidate the molecular mechanism underlying the phenotype that Asb10 exacerbates cardiac hypertrophy. As a previously reported ubiquitination regulator [16], Asb10 was reported to interact with Culin-5, RBX, and Elongin BC to form an integrated E3 ligase [40] and was shown to enhance the ubiquitination of its substrates [16]. Hence, identifying the direct binding proteins of Asb10 that regulate cardiac hypertrophy may shed light on the molecular mechanism of Asb10’s actions in the heart. In this regard, a comprehensive analysis combining both reported Asb10-interactome data and our experimental immunoprecipitation-mass spectrum data of Asb10 interactome in NRVMs was performed. Several potential interacting proteins including HSP70 were identified (Fig. 3A, B, Table S2, Fig. S9). Next, to validate the interaction between candidate targets and Asb10, we performed immunoprecipitation in NRVMs and found that Asb10 directly bound to HSP70 and HSC70 (Fig. 3C). HSP70 was then selected for further tests for the following reasons: 1) our immunoprecipitation results showed that the interaction between HSP70 and Asb10 was significantly stronger than that between HSC70 and Asb10; 2) HSP70 ranked at the top of Asb10 interactome in our immunoprecipitation-mass spectrum data (Fig. 3B); 3) HSP70 was reported to be a critical regulator of pathological cardiac hypertrophy [22, 41, 42]; 4) multiple previous publications reported the interaction between HSP70 and Asb10 in other cell types [15, 40].

To further confirm the direct interaction between Asb10 and HSP70, we validated the Asb10/HSP70 complex in HEK293T cells by transfecting cells with MYC-tagged HSPA1 and FLAG-tagged ASB10 plasmids, followed by co-immunoprecipitation assays (Fig. 3D, E). In addition, immunofluorescence staining results showed clear endogenous colocalization of HSP70 and Asb10 in NRVMs (Fig. 3F), which further supports the direct interaction between Asb10 and HSP70. In addition, we constructed truncated HSPA1 and ASB10 plasmids and co-transfected them in HEK293T cells to map the domains required for ASB10 and HSP70 interaction. We demonstrated that the ankyrin repeat domain of ASB10 (ASB10-ANKR) bound to the substrate binding domain (containing C terminal) of HSP70 (HSPA1-SBD + C) (Fig. 3G–I). Taken together, these results suggest that Asb10 directly binds to HSP70.

### Asb10 stabilizes HSP70 via competitively inhibiting STUB1-mediated ubiquitination of HSP70

**Fig. 4:**
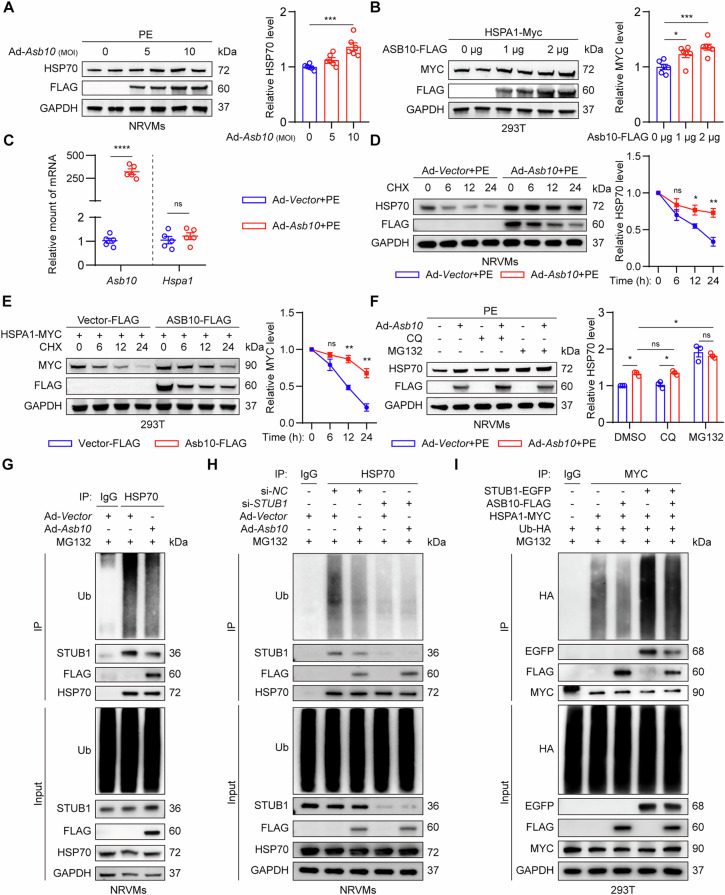
ASB10 stabilizes HSP70 via competitively inhibiting STUB1-mediated ubiquitination of HSP70. **A** Protein expression of HSP70 after overexpressing Asb10 in NRVMs as determined by western blot. The right panel showed the quantifications. *N* = 6. **B** Protein expression of HSP70 after overexpressing ASB10 in 293 T cells as determined by western blot. The right panel showed the quantifications. *N* = 6. **C** Relative expressions of *Asb10* and *Hspa1* in NRVMs overexpressing Asb10 as determined by qPCR. *N* = 5. **D** Expression changes of HSP70 in NRVMs overexpressing Asb10 and treated with different time courses of CHX. The right panel showed the quantifications. *N* = 3. **E** Expression changes of HSP70 in 293T cells overexpressing ASB10 and treated with different time courses of CHX. The right panel showed the quantifications. *N* = 3. **F** Expression changes of HSP70 in NRVMs overexpressing Asb10 and treated with CQ or MG132 for 6 h. The right panel showed the quantifications. *N* = 3. **G** Co-immunoprecipitation assay to detect the changes of the STUB1/HSP70 complex and HSP70 ubiquitination level in NRVMs overexpressing Asb10. *N* = 3. **H** Co-immunoprecipitation assay to detect the changes of the STUB1/HSP70 complex and HSP70 ubiquitination level in NRVMs transfected with STUB1 siRNA and Ad-*Asb10*. *N* = 3. **I** Co-immunoprecipitation assay to detect the changes of the STUB1/HSP70 complex and HSP70 ubiquitination level in 293T cells transfected with STUB1-EGFP, ASB10-FLAG, HSPA1-Myc and Ub-HA. *N* = 3. Unpaired Student’s *t*-test (**A**–**E**) was conducted. One-way ANOVA followed by Turkey’s multiple comparisons test (**F**) was conducted. Data were represented as Mean ± SEM. *P < 0.05, **P < 0.01, ***P < 0.001, ****P < 0.0001, ^ns^*P* > 0.05.

E3 ligase-mediated protein degradation through the ubiquitin-proteasome system is one of the most important mechanisms for cellular protein quality control [43]. Asb10’s ability to directly interact with HSP70 in NRVMs promoted us to ask whether this interaction leads to the degradation of HSP70, thereby playing a role in the pathogenesis of cardiac hypertrophy and heart failure. We assessed the post-translational and transcriptional expression of HSP70 following Asb10 overexpression. Unexpectedly, we found that overexpression of Asb10 elevated the protein level of HSP70 in both NRVMs and HEK293T cells (Fig. 4A, B), while the mRNA expression of *Hspa1* remained unaltered (Fig. 4C). By utilizing cycloheximide (CHX), a commonly used de novo protein synthesis inhibitor, we further showed that Asb10 overexpression reduced the degradation rate of HSP70 in both NRVMs and HEK293T cells (Fig. 4D, E). These results suggested that Asb10 may protect HSP70 from being degraded rather than promoting its degradation in cardiac hypertrophy. Considering that the quality control of HSP70 is mainly regulated via the ubiquitin-proteasome system and chaperon-mediated autophagy-lysosome system [18], we treated NRVMs with proteasome inhibitor MG132 and autophagy-lysosome inhibitor CQ, respectively, to investigate which signaling pathway is responsible for Asb10-mediated protection of HSP70. We showed that in NRVMs, the degradation of HSP70 was mainly mediated by the ubiquitin-proteasome system (Fig. 4F). Based on these findings, we proceeded to investigate the ubiquitination level of HSP70 in NRVMs after Asb10 overexpressing. Surprisingly, we found that the ubiquitination of HSP70 was reduced in Asb10 overexpression NRVMs (Figs. 4G and S10A), implying that Asb10 may not be the E3 ubiquitin ligase for ubiquitination of HSP70, and there may be other factors mediating the ubiquitination of HSP70. A previous study reported that STIP1 homology and U-box containing protein 1 (STUB1) is a co-chaperone protein with E3 ubiquitin ligase activity, mediating the K48-ubiquitination and proteasomal degradation of HSP70 [19]. Another study further showed that other proteins can competitively inhibit the binding of STUB1 to HSP70, thereby stabilizing HSP70 [44]. Next, we asked whether Asb10 overexpression protects HSP70 from degradation by competing with STUB1 for the same binding site of HSP70, thereby inhibiting STUB1-mediated ubiquitination and proteasomal degradation of HSP70. We first explored the binding mode of STUB1 and HSP70 by co-immunoprecipitation assay. We found that STUB1 also interacted with the HSPA1-SBD + C domain of HSP70, the same domain that Asb10 bound to (Fig. S10B). Further co-immunoprecipitation assay revealed that overexpression of Asb10 decreased the STUB1/HSP70 complex and reduced the ubiquitination of HSP70 (Figs. 4G and S10A). To further elucidate the interactions between Asb10, HSP70 and STUB1, we employed STUB1 siRNA and STUB1 overexpression plasmid to achieve both loss-of-function and gain-of-function conditions. As shown in Fig. 4H, under basal STUB1 levels, overexpression of Asb10 significantly reduced the STUB1/HSP70 complex formation and the ubiquitination of HSP70. However, when STUB1 levels were reduced, Asb10 overexpression failed to further decrease HSP70 ubiquitination (Figs. 4H and S10C). Conversely, STUB1 overexpression reversed the Asb10 overexpression-induced reduction of STUB1/HSP70 complex formation and HSP70 ubiquitination (Figs. 4I and S10D). Collectively, these results indicate that Asb10 stabilizes HSP70 via competitively inhibiting STUB1-mediated ubiquitination and degradation of HSP70.

### Downregulation of HSP70 partially rescues Asb10 overexpression-induced hypertrophic growth in NRVMs

**Fig. 5:**
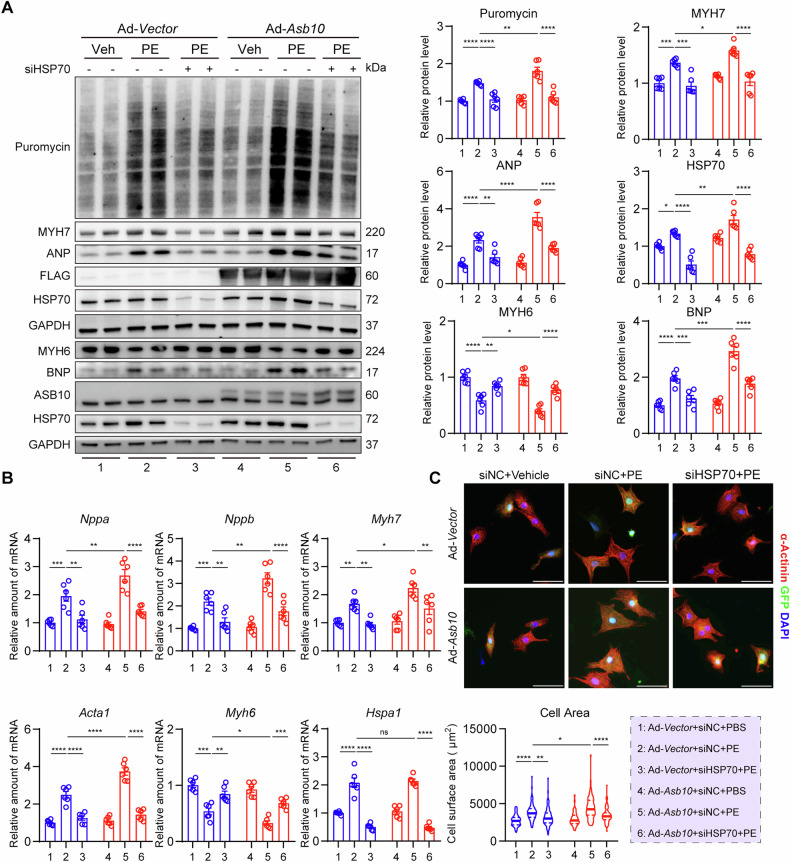
Downregulation of HSP70 partially rescues Asb10 overexpression-induced hypertrophic growth in NRVMs. NRVMs were infected with adenovirus-packed Asb10 or Vector, and co-transfected with siNC or siHSP70 for 30 h, then treated with 50 μM PE for 24 h, and cells were then collected for further detection. **A** Protein expression of hypertrophic markers with GAPDH as loading control as determined by western blot. The right panel showed the quantifications. *N* = 6. **B** Relative mRNA expression of hypertrophic markers as detected by qPCR. *N* = 6. **C** Representative images of anti-α-actinin immunofluorescence staining showing cell size. The lower panel showed the quantifications. Scale bar = 30 μm. *N* = 76 (group 1), 79 (group 2), 82 (group 3), 84 (group 4), 75 (group 5) and 80 (group 6). Three-way ANOVA followed by Turkey’s multiple comparisons test was conducted. Data were represented as Mean ± SEM. *P < 0.05, **P < 0.01, ***P < 0.001, ****P < 0.0001, ^ns^*P* > 0.05.

As mentioned above that HSP70 was reported to be a critical regulator of cardiac hypertrophy, we next set to investigate whether Asb10 overexpression-induced protection of HSP70 contributes to PE-induced hypertrophic growth in cardiomyocytes. PES, a chemical inhibitor of HSP70 [22], and siRNA oligo against HSP70 were utilized to inhibit and knockdown HSP70, respectively. Under basal condition, both HSP70 silencing and PES treatment were able to partially inhibit hypertrophic growth as revealed by the western blot analysis of puromycin-labeled proteins and cardiac hypertrophic markers (Fig. S11). After PE treatment, HSP70 knockdown ameliorated hypertrophic growth in Asb10 overexpression NRVMs, as determined by western blot, qPCR, and cell size analyses (Fig. 5). In addition, pharmacological inhibition of HSP70 by PES was also shown to attenuate PE-induced hypertrophic growth in Asb10 overexpression NRVMs (Fig. S12). These findings together suggest that HSP70 is required for Asb10 overexpression-induced hypertrophic growth in cardiomyocytes.

### Cardiac overexpression of Asb10 deteriorates pathological cardiac hypertrophy under pressure overload

**Fig. 6:**
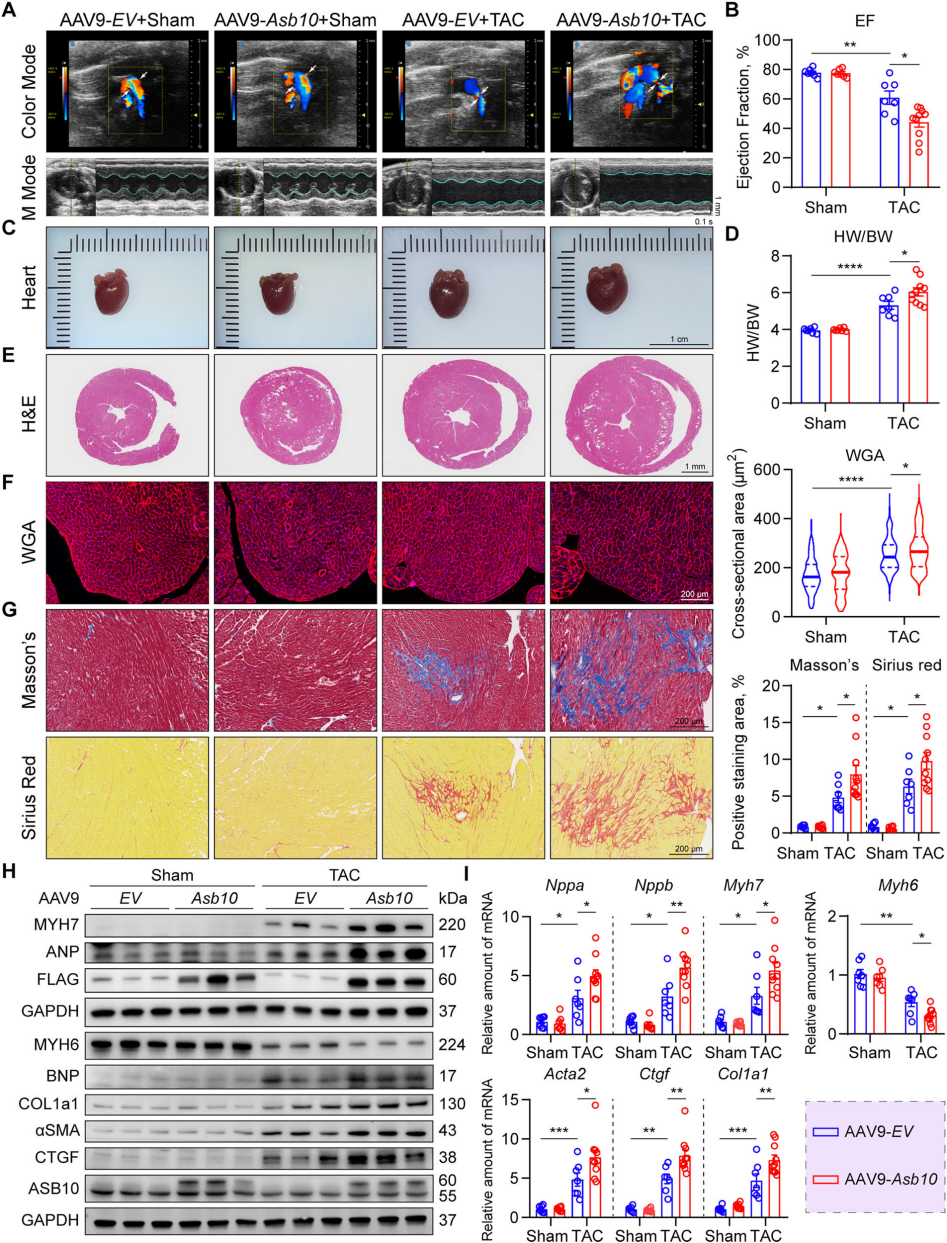
Cardiac overexpression of Asb10 deteriorates pathological cardiac hypertrophy under pressure overload. Mice were randomly divided into 4 groups: AAV9-*EV*+Sham, AAV9-*Asb10*+Sham, AAV9-*EV* + TAC and AAV9-*Asb10* + TAC. AAV9 infection was achieved via tail vein injection. Four weeks after injection, mice received either TAC or sham surgery. The cardiac function of mice was assessed using a non-invasive high-resolution imaging system. Mice were then sacrificed, and left ventricular tissues were collected for further detection. **A** Representative color mode echo images of blood flow in the aortic arch area (upper panel) and M mode images of echocardiography from 4 groups (lower panel). *N* = 7 (*EV*+Sham, *Asb10*+Sham, *EV* + TAC) and 10 (*Asb10* + TAC). **B** Ejection fraction (EF%) of mice in the late phase of TAC. *N* = 7 (*EV* + Sham, *Asb10*+Sham, *EV* + TAC) and 10 (*Asb10* + TAC). **C** Representative visual images of hearts from 4 groups. Scale bar = 1 cm. N = 7 (*EV*+Sham, *Asb10*+Sham, *EV* + TAC) and 10 (*Asb10* + TAC). **D** Heart weight and body weight ratio (HW/BW). *N* = 7 (*EV*+Sham, *Asb10*+Sham, *EV* + TAC) and 10 (*Asb10* + TAC). **E** Representative H&E staining images of mice. Scale bar = 1 mm. *N* = 7 (*EV*+Sham, *Asb10*+Sham, *EV* + TAC) and 10 (*Asb10* + TAC). **F** Representative WGA immunofluorescence staining images showing cardiomyocyte cross-sectional area. The right panel showed the quantifications. Scale bar = 200 μm. *N* = 216 (*EV*+Sham), 180 (*Asb10*+Sham), 240 (*EV* + TAC), 255 (*Asb10* + TAC). **G** Representative Masson’s and Sirius Red images showing cardiac interstitial fibrosis. The right panel showed the quantifications. Scale bar = 200 μm. *N* = 7 (*EV*+Sham, *Asb10*+Sham, *EV* + TAC) and 10 (*Asb10* + TAC). **H** Protein expression of hypertrophic and fibrotic markers with GAPDH as loading control as determined by western blot. *N* = 7 (*EV*+Sham, *Asb10*+Sham, *EV* + TAC) and 10 (*Asb10* + TAC). **I** Relative mRNA expression of pathological hypertrophic markers (upper panel) and cardiac fibrotic markers (lower panel) as detected by qPCR. *N* = 7 (*EV*+Sham, *Asb10*+Sham, *EV* + TAC) and 10 (*Asb10* + TAC). Two-way ANOVA followed by Turkey’s multiple comparisons test was conducted. Data were represented as Mean ± SEM. *P < 0.05, **P < 0.01, ***P < 0.001, ****P < 0.0001.

Our findings in NRVMs promoted us to investigate whether this pro-hypertrophy effect of Asb10 also exists in the mouse heart under hemodynamic stress. To overexpress Asb10 specifically in cardiac tissue, we took advantage of the AAV9 gene expression system [45]. An AAV9-cTNT-*Asb10* virus was constructed and injected through tail vein to achieve cardiac-specific overexpression of Asb10. Firstly, a concentration-expression curve of AAV9 was established, and the amount of 5.0E + 11 v.g. was determined to be the optimal titer by western blot and qPCR analyses. Mice injected with 5.0E + 11 v.g. of AAV9-*Asb10* for 4 weeks showed approximately a 5.0-fold elevation in mRNA levels and a 2-fold increase in protein levels (Fig. S13A, B). The overexpression of Asb10 was restricted to cardiac tissue, as nearly no signal of FLAG-tag was detected in the brain, kidney, liver, and skeletal muscle (Fig. S13C). In addition, injection of AAV9-*Asb10* for 4 weeks did not affect the basal cardiac function and structure in mice (Fig. S13D–F). To determine the role of Asb10 overexpression in the heart under pressure overload, mice were injected with 5.0E + 11 v.g. of AAV9-*Asb10* and subjected to TAC surgery. As shown by the aortic arch ultrasound analysis, TAC surgery successfully resulted in aortic arch stenosis and high blood flow velocity (Figs. 6A and S14A).

At the early postoperative time points following TAC surgery, mice in AAV9-*Asb10* group exhibited a significant increase of LVAWd and a trend of increased LVAWs and LVPWd, compared with the control group. However, no difference of cardiac function was observed between the two groups (Fig. S14B, C). After prolonged TAC surgery, echocardiography analysis manifested that Asb10 overexpression led to a significant increase in LVIDs and a dramatic decrease in both ejection fraction and fractional shortening, indicating more severe systolic dysfunction compared with the control group (Figs. 6A, B and S15A). No change in heart rate was detected (Fig. S15A). While no morphological changes were seen at baseline between AAV9-*Asb10* and control groups, prolonged hemodynamic stress resulted in enlarged hearts and elevated ratios of heart weight to body weight and heart weight to tibia length in the Asb10 overexpression group (Fig. 6C–E). Furthermore, histological analysis of wheat germ agglutinin staining showed a significant increase in cardiomyocyte cross-sectional area, and Masson’s trichrome staining together with Sirius Red staining revealed increased fibrosis in Asb10 overexpressed hearts (Fig. 6F, G). Additionally, molecular markers of cardiac hypertrophy and fibrosis were found to be substantially elevated by Asb10 overexpression after TAC (Fig. 6H, I). Taken together, these findings suggest that cardiac overexpression of Asb10 aggravates cardiac hypertrophy in response to hemodynamic stress.

### Increased cardiac inflammation and HDAC2 phosphorylation are involved in Asb10 overexpression-induced pathological cardiac remodeling

**Fig. 7:**
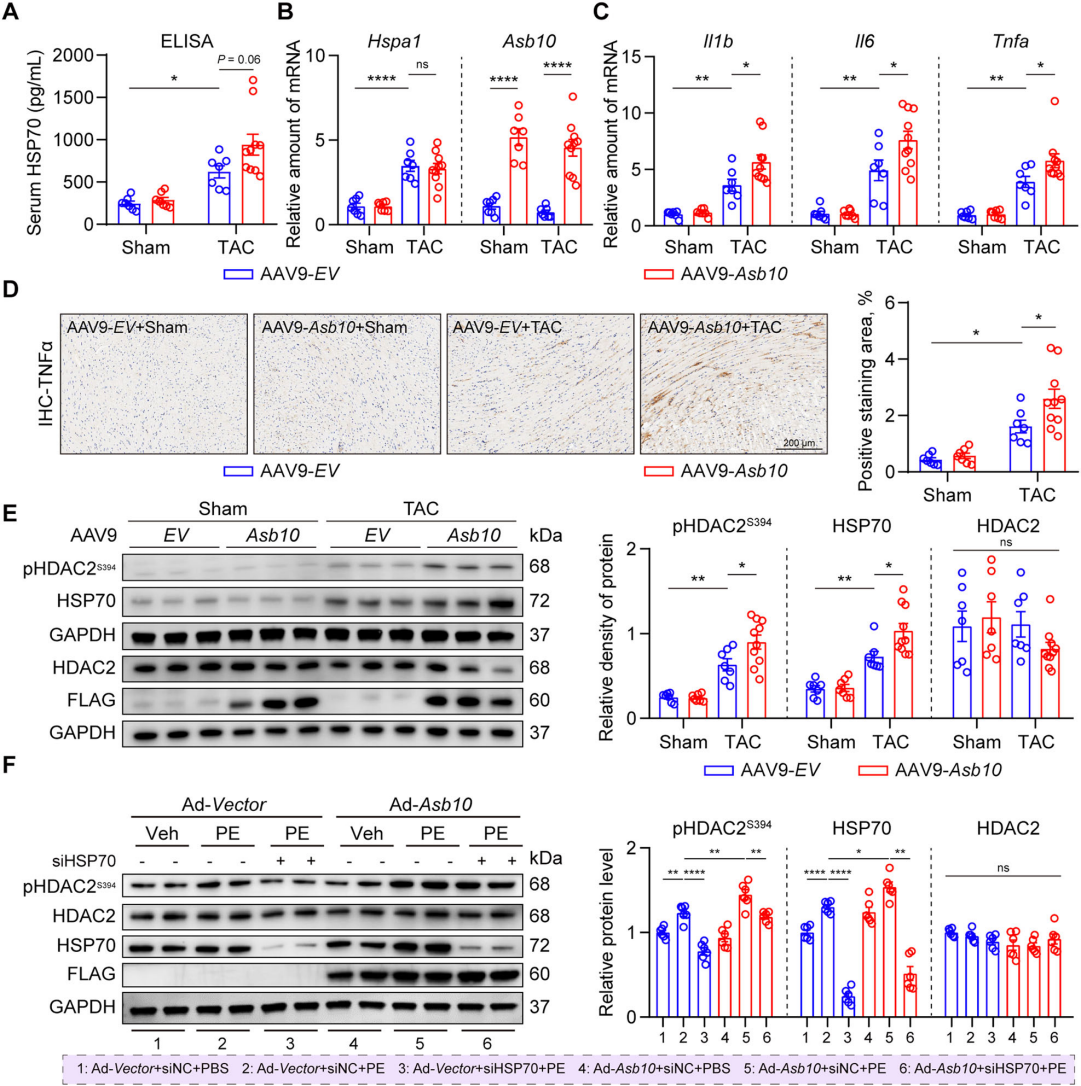
Increased cardiac inflammation and HDAC2 phosphorylation are involved in Asb10 overexpression-induced pathological cardiac remodeling. **A** ELISA assay to detect serum HSP70 levels. *N* = 7 (*EV*+Sham, *Asb10*+Sham, *EV* + TAC) and 10 (*Asb10* + TAC). **B** Relative mRNA expression of *Hspa1* and *Asb10* as detected by qPCR. *N* = 7 (*EV*+Sham, *Asb10*+Sham, *EV* + TAC) and 10 (*Asb10* + TAC). **C** Relative mRNA expression of canonical inflammation markers as detected by qPCR. *N* = 7 (*EV*+Sham, *Asb10*+Sham, *EV* + TAC) and 10 (*Asb10* + TAC). **D** Representative immunohistochemistry staining images showing cardiac inflammation of mouse hearts. The right panel showed the quantifications. Scale bar = 200 μm. *N* = 7 (*EV*+Sham, *Asb10*+Sham, *EV* + TAC) and 10 (*Asb10* + TAC). **E** Protein expression of pHDAC2^S394^, HSP70 and HDAC2 in left ventricular tissue of mice, with GAPDH as loading control as determined by western blot. The right panel showed the quantifications. *N* = 7 (*EV*+Sham, *Asb10*+Sham, *EV* + TAC) and 10 (*Asb10* + TAC). **F** Protein expression of pHDAC2^S394^, HSP70 and HDAC2 in Asb10^OE^ and HSP70^KD^ NRVMs treated with PE, with GAPDH as loading control as determined by western blot. The right panel showed the quantifications. *N* = 6. Two-way ANOVA followed by Turkey’s multiple comparisons test (**A**–**E**) was conducted. Three-way ANOVA followed by Turkey’s multiple comparisons test (**F**) was conducted. Data were represented as Mean ± SEM. *P < 0.05, **P < 0.01, ****P < 0.0001, ^ns^*P* > 0.05.

We have shown that Asb10 stabilizes HSP70 and promotes cardiac hypertrophic growth in NRVMs. We then asked if this mechanism also operates in mice overexpressing Asb10. It was worth noting that HSP70 was reported to be a double-edged sword in the pathogenesis of cardiac hypertrophy by serving as either a protective intracellular chaperon or destructive extracellular DAMPs [21–23,41]. Moreover, previous studies revealed that elevated serum level of HSP70 aggravates cardiac fibrosis and dysfunction through activation of cardiac inflammation and pro-hypertrophy pathways [41]. We assessed the serum levels of HSP70 utilizing a commercially available ELISA kit and found that circulating HSP70 was significantly increased in both AAV9-*Asb10* and AAV9-*EV* groups after TAC (Fig. 7A). Although no statistical significance was observed, there was a trend of increased serum HSP70 in the Asb10 overexpression group compared with the control group (*P* = 0.06, Fig. 7A), while the mRNA level of HSP70 remained unaltered (Fig. 7B), indicating a pro-hypertrophic role of HSP70 in the heart. Furthermore, consistent with previous findings [41], our data also revealed increased cardiac inflammation in Asb10/HSP70 axis-activated hearts (Fig. 7C, D). In addition to cardiac inflammation, HSP70-mediated phosphorylation of the S394 site of HDAC2 was reported to contribute to the development of cardiac hypertrophy [22]. Here, we detected the levels of pHDAC2^S394^, HDAC2, and HSP70 in cardiac tissues. Following TAC surgery, both HSP70 and pHDAC^S394^ were significantly upregulated in Asb10 overexpressed hearts (Fig. 7E). Consistently, Asb10 overexpression also substantially increased the expression levels of HSP70 and pHDAC2^S394^ in NRVMs, which were significantly inhibited by HSP70 knockdown (Fig. 7F). Collectively, these results suggest that HSP70 may contribute to Asb10 overexpression induced cardiac hypertrophy by increasing cardiac inflammation and phosphorylation of HDAC2.

### Cardiac-specific Asb10 knockdown ameliorates pathological hypertrophy and reduces HDAC2 phosphorylation in response to pressure overload

Since Asb10 overexpression exacerbates pathological hypertrophy upon hemodynamic stress, we next investigated whether Asb10 inhibition could mitigate this condition. To achieve cardiac-specific knockdown of Asb10, we constructed an AAV9-cTNT-*shAsb10* virus and delivered it via tail vein injection. Mice injected with 5.0E + 11 v.g. of AAV9-*shAsb10* for four weeks exhibited an approximately 60% reduction in Asb10 protein levels (Fig. S16A). This knockdown was specific to cardiac tissue, with no detectable changes in Asb10 expression in the liver, kidney, brain, or skeletal muscle (Fig. S16B). Additionally, AAV9-*shAsb10* injection for four weeks did not affect baseline cardiac function (Fig. S16D).

**Fig. 8:**
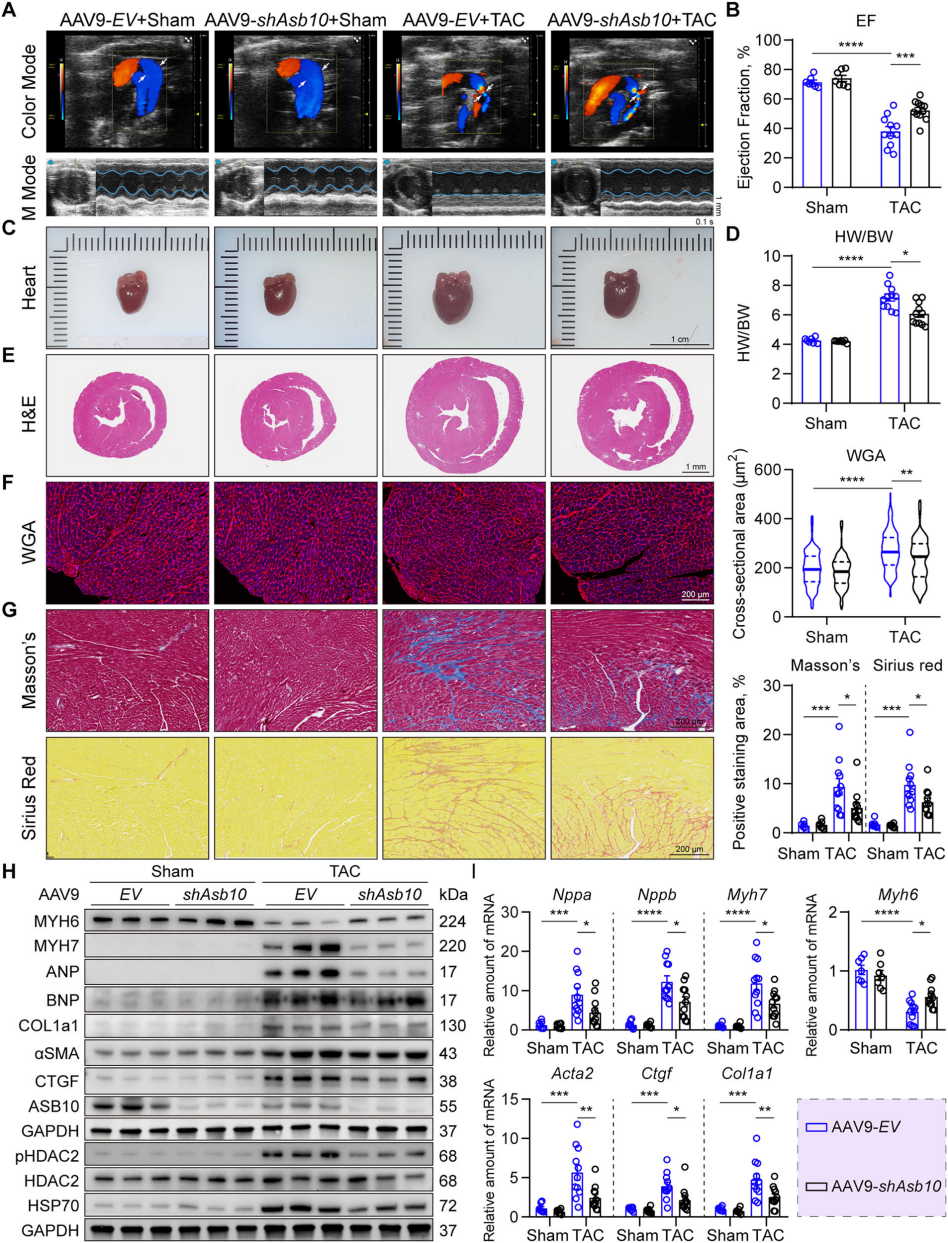
Cardiac-specific Asb10 knockdown ameliorates pathological hypertrophy and reduces HDAC2 phosphorylation in response to pressure overload. Mice were randomly divided into 4 groups: AAV9-*EV*+Sham, AAV9-*shAsb10*+Sham, AAV9-*EV* + TAC and AAV9- *shAsb10* + TAC. AAV9 infection was achieved via tail vein injection. Four weeks after injection, mice received either TAC or sham surgery. The cardiac function of mice was assessed using a non-invasive high-resolution imaging system. Mice were then sacrificed, and left ventricular tissues were collected for further detection. **A** Representative color mode echo images of blood flow in the aortic arch area (upper panel) and M mode images of echocardiography from 4 groups (lower panel). *N* = 7 (*EV*+Sham, *shAsb10*+Sham) and 11 (*EV* + TAC, *shAsb10* + TAC). **B** Ejection fraction (EF%) of mice after TAC surgery. N = 7 (*EV*+Sham, *shAsb10*+Sham) and 11 (*EV* + TAC, *shAsb10* + TAC). **C** Representative visual images of hearts from 4 groups. Scale bar = 1 cm. *N* = 7 (*EV*+Sham, *shAsb10*+Sham) and 11 (*EV* + TAC, *shAsb10* + TAC). **D** Heart weight and body weight ratio (HW/BW). *N* = 7 (*EV*+Sham, *shAsb10*+Sham) and 11 (*EV* + TAC, *shAsb10* + TAC). **E** Representative H&E staining images of mice. Scale bar = 1 mm. *N* = 7 (*EV*+Sham, *shAsb10*+Sham) and 11 (*EV* + TAC, *shAsb10* + TAC). **F** Representative WGA immunofluorescence staining images showing the cardiomyocyte cross-sectional area. The right panel showed the quantifications. Scale bar = 200 μm. *N* = 217 (*EV*+Sham), 208 (*shAsb10*+Sham) and 256 (*EV* + TAC, *shAsb10* + TAC). **G** Representative Masson’s and Sirius Red images showing cardiac interstitial fibrosis. The right panel showed the quantifications. Scale bar = 200 μm. *N* = 7 (*EV*+Sham, *shAsb10*+Sham) and 11 (*EV* + TAC, *shAsb10* + TAC). **H** Protein expression of hypertrophic and fibrotic markers with GAPDH as loading control as determined by western blot. *N* = 7 (*EV*+Sham, *shAsb10*+Sham) and 11 (*EV* + TAC, *shAsb10* + TAC). **I** Relative mRNA expression of pathological hypertrophic markers (upper panel) and cardiac fibrotic markers (lower panel) as detected by qPCR. *N* = 7 (*EV*+Sham, *shAsb10*+Sham) and 11 (*EV* + TAC, *shAsb10* + TAC). Two-way ANOVA followed by Turkey’s multiple comparisons test was conducted. Data were represented as mean ± SEM. *P < 0.05, **P < 0.01, ***P < 0.001, ****P < 0.0001.

Mice injected with AAV9-*EV* or AAV9-*shAsb10* were then subjected to TAC surgery, and both groups exhibited successful aortic arch stenosis and similarly high blood flow velocity (Fig. 8A). However, Asb10 knockdown significantly preserved cardiac systolic function compared to the control mice following TAC surgery (Figs. 8A, B and S17A). Consistently, Asb10 knockdown remarkably reduced pressure overload-induced cardiac enlargement, as well as the heart weight to body weight and heart weight to tibia length ratios (Figs. 8C, D and S17A). Although aortic pressure overload led to increased cardiomyocyte cross-sectional area and collagen deposition, Asb10 inhibition substantially alleviated the above pathological changes, as evidenced by WGA staining, Masson’s trichrome staining, and Sirius Red staining (Fig. 8F, G). Furthermore, the expression levels of hypertrophic and fibrotic molecular markers were critically reduced in Asb10 knockdown group following TAC (Figs. 8H, I and S17B). Next, we asked if cardiac Asb10 inhibition affects the expression levels of HSP70 and HDAC2 phosphorylation, as well as cardiac inflammation. In contrast to the findings in Asb10-overexpressing mice, we detected a significant reduction of HSP70 protein levels in Asb10-knockdown mice, despite no changes at its transcriptional level (Figs. 8H and S17B). Additionally, Asb10 knockdown diminished HDAC2 phosphorylation (Figs. 8H and S17B) and cardiac inflammation (Fig. S17C) following hemodynamic stress. Taken together, these findings suggest that cardiac-specific Asb10 knockdown ameliorates pathological hypertrophy and reduces HDAC2 phosphorylation in response to hemodynamic stress.

## Discussion

Hypertension is a major independent risk factor for heart failure, with a prevalence of more than 30% in the adult population worldwide [46]. Hemodynamic stress is the main characteristic of the disease. To overcome the stress and maintain its pump function, the heart is required to undergo an adaptive hypertrophic growth process. However, as the hemodynamic stress persists, it will ultimately lead to maladaptive cardiac hypertrophy and heart failure [47]. During hypertrophic growth, cardiomyocytes undergo enlargement, manifesting as an increase in sarcomere units and remodeling of microtubule networks in response to increased ventricular wall stress [1]. At the molecular level, this is paralleled by the enhancement of intracellular protein synthesis and degradation turnover [39]. As an important component of the protein quality control system, the ubiquitin-proteasome system is mainly responsible for the clearance of misfolded and oxidatively damaged proteins [48] and is believed to be crucial for the development of pathological cardiac hypertrophy [49]. In this study, we reported that Asb10 plays a critical role in the pathogenesis of cardiac hypertrophy and heart failure. We identified Asb10 as a downregulated gene in the failing hearts. Cardiac overexpression of Asb10 with AAV9-cTNT-*Asb10* exacerbates pathological cardiac remodeling in response to pressure overload, whereas Asb10 knockdown ameliorates it. Our data further showed that Asb10 prevents HSP70 from undergoing STUB1-mediated ubiquitination and degradation, and HSP70-associated cardiac inflammation and HDAC2 phosphorylation may contribute to Asb10 overexpression-induced pathological cardiac hypertrophy and heart failure.

Currently, our knowledge of Asb10 in cardiovascular disease remains limited. Asb10 was initially identified as a susceptibility gene for open-angle glaucoma (POAG), which is characterized by elevated intraocular pressure. Mutations in the N-terminal region of Asb10 have been widely identified in American, European, and Asian populations [8, 14, 50]. Further studies revealed its colocalization with HDAC6 and HSP70 in aggresome-like structures, indicating its role in proteostasis regulation [15]. A recent study by Xu et al. demonstrated that Asb10 mediates the ubiquitination and degradation of TEM8 in triple-negative breast cancer [16]. Here, we extended the role of Asb10 to cardiovascular disease. We found that Asb10 is highly enriched in cardiac tissue, as previously reported by RNA-sequencing data [17]. Our data further revealed that Asb10 is downregulated in cardiac hypertrophy and heart failure, whereas its overexpression results in more severe pathological cardiac remodeling following pressure overload, indicating its protective role in these processes. Here, we propose that the downregulation of Asb10 may function as a protective mechanism against pressure overload by attenuating excessive hypertrophic growth, thereby preserving cardiac function. Supporting this hypothesis, our data further demonstrate that Asb10 knockdown reduces cardiomyocyte cross-sectional area and alleviates cardiac dysfunction in mice following TAC surgery. The discrepancy between Asb10’s correlation and phenotype is not uncommon in cardiovascular research, and such discrepancies have been reported by many other investigators before. For example, Ferdous et al. reported that the activation of FoxO1 (p-FoxO1) is downregulated after TAC surgery, and blocking FoxO1 signaling via FoxO1-cKO preserves cardiac function in response to hemodynamic stress [51]. Additionally, another study by Barbara Roman et al. found that MICU3 expression is reduced in failing human hearts, while overexpression of MICU3 leads to more severe cardiac hypertrophy and cardiac dysfunction [52].

Ubiquitylation, a major post-translational regulatory modification in all eukaryotic cells, is defined as the covalent attachment of ubiquitin to lysine residues of a protein substrate. Briefly, in a multi-enzymatic cascade, ubiquitin molecules are sequentially activated by E1 enzymes, transferred to E2 enzymes, and covalently bound to the substrate with the assistance of E3 enzymes. This process is reversibly regulated by deubiquitinating enzymes, thereby maintaining a balance in protein quality control [48]. Recent studies highlighted the critical role of protein ubiquitination in cardiac hypertrophy. Zhao et al. demonstrated that targeting E3 ubiquitin ligase WWP1 with AAV9-shRNA prevents cardiac hypertrophy through destabilizing DVL2 via inhibition of its K27-linked ubiquitination [53]. Another study by Liu et al. revealed that E3 ubiquitin ligase TRIM16 negatively regulates cardiac hypertrophic growth via ubiquitin-dependent degradation of Src and subsequent suppression of Prdx1-dependent oxidative stress [7]. In our study, we identified HSP70 as a downstream binding protein of Asb10 by utilizing high-throughput immunoprecipitation-mass spectrometry, combined with the analysis of several reported Asb10 interactomes and co-immunoprecipitation experimental validations. Cardiac overexpression of Asb10 was found to exacerbate pathological remodeling by stabilizing HSP70. Moreover, we identified that the ankyrin repeats domain of Asb10 is the binding site for the substrate-binding domain (containing the C-terminal) of HSP70. In addition to HSP70, other heat shock proteins and chaperones, including HSC70, were also detected in our experimental Asb10 interactomes. However, we failed to detect any interaction of these potential targets with Asb10 in our experimental immunoprecipitation assay, except for HSC70. HSC70 was reported to be closely associated with the development of cardiac hypertrophy [54, 55]; however, it is primarily involved in chaperone-mediated autophagy and lysosomal degradation, not ubiquitin-mediated degradation [56], and its binding strength with Asb10 is 10-fold weaker than that of HSP70 with Asb10. As a result, HSC70 was not selected for further investigation in the following experiments.

The quality control of HSP70 has been extensively investigated under both physiological and pathological conditions. Under stress conditions, the activation of heat shock transcription factor 1 (HSF1) triggers the elevation of several heat shock proteins, including HSP70, to promote cell survival. During stress recovery, the elevated HSP70 decreases to normal levels to maintain physiological cellular homeostasis [57]. STUB1 has been identified as the typical chaperone-associated E3 ligase that mediates the sequential ubiquitination of HSP70’s clients and HSP70 itself [19]. Further study revealed six ubiquitination residues of HSP70 and the forms of polyubiquitin chains mediated by STUB1 [58]. Subsequent degradation of HSP70 by 26 s proteasome has also been observed [59]. In our study, we also demonstrated that under hypertrophic stimulation, HSP70 is mainly degraded via the STUB1-mediated UPS pathway rather than the autophagy-lysosome pathway by utilization of proteasome inhibitor and autophagy-lysosome inhibitor. Moreover, we observed a significant increase in HSP70 expression and a decrease in HSP70 ubiquitination levels following Asb10 overexpression, strongly indicating that Asb10 may prevent HSP70 from STUB1-mediated ubiquitination and degradation. Remarkably, previous studies showed that OLA1 (Obg-like ATPase-1) could competitively inhibit the binding of STUB1 to HSP70. Overexpression of OLA1 inhibits STUB1-mediated ubiquitin-dependent degradation of HSP70, while OLA1 deficiency enhances this process [44, 60]. Similar as the previous findings, our co-immunoprecipitation data also suggest that Asb10 can compete with STUB1 for the same binding domain of HSP70, thereby suppressing STUB1-mediated degradation of HSP70.

Our further results from HSP70 knockdown and HSP70 inhibitor treatments in NRVMs revealed that the pro-hypertrophic effect of Asb10 is mediated by the elevation of HSP70. Moreover, in TAC mice, we observed not only cardiomyocyte hypertrophy but also fibrotic and inflammatory responses. Given the low expression of Asb10 in immune cells and fibroblasts [17], and the specificity of Asb10 expression modulation via AAV9 virus with the cTNT promoter, we propose that the fibrotic and inflammatory responses might be concomitant phenotypes indirectly regulated by Asb10 and potentially linked to the elevation of HSP70. In fact, the role of HSP70 in cardiac hypertrophy and heart failure has been extensively investigated. Previous studies reported that hypertrophic stimuli, including aortic banding, excessive isoproterenol and angiotensin II, induce the expression of HSP70, which then interacts with HDAC2 and promotes its activity to simulate cardiomyocyte hypertrophy. Further investigations revealed that HSP70 preferentially binds to pHDAC2^S394^, but not to the S422/424 sites, and prevents the dephosphorylation of HDAC2 by reducing its interaction with PP2CA, thereby promoting the increase of pHDAC2^S394^ [22, 23]. Another study by Cai et al. further found that antagonism of extracellular HSP70 attenuates abdominal aortic constriction-induced cardiac hypertrophy and fibrosis [41]. In our study, we found an elevation of HSP70 upon pressure overload in vivo and molecular hypertrophic stimuli in vitro, with an even higher expression following Asb10 overexpression. Increased levels of HSP70 are paralleled by the activation of pHDAC2^S394^ and the deterioration of cardiac inflammation, two important factors contributing to the development of pathological cardiac hypertrophy and heart failure. Although our data, together with the findings by others mentioned above, seem to support a pro-hypertrophic role of HSP70, it is worth noting that results from an HSP70 whole-body knockout mouse model demonstrated that HSP70 deletion results in mild hypertrophy under basal conditions [42], suggesting a complex and multifaced role of HSP70 in the pathogenesis of cardiac hypertrophy. Future studies are warranted to fully address the role of HSP70 in Asb10 overexpression-mediated cardiac hypertrophy by utilizing cardiac-specific HSP70 KO mice.

There are some limitations in this study. First, due to the constraints of AAV9 technology and the absence of in vivo dual validation through knockdown and rescue experiments for both upstream and downstream proteins, further validation is required in future studies using Asb10 and HSP70 knockout or transgenic overexpression mice. Additionally, the competitive inhibition of the STUB1/HSP70 complex is a non-canonical function of Asb10 as an E3 ligase. Further investigations are needed to determine whether Asb10 regulates cardiac hypertrophy through its canonical role as an E3 ligase.

In conclusion, we found that Asb10’s expression is substantially decreased in both NRVMs and mouse hearts upon hypertrophic stimuli. Overexpression of Asb10 aggravates pressure overload-induced pathological cardiac hypertrophy by directly interacting with HSP70 and competitively inhibiting STUB1-mediated ubiquitin-dependent degradation of HSP70. In contrast, Asb10 knockdown reduces HSP70’s levels and provides protection against hemodynamic stress-induced pathological cardiac remodeling. Our study extends the understanding of the mechanisms underlying cardiac hypertrophy and provides a potential strategy of targeting the Asb10/HSP70 axis for the treatment of cardiac hypertrophy and heart failure.

## Supplementary information

Supplementary Information

Checklist

Original Gel Data

## Data Availability

The main data supporting the findings of this study are available within the article and its Supplementary Information. All GEO datasets analyzed in this study are publicly accessible with at https://www.ncbi.nlm.nih.gov/geo. The raw mass spectrometry data reported in this paper has been deposited in the Integrated Proteome Resources (iProX) with the ID of PXD061568 (http://proteomecentral.proteomexchange.org/cgi/GetDataset?ID=PXD061568).
