# Supplementary material for: Asb10 accelerates pathological cardiac remodeling by stabilizing HSP70
Source: Cell Death Dis. 2025 May 22;16(1):409. doi: 10.1038/s41419-025-07735-5 (PMC12095639; doi:10.1038/s41419-025-07735-5)
Supplement: Supplementary file 1 — Supplementary Information [file 41419_2025_7735_MOESM1_ESM.docx]

***Supplementary Materials***

**Asb10 Accelerates Pathological Cardiac Remodeling by Stabilizing HSP70**

**Supplementary Table S1.** Primer sequence used in this study.

| **Gene** | **Species** | **Sequence** | **Test** |
| --- | --- | --- | --- |
| *Asb10* | Rat | GGATGTGGGCTCTGTCTCTCAA/GGATGTGGGCTCTGTCTCTCAA | qPCR |
| *Nppa* | Rat | CTTCTTCCTCTTCCTGGCCT/TTCATCGGTCTGCTCGCTCA | qPCR |
| *Nppb* | Rat | TCCTTAATCTGTCGCCGCTG/AGGCGCTGTCTTGAGACCTA | qPCR |
| *Myh7* | Rat | CTGAGGAGACACAGCGTTCT/GGGTCAGCTGAGAGATAAGA | qPCR |
| *Myh6* | Rat | CCGAGTCCCAGGTCAACAAG/ TCATCGTGCATTTTCTGCTTGG | qPCR |
| *Acta1* | Rat | GAGCGTGGCTATTCCTTCGT/CCGCAGACTCCATACCGATAA | qPCR |
| *Hspa1* | Rat | CCGCTGTCGCTGGGTCTG/CCGAGTAGGTGGTGAAGGTCTG | qPCR |
| *Actb* | Rat | GCAGGAGTACGATGAGTCCG/ACGCAGCTCAGTAACAGTCC | qPCR |
| *18s* | Rat | AAACGGCTACCACATCCAAG/CCTCCAATGGATCCTCGTTA | qPCR |
| *Asb10* | Mouse | CTATCATCATCCGCTCGGCTT/CAGAGACCAGAGTCTCAAAGCA | qPCR |
| *Nppa* | Mouse | GCTTCCAGGCCATATTGGAG/GGGGGCATGACCTCATCTT | qPCR |
| *Nppb* | Mouse | GAGGTCACTCCTATCCTCTGG/GCCATTTCCTCCGACTTTTCTC | qPCR |
| *Myh7* | Mouse | ACTGTCAACACTAAGAGGGTCA/TTGGATGATTTGATCTTCCAGGG | qPCR |
| *Myh6* | Mouse | GCCCAGTACCTCCGAAAGTC/GCCTTAACATACTCCTCCTTGTC | qPCR |
| *Col1a1* | Mouse | CTCGTGGATTGCCTGGAACA/CCAACAGCACCATCGTTACC | qPCR |
| *Ctgf* | Mouse | TGCACTTGCCTGGATGG/GGCAGTTGGCTCGCATCATA | qPCR |
| *Acta2* | Mouse | GTCCCAGACATCAGGGAGTAA/TCGGATACTTCAGCGTCAGGA | qPCR |
| *Hspa1* | Mouse | TGGTGCAGTCCGACATGAAG/GCTGAGAGTCGTTGAAGTAGGC | qPCR |
| *Il1b* | Mouse | GCAACTGTTCCTGAACTCAACT/ATCTTTTGGGGTCCGTCAACT | qPCR |
| *Il6* | Mouse | TAGTCCTTCCTACCCCAATTTCC/TTGGTCCTTAGCCACTCCTTC | qPCR |
| *Tnfa* | Mouse | CCCTCACACTCAGATCATCTTCT/GCTACGACGTGGGCTACAG | qPCR |
| *Actb* | Mouse | GGCTGTATTCCCCTCCATCG/CCAGTTGGTAACAATGCCATGT | qPCR |
| *18s* | Mouse | AGGGTTCGATTCCGGAGAGG/CAACTTTAATATACGCTATTGG | qPCR |

**Supplementary Table S2.** Sequences of HSPA1a-specific peptides and their scores identified in mass spectrometry assay.

| **Sequence** | **Score** |
| --- | --- |
| VEIIANDQGNR | 183.47 |
| ATAGDTHLGGEDFDNR | 171.32 |
| IINEPTAAAIAYGLDR | 171.29 |
| ITITNDKGR | 157.58 |
| VCNPIISGLYQGAGAPGAGGFGAQAPK | 154.69 |
| ARFEELCSDLFR | 154.66 |
| LVSHFVEEFKR | 147.91 |
| AQIHDLVLVGGSTR | 146.79 |
| EIAEAYLGHPVTNAVITVPAYFNDSQR | 144.83 |
| TTPSYVAFTDTER | 143.50 |
| FEELCSDLFR | 141.48 |
| ITITNDK | 139.27 |
| CQEVISWLDSNTLAEKEEFVHK | 137.12 |
| FELSGIPPAPR | 135.79 |
| NQVALNPQNTVFDAK | 131.08 |
| FGDPVVQSDMK | 127.76 |
| DAGVIAGLNVLR | 126.24 |
| DNNLLGR | 122.15 |
| YKAEDEVQR | 117.70 |
| LLQDFFNGR | 112.17 |
| GGSGSGPTIEEVD | 107.69 |
| LVSHFVEEFK | 102.52 |
| KFGDPVVQSDMK | 88.02 |
| SAVEDEGLKGK | 84.21 |
| CQEVISWLDSNTLAEKEEFVHKR | 79.69 |
| SFYPEEISSMVLTK | 73.44 |
| SENVQDLLLLDVAPLSLGLETAGGVMTALIK | 54.00 |
| MKEIAEAYLGHPVTNAVITVPAYFNDSQR | 42.59 |
| KTAIGIDLGTTYSCVGVFQHGK | 36.66 |


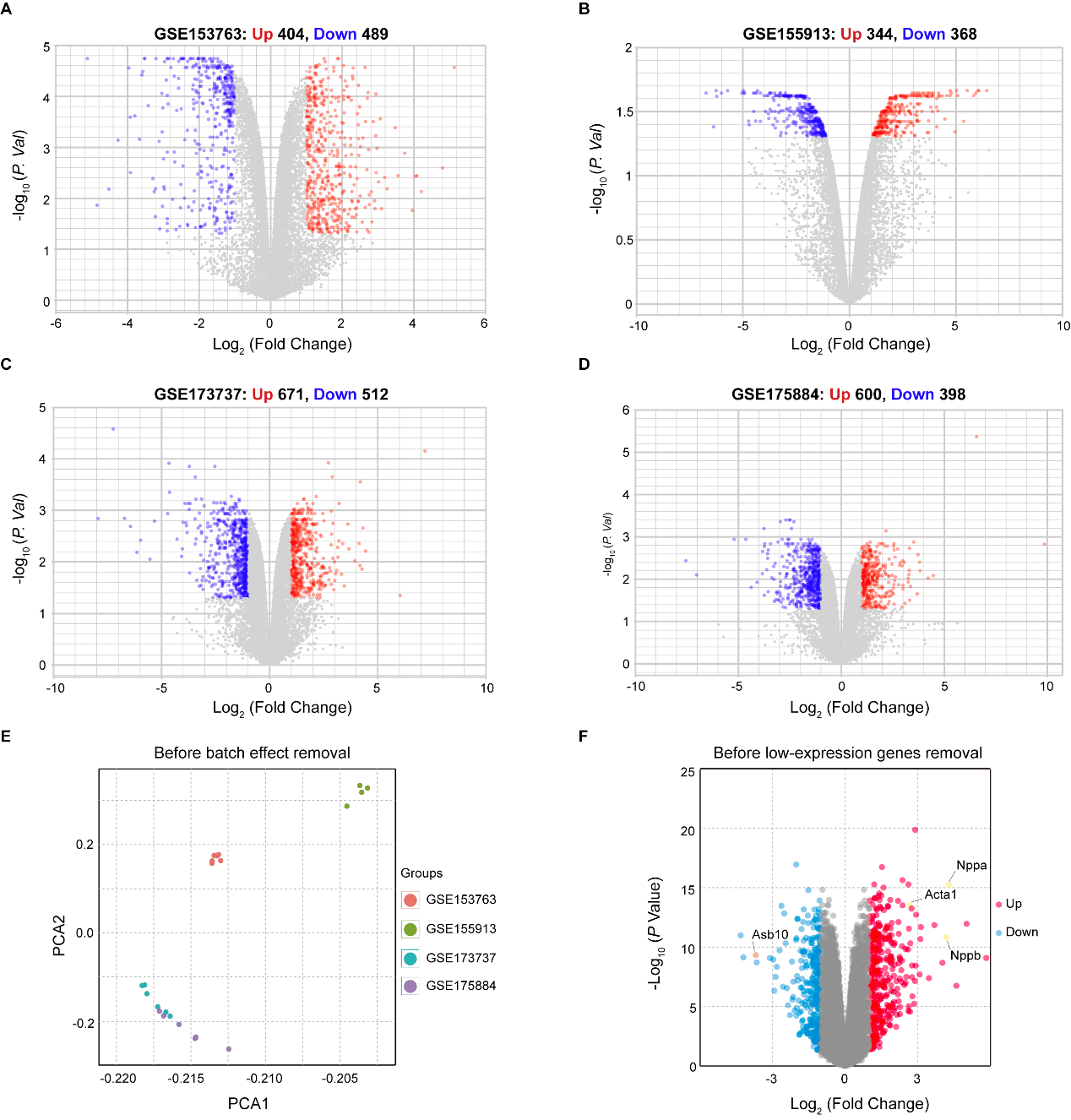


**Figure S1.** **Basic information of selected GEO datasets.** GEO datasets with identical experimental conditions were analyzed separately. Differential expression analysis was performed using the Limma package to identify differentially expressed genes (DEGs), defined by *adj. P. Val* < 0.05 and Log_2_ (Fold Change) ≥ 1. Datasets with comparable significant readouts were selected for further combination.

(A) Volcano plot of GSE153763. N=3.

(B) Volcano plot of GSE155913. N=2.

(C) Volcano plot of GSE173737. N=3.

(D) Volcano plot of GSE175884. N=3.

(E) PCA plot of merged datasets before batch effect removal.

(F) Volcano plot of merged datasets before low-expression genes removal.

Limma power differential expression analysis was conducted.


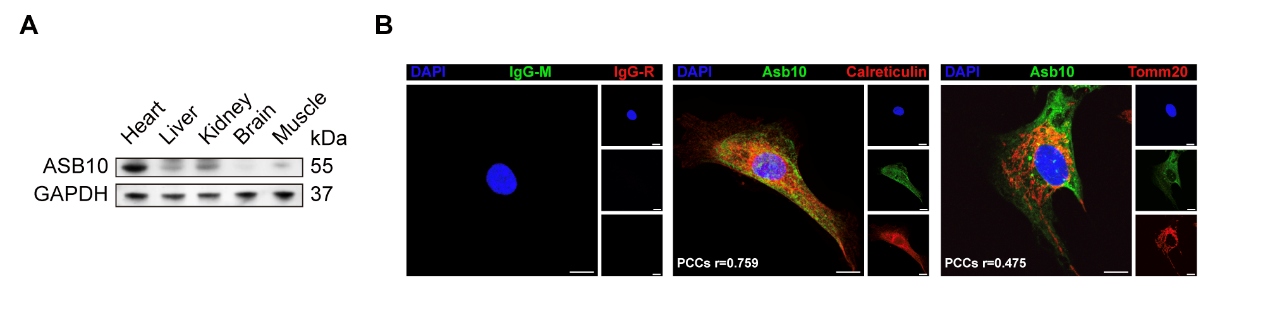


**Figure S2. Expression distribution of Asb10.**

(A) Tissue distribution of Asb10 as detected by western blot. N=1.

(B) Subcellular localization of Asb10 as determined by immunofluorescence staining of Asb10 with Calreticulin (endoplasmic reticulum marker), Tomm20 (mitochondria marker) and DAPI (nucleus). N=5.

Pearson correlation coefficients (PCCs, r) were used for the colocalization quantification. Scale bar = 10 μm.


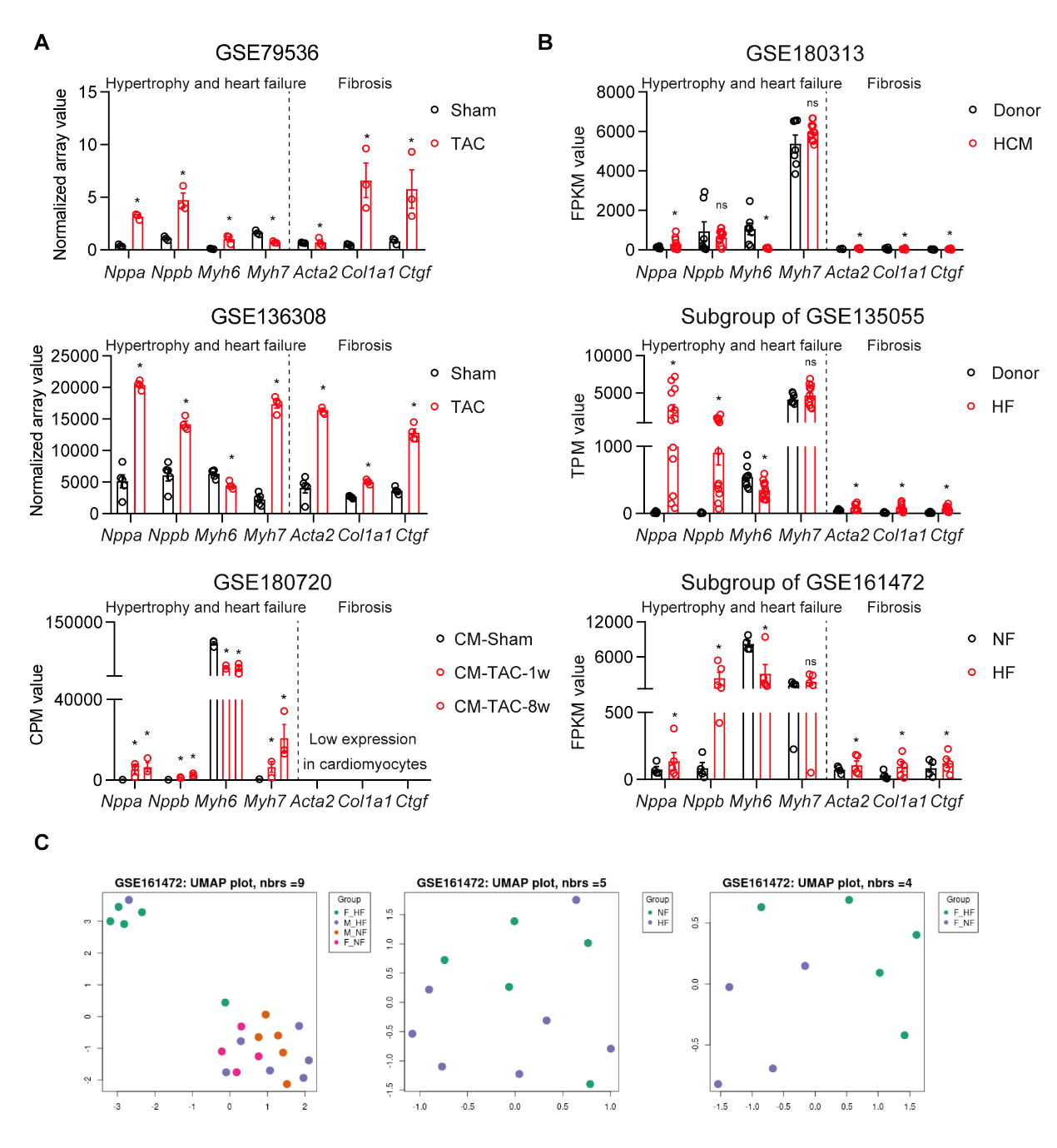


**Figure S3.** **Biomarkers in selected GEO datasets of TAC mice or patient samples.** Biomarkers of cardiac hypertrophy and heart failure (*Nppa*, *Nppb*, *Myh6* and *Myh7*), and biomarkers of cardiac fibrosis (*Acta2*, *Col1a1,* and *Ctgf*) were analyzed. GEO datasets with significant changes in these biomarkers were selected for Asb10’s expression validation.

(A) Relative expression of biomarkers from GSE79536, GSE136308 and GSE180720. N=3 for GSE79536 and GSE180720, N=5 (sham) and 4 (TAC) in GSE136308.

(B) Relative expression of biomarkers from GSE180313, GSE135055 (subgroup) and GSE161472 (subgroup). N=7 (Donor) and 13 (HCM) in GSE180313, N=9 (Donor) and 13 (HF) in GSE135055, N=4 (NF) and 5 (HF) in GSE161472.

(C) PCA plot of GSE161472: left panel shows mixed-gender samples, middle panel shows male samples, and right panel shows female samples.

Limma power differential expression analysis was conducted. Data were represented as Mean ± SEM.**adj. P. Val.*＜0.05, ^ns^*adj. P. Va*l > 0.05.


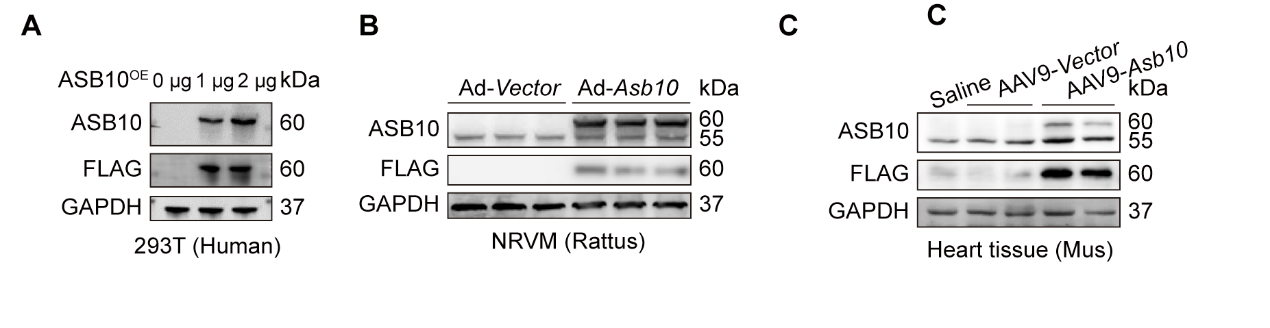


**Figure S4. Validation of Asb10 antibody.** Lysates from 293T cells transfected with ASB10-FLAG plasmid, neonatal rat ventricular myocytes (NRVMs) infected with Adenovirus-packed Asb10-FLAG and mouse heart tissues infected with AAV9-*Asb10*-FLAG were collected. Western blot analysis was performed to detect Asb10 and FLAG expressions, with GAPDH used as loading control.

1. Validation of Asb10 antibody in 293T cell lysates. N=1.
2. Validation of Asb10 antibody in NRVM lysates. N=3.
3. Validation of Asb10 antibody in mouse cardiac tissue lysates. N=1 (Saline) and 2 (*EV, Asb10*).


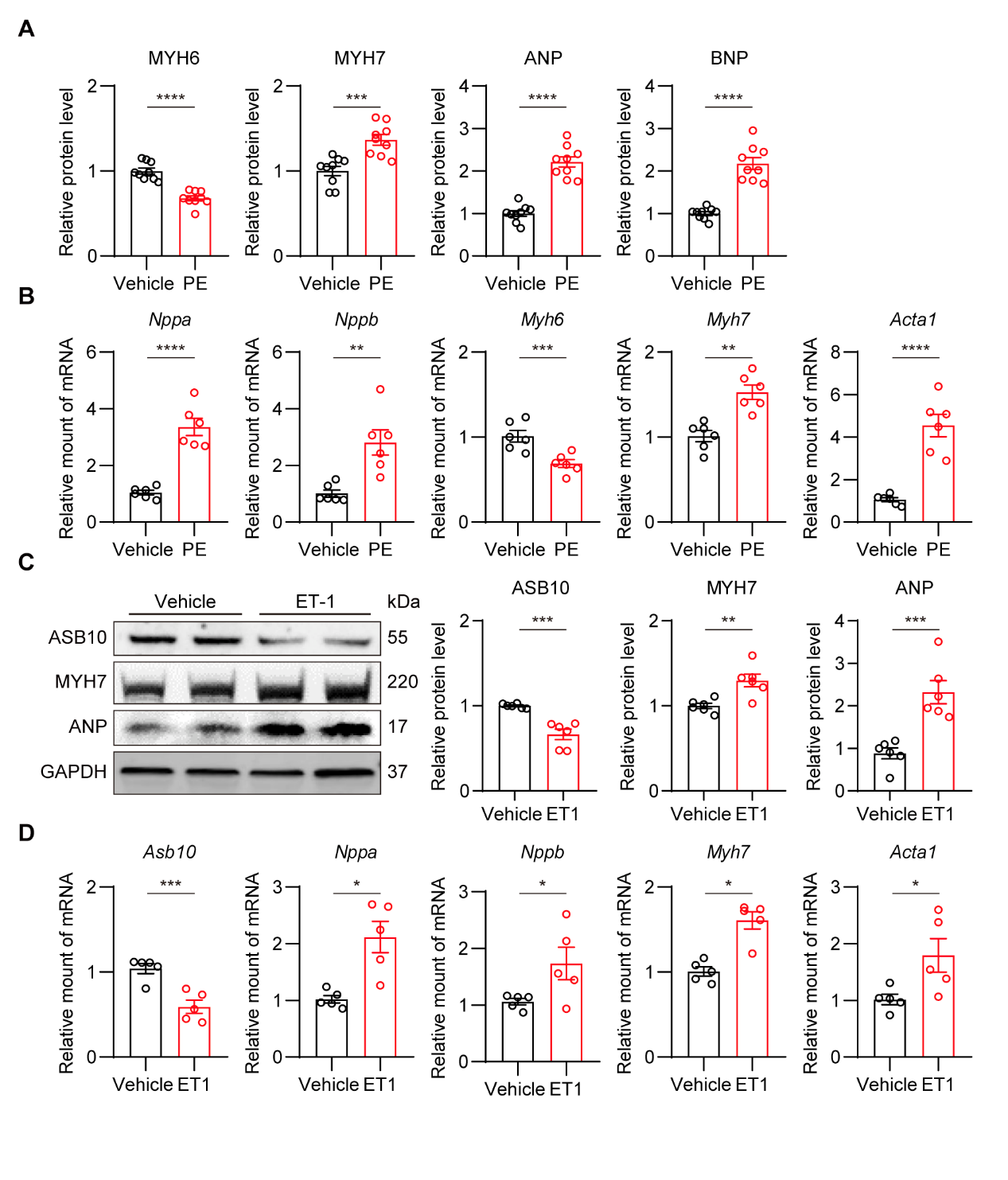


**Figure S5. Asb10 is downregulated in cardiac hypertrophy model induced by phenylephrine (PE) or endothelin-1 (ET-1).** NRVMs were treated with 50μM PE or 10 μM ET-1 for 24h, cells were then collected for western blot and qPCR detection.

(A) Quantifications of the blots in Figure 1F. N=9.

(B) Relative expressions of *Nppa,* *Nppb*, *Myh6, Myh7* and *Acta1* as detected by qPCR, related to Figure 1G. N=6.

(C) Protein expression levels of ASB10, MYH7 and ANP with GAPDH as loading control. The right panel shows the quantifications. N=6.

(D) Relative expressions of *Nppa,* *Nppb*, *Myh7* and *Acta1* as detected by qPCR. N=5.

Unpaired Student’s t-test was conducted. Data were represented as Mean ± SEM. **P* < 0.05, ***P* < 0.01, ****P* < 0.001, *****P* < 0.0001.


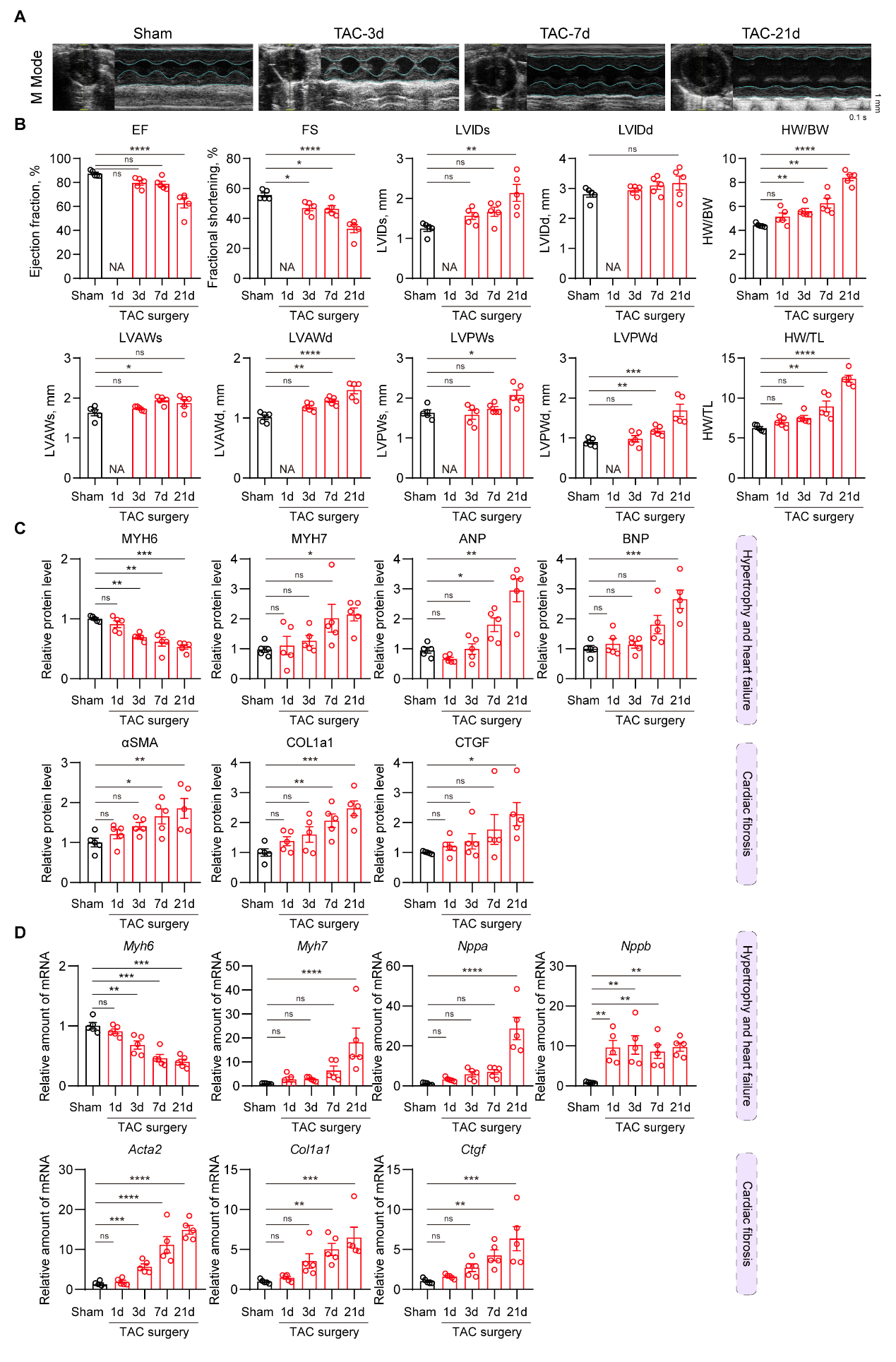


**Figure S6.** **Echocardiographic and biometric parameters of mice receiving TAC surgery at the indicated time points.** 8-week-old male wild-type mice were randomly divided into 5 groups, 1) mice received sham surgery, 2) mice received TAC surgery for 1 day, 3) mice received TAC surgery for 3 days, 4) mice received TAC surgery for 7 days, 5) mice received TAC surgery for 21 days. Cardiac function was assessed at the indicated time points, mice were then sacrificed, and the left ventricular tissues were collected for further analysis.

(A) Representative echocardiography images of mice from 5 groups. N=5.

(B) Detailed echocardiographic and biometric parameters of mice, including ejection fraction (EF), fractional shortening (FS), diameters of left ventricular internal area, anterior wall, posterior wall in systolic and diastolic phase (LVID, LVAW, LVPW), heart weight/body weight ratio (HW/BW) and heart weight/tibial length ratio (HW/TL). NA = Not available. N=5.

(C) Quantifications of the blots in Figure 1I. N=5.

(D) Relative expressions of *Myh6, Myh7*, *Nppa,* *Nppb*, *Acta2*, *Col1a1* and *Ctgf* as detected by qPCR, related to Figure 1J. N=5.

One-way ANOVA followed by Turkey’s multiple comparisons test was conducted. Data were represented as Mean ± SEM. **P* < 0.05, ***P* < 0.01, ****P* < 0.001, *****P* < 0.0001, ^ns^*P* > 0.05.


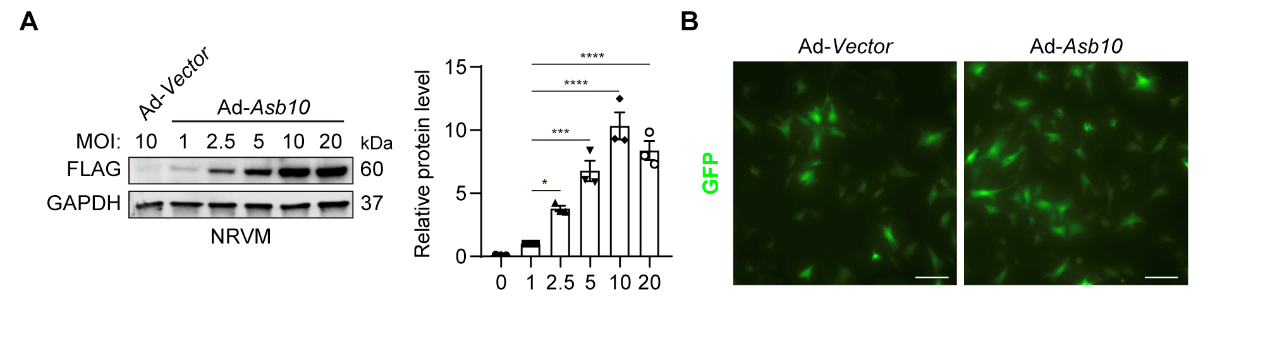


**Figure S7. Selection of adenovirus multiplicity of infection (MOI).**

(A) NRVMs were infected with different MOI of Adenovirus-*Asb10* with Adenovirus-*Vector* as control. FLAG was detected by western blot with GAPDH as loading control. The right panel shows the quantifications. N=3.

(B) NRVMs were infected with adenovirus at MOI = 10, and the successfully infected cells were GFP-positive. Representative images were shown, scale bar = 50 μm. N=3.

One-way ANOVA followed by Turkey’s multiple comparisons test was conducted. Data were represented as Mean ± SEM. **P* < 0.05, ***P* < 0.01, ****P* < 0.001, *****P* < 0.0001.


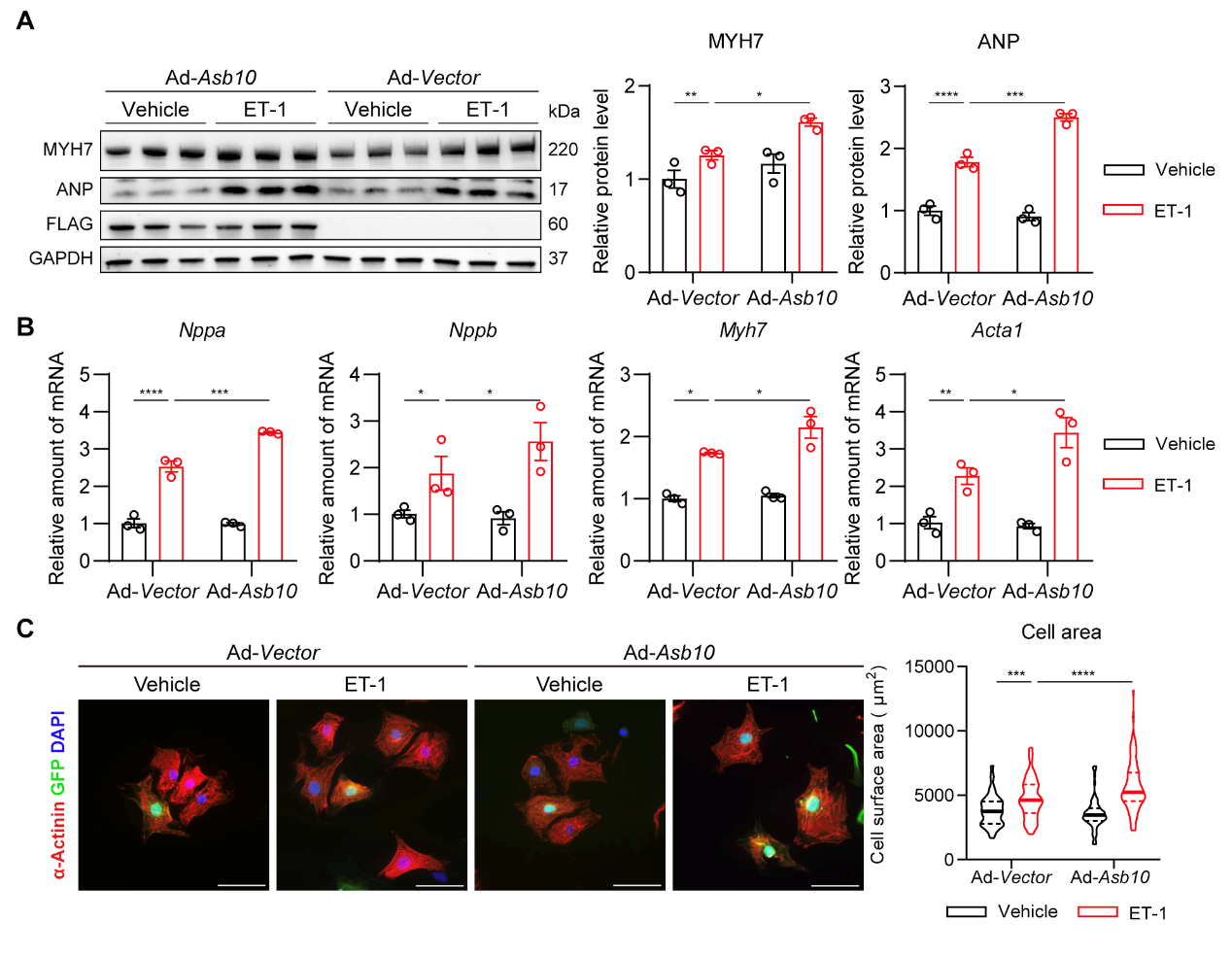


**Figure S8. Overexpression of Asb10 deteriorates ET-1-induced cardiac hypertrophy in NRVMs.** NRVMs were infected with adenovirus-packed Vector or Asb10 for 30 h, followed by 10 μM ET-1 for 24 h and were then collected for further detection.

(A) Protein expression levels of MYH7, ANP and FLAG, with GAPDH as loading control. The right panel shows the quantifications. N=3.

(B) Relative expressions of *Nppa,* *Nppb*, *Myh7* and *Acta1* as detected by qPCR. N=3.

(C) Representative images of anti-α-actinin immunofluorescence staining showing cell size. The right panel shows the quantifications. Scale bar = 30 μm. N=80 (Ad-*Vector*+Vehicle, Ad-*Vector*+ET-1), 69 (Ad-*Asb10*+Vehicle) and 85 (Ad-*Asb10*+ET-1).

Two-way ANOVA followed by Turkey’s multiple comparisons test was conducted. Data were represented as Mean ± SEM. **P* < 0.05, ***P* < 0.01, ****P* < 0.001, *****P* < 0.0001.


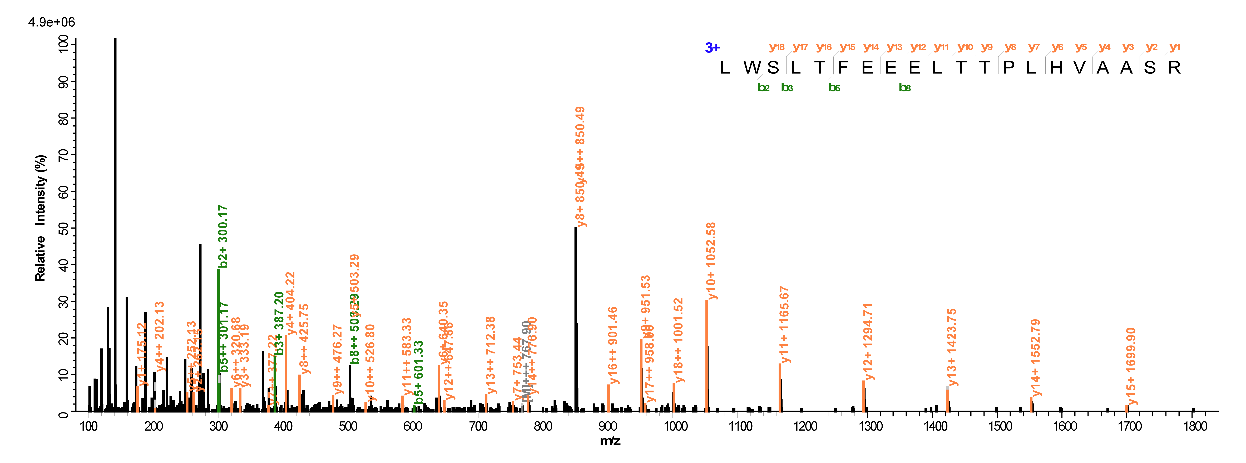


**Figure S9. Immunoprecipitation-mass spectrum analysis of Asb10 interactome in NRVMs, related to Figure 3B.**

The peptide map of the Asb10 peptide with the highest mapping score. The sequence of the Asb10 peptide is LWSLTFEEELTTPLHVAASR.


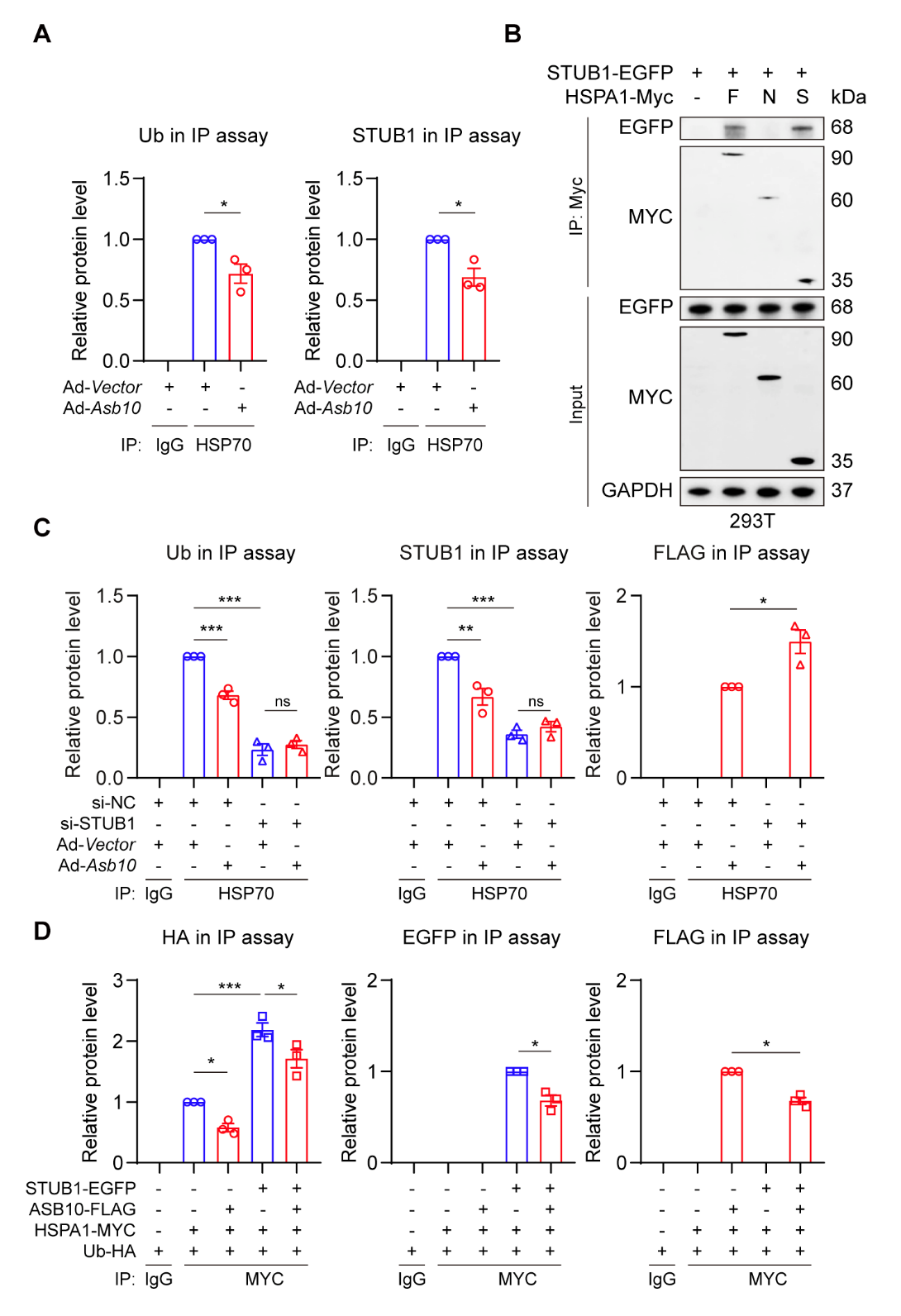


**Figure S10. Asb10 stabilizes HSP70 via competitively inhibiting STUB1-mediated ubiquitination of HSP70, related to Figure 4.**

(A) Quantifications of the blots in Figure 4G. N=3.

(B) Binding of STUB1-EGFP with truncated HSPA1-Myc as detected by co-immunoprecipitation in 293T cells. N=3.

(C) Quantifications of the blots in Figure 4H. N=3.

(D) Quantifications of the blots in Figure 4I. N=3.

Unpaired Student’s t-test (A, comparison of FLAG level in C, comparison of EGFP and FLAG levels in D) and two-way ANOVA followed by Turkey’s multiple comparisons test (comparison of Ub and STUB1 levels in C, comparison of HA level in D) were conducted. Data were represented as Mean ± SEM. **P* < 0.05, ***P* < 0.01, ****P* < 0.001, ^ns^*P* > 0.05.


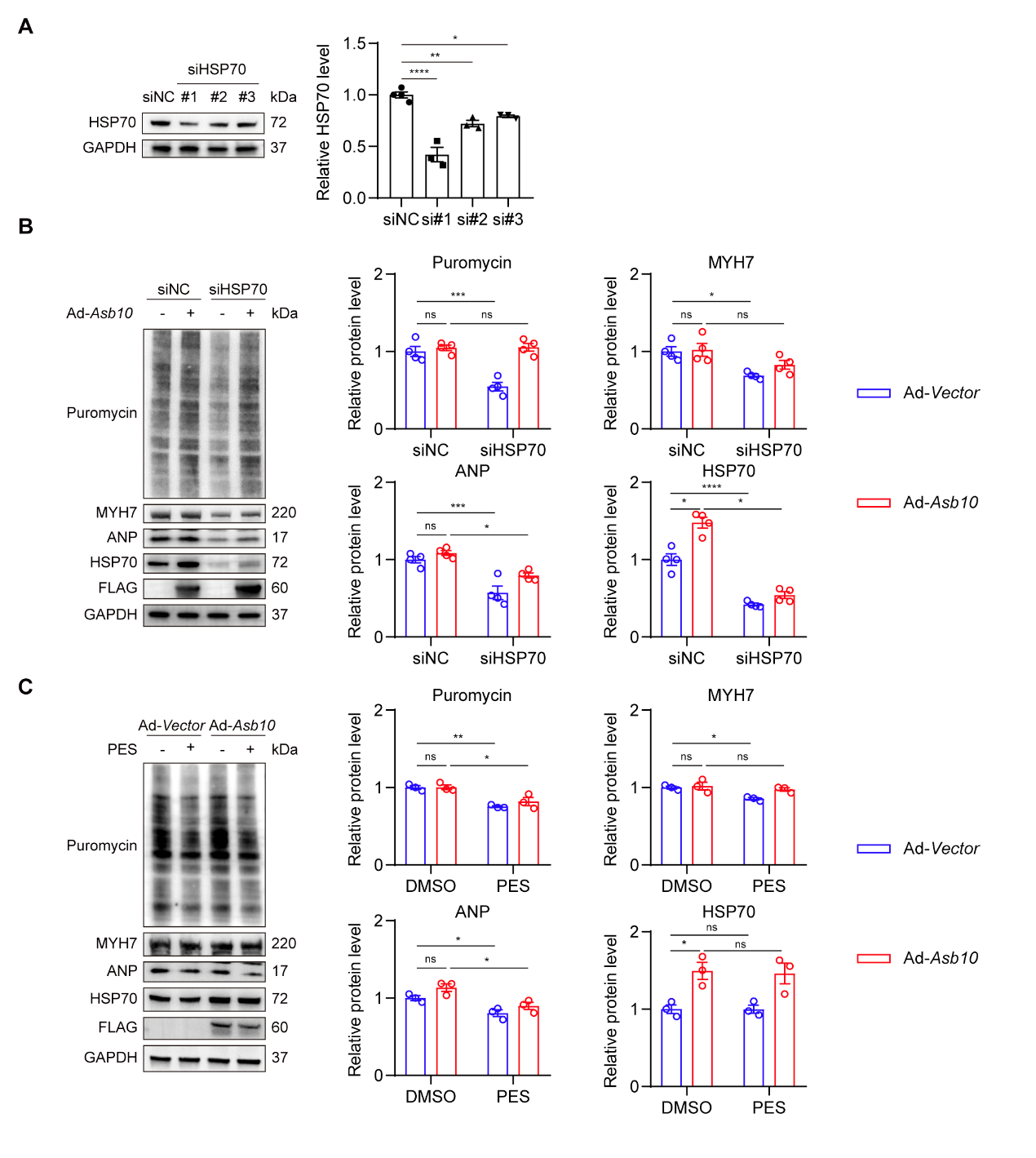


**Figure S11. HSP70 inhibition with siRNA or pharmacological inhibitor attenuates pro-hypertrophic effect of Asb10 overexpression.**

(A) Knockdown efficiency of HSP70 as determined by western blot. The right panel shows the quantifications. N=3.

(B) Knockdown of HSP70 decreases the pro-growth effect of Adenovirus-*Asb10* on NRVMs. Protein expressions of Puromycin, MYH7, ANP, HSP70 and FLAG were detected with GAPDH as loading control. The right panel shows the quantifications. N=4.

(C) Pharmacological inhibition of HSP70 by Pifithrin-μ (PES) decreased the pro-growth effect of Adenovirus-*Asb10* on NRVMs. Protein expressions of Puromycin, MYH7, ANP, HSP70 and FLAG were detected with GAPDH as loading control. N=3.

Unpaired Student’s t-test (A) and two-way ANOVA followed by Turkey’s multiple comparisons test (B) were conducted. Data were represented as Mean ± SEM. **P* < 0.05, ***P* < 0.01, ****P* < 0.001, *****P* < 0.0001, ^ns^*P* > 0.05.


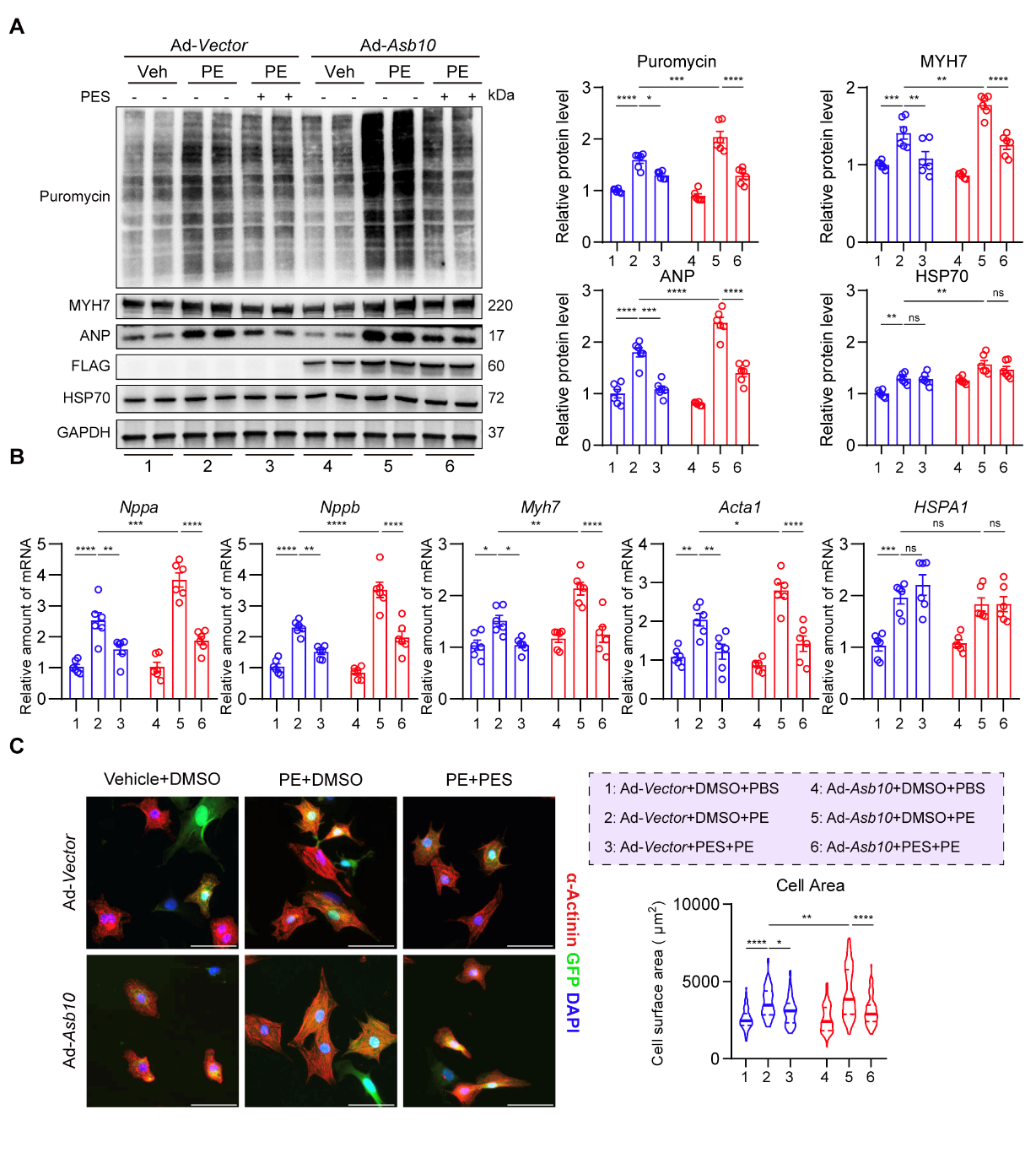


**Figure S12. Pharmacological inhibition of HSP70 partially ameliorates Asb10 overexpression-induced hypertrophic growth in NRVMs.** NRVMs were infected with adenovirus for 30 h, pretreated with 2 μM PES and were then treated with 50 μM PE for 24h. Cells were then collected for further detection.

(A) Protein expressions of Puromycin, MYH7, ANP, HSP70 and FLAG were detected with GAPDH as loading control. The right panel shows the quantifications. N=6.

(B) Relative expressions of *Nppa,* *Nppb*, *Myh7* and *Acta1* and *Hspa1* as detected by qPCR. N=6.

(C) Representative images of NRVMs stained with α-actinin and DAPI. The right panel shows the quantifications. N=81 (group 1), 74 (group 2), 71 (group 3), 74 (group 4), 83 (group 5) and 71 (group 6).

Three-way ANOVA followed by Turkey’s multiple comparisons test was conducted. Data were represented as Mean ± SEM. **P* < 0.05, ***P* < 0.01, ****P* < 0.001, *****P* < 0.0001, ^ns^*P* > 0.05.


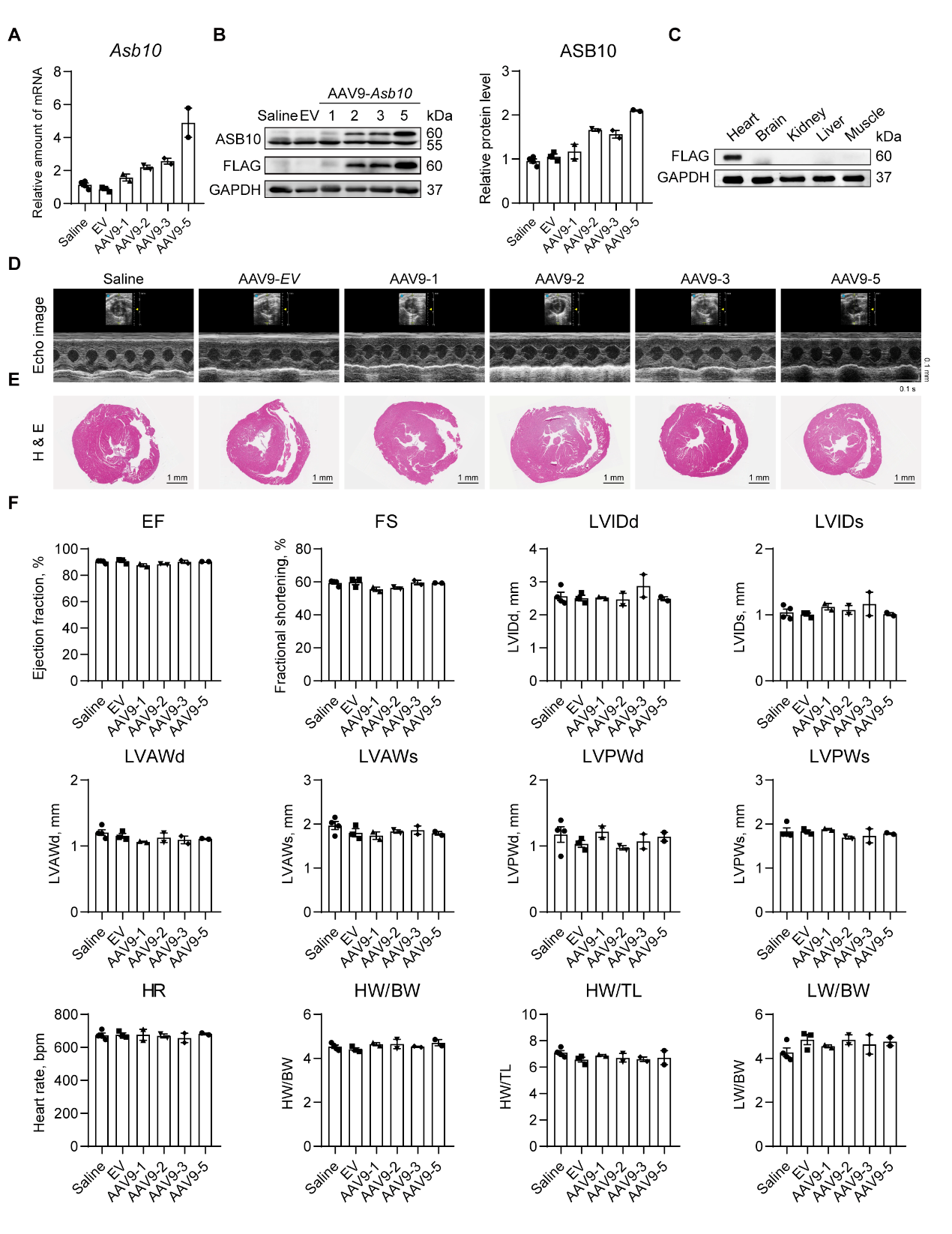


**Figure S13. Titer selection of Adeno-associated virus-packed Asb10 (AAV9-cTNT-*Asb10*).** Mice were randomly divided into 6 groups: mice injected with 1) saline, 2) empty vector (AAV9-*EV*), 3) 1.0E+11 v.g. AAV9-*Asb10* (AAV9-1), 4) 2.0E+11 v.g. AAV9-*Asb10* (AAV9-2), 5) 3.0E+11 v.g. AAV9-*Asb10* (AAV9-3), 6) 5.0E+11 v.g. AAV9-*Asb10* (AAV9-5). AAV9 infection was achieved via tail vein injection. Four weeks after injection, mice were sacrificed, and left ventricular tissues were then collected for further detection.

(A) mRNA expression of *Asb10* in left ventricular of mice from 6 groups as assessed by qPCR. N=4 (Saline and EV), and 2 (AAV9-1, 2, 3, 5).

(B) Protein expression efficiency of Asb10 in left ventricular of mice from 6 groups as determined by western blot. The right panel shows the quantifications. N=4 (Saline and EV), and 2 (AAV9-1, 2, 3, 5).

(C) Tissue specificity of Asb10 overexpression as determined by western blot. N=1.

(D) Representative echocardiography images of mice from 6 groups. N=4 (Saline and EV), and 2 (AAV9-1, 2, 3, 5).

(E) Representative histology images of mice from 6 groups. N=4 (Saline and EV), and 2 (AAV9-1, 2, 3, 5).

(F) Asb10 overexpression with different doses of AAV9-*Asb*10 does not influence echocardiographic and biometric parameters of mice, including EF, FS, diameters of LVID, LVAW, LVPW in systolic and diastolic phase and HW/BW, HW/TL, LW/BW. N=4 (Saline and EV), and 2 (AAV9-1, 2, 3, 5).

Data were represented as Mean ± SEM.


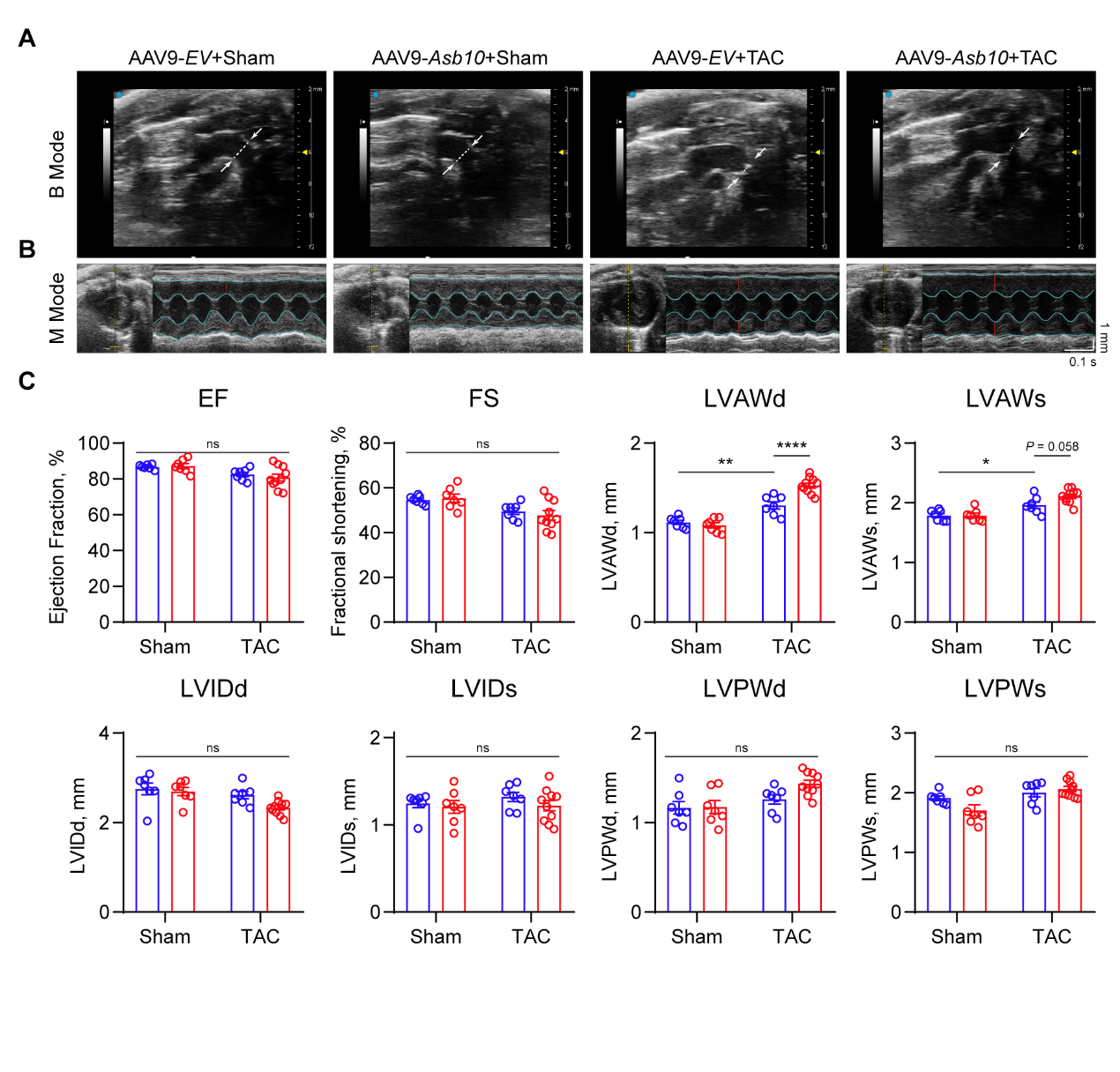


**Figure S14. Overexpression of Asb10 increased left ventricular anterior wall thickness of mice at the early postoperative time point following TAC surgery.** Mice were randomly divided into 4 groups: mice injected with 1) empty vector and received sham surgery (AAV9-*EV*+Sham), 2) AAV9-*Asb10* and received sham surgery (AAV9-*Asb10*+Sham), 3) empty vector and received TAC surgery (AAV9-*EV* +TAC), 4) AAV9-*Asb10* and received TAC surgery (AAV9-*Asb10*+TAC). AAV9 infection was achieved via tail vein injection. Four weeks after injection, mice received either TAC or sham surgery. The cardiac function of mice was assessed using a non-invasive high-resolution imaging system.

(A) Representative B mode images of aortic arch from the 4 groups (correlated with Fig 6A). N=7 (*EV*+Sham, *Asb10*+Sham, *EV*+TAC) and 10 (*Asb10*+TAC).

(B) Representative M mode images of echocardiography from the 4 groups. N=7 (*EV*+Sham, *Asb10*+Sham, *EV*+TAC) and 10 (*Asb10*+TAC).

(C) Detailed echocardiographic parameters of mice at the early postoperative time point following TAC surgery, including EF, FS, and diameters of LVID, LVAW, LVPW in the systolic and diastolic phases. N=7 (*EV*+Sham, *Asb10*+Sham, *EV*+TAC) and 10 (*Asb10*+TAC).

Two-way ANOVA followed by Turkey’s multiple comparisons test was conducted. Data were represented as Mean ± SEM. **P* < 0.05, ***P* < 0.01, *****P* < 0.0001, ^ns^*P* > 0.05.


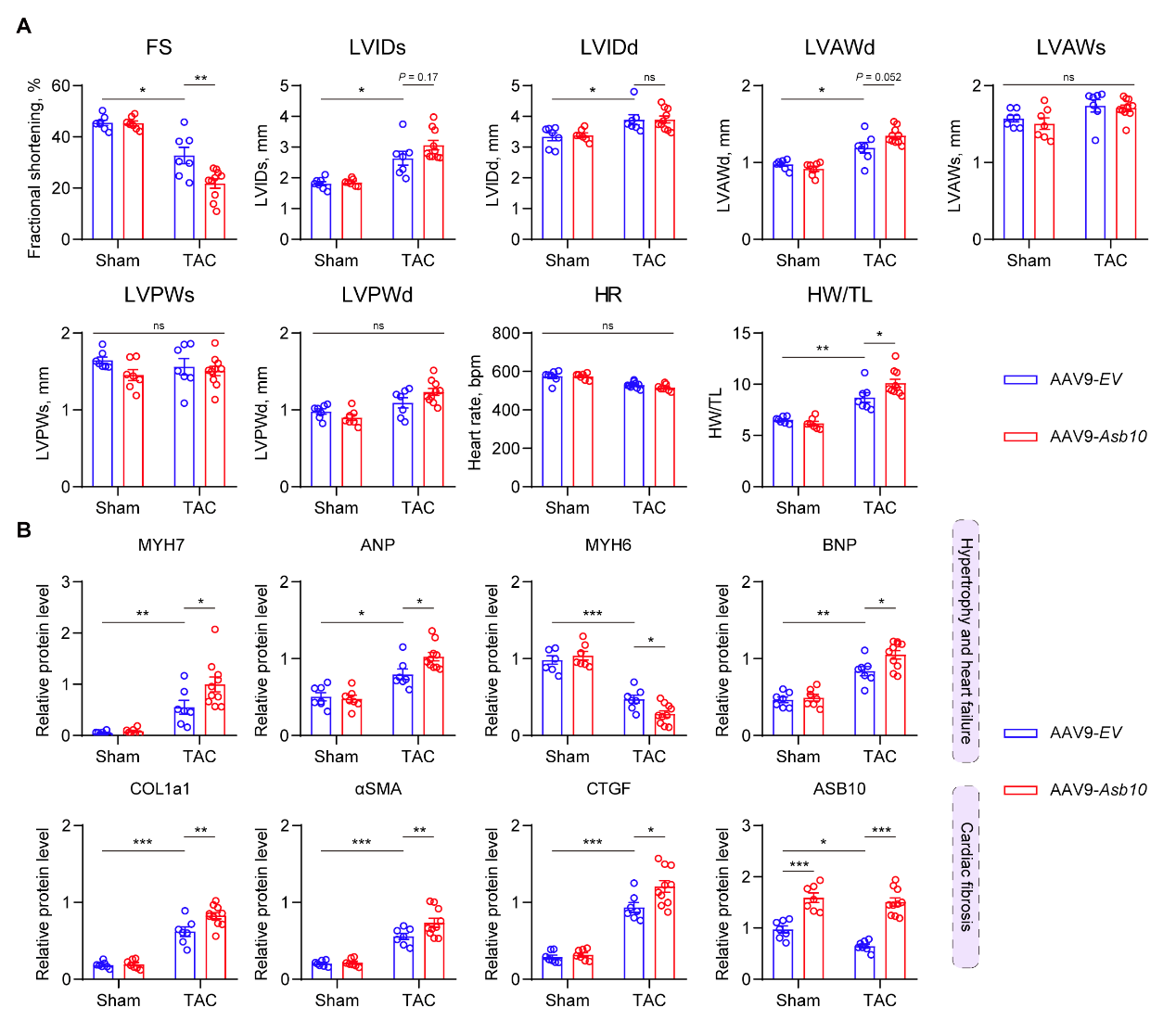


**Figure S15. Overexpression of Asb10 deteriorates cardiac function and exacerbates cardiac hypertrophy as well as fibrosis after prolonged TAC surgery.** After prolonged TAC surgery, the cardiac systolic function was assessed using a non-invasive high-resolution imaging system. Mice were then sacrificed, and left ventricular tissues were collected for further detection.

(A) Detailed echocardiographic and biometric parameters of mice after prolonged TAC surgery, including FS, diameters of LVID, LVAW, LVPW in the systolic and diastolic phases, and HW/TL. N=7 (*EV*+Sham, *Asb10*+Sham, *EV*+TAC) and 10 (*Asb10*+TAC).

(B) Quantifications of the blots in Figure 6H. N=7 (*EV*+Sham, *Asb10*+Sham, *EV*+TAC) and 10 (*Asb10*+TAC).

Two-way ANOVA followed by Turkey’s multiple comparisons test was conducted. Data were represented as Mean ± SEM. **P* < 0.05, ***P* < 0.01, ****P* < 0.001, ^ns^*P* > 0.05.


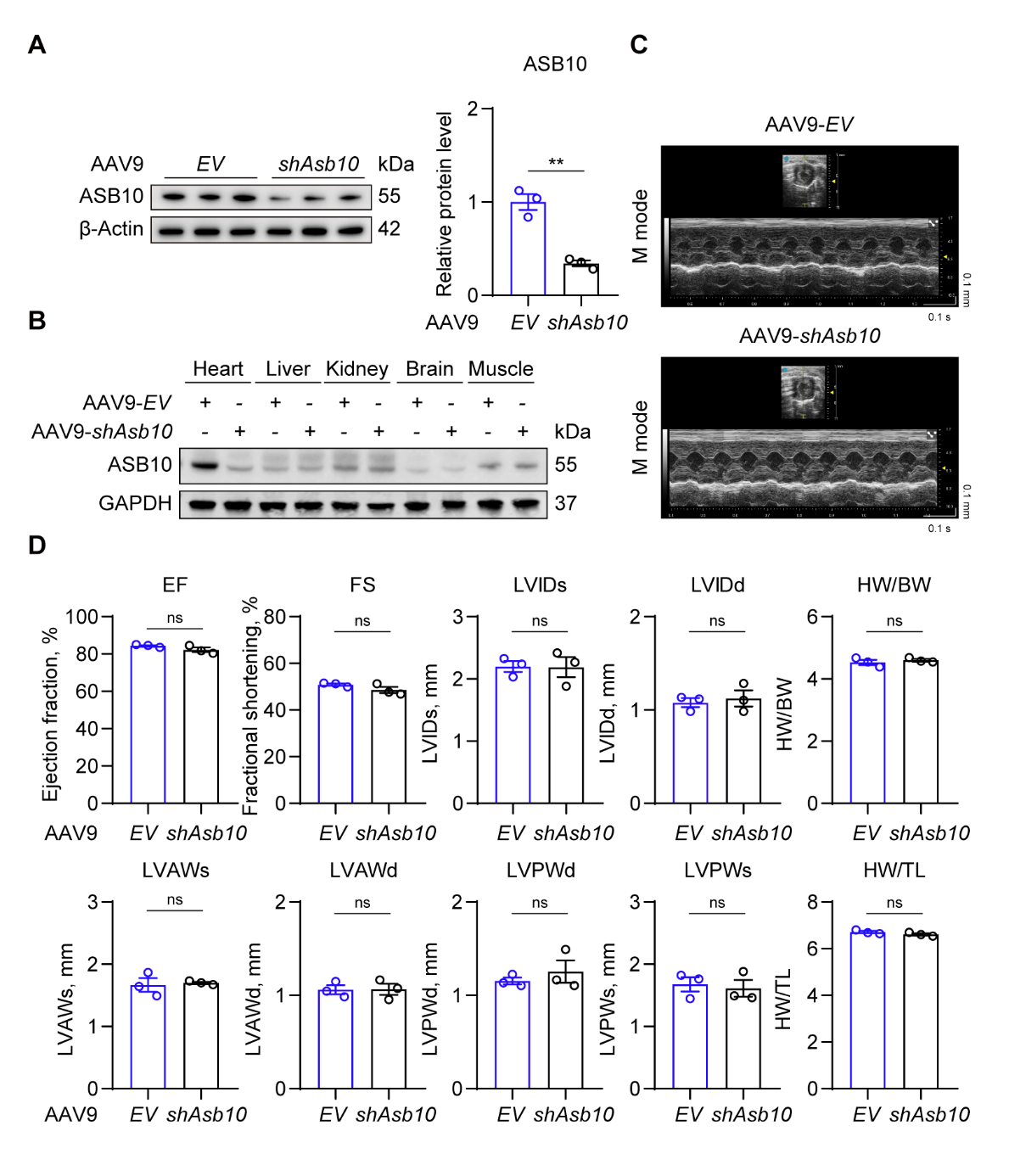


**Figure S16. Injection of AAV9-cTNT-*shAsb10* specifically decreases the expression of Asb10 in heart tissue and has no effect on basal cardiac function.**

Mice were randomly divided into 2 groups: mice injected with 1) empty vector (AAV9-*EV*), 2) 5.0E+11 v.g. AAV9-*shAsb10* (AAV9-*shAsb10*). AAV9 infection was achieved via tail vein injection. Four weeks after injection, mice were sacrificed, and left ventricular tissues were then collected for further detection.

(A) Asb10 knockdown efficiency as determined by western blot. The right panel shows the quantifications. N=3.

(B) Asb10 knockdown specificity as determined by western blot. N=1.

(C) Representative echocardiography images of mice from the 2 groups. N=3.

(D) Knockdown of Asb10 did not influence basal echocardiographic parameters of mice, including EF, FS, diameters of LVID, LVAW, LVPW in systolic and diastolic phase, and HW/BW as well as HW/TL. N=3.

Unpaired Student’s t-test was conducted. Data were represented as Mean ± SEM. ^ns^*P* > 0.05, ***P* < 0.01.

**
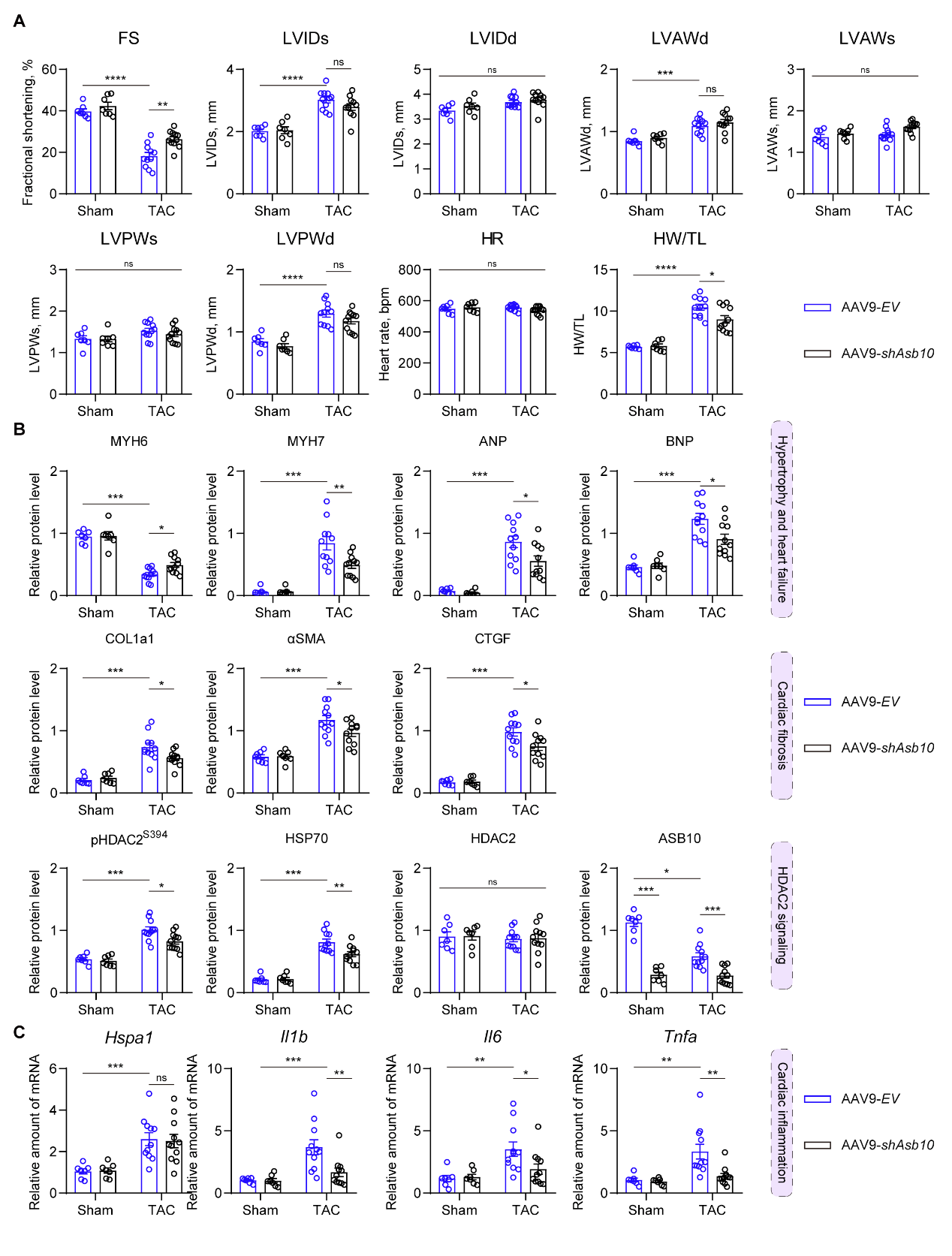
**

**Figure S17. Knockdown of Asb10 ameliorates cardiac hypertrophy, fibrosis and inflammation, related to Figure 8.**

Mice were randomly divided into 4 groups: mice injected with 1) empty vector and received sham surgery (AAV9-*EV*+Sham), 2) AAV9-*shAsb10* and received sham surgery (AAV9-*shAsb10*+Sham), 3) empty vector and received TAC surgery (AAV9-*EV* +TAC), 4) AAV9-*Asb10* and received TAC surgery (AAV9-*shAsb10*+TAC). AAV9 infection was achieved via tail vein injection. Four weeks after injection, mice received either TAC or sham surgery. The cardiac function of mice was assessed using a non-invasive high-resolution imaging system. Then, mice were sacrificed, and left ventricular tissues were then collected for further detection.

(A) Detailed echocardiographic and biometric parameters of mice after TAC surgery, including FS, diameters of LVID, LVAW, LVPW in the systolic and diastolic phases, and HW/TL. N=7 (*EV*+Sham, *shAsb10*+Sham) and 11 (*EV*+TAC, *shAsb10*+TAC).

(B) Quantifications of the blots in Figure 8H. N=7 (*EV*+Sham, *shAsb10*+Sham) and 11 (*EV*+TAC, *shAsb10*+TAC).

(C) Relative mRNA expressions of *Hspa1*, *Il1b*, *Il6* and *Tnfa* as detected by qPCR. N=7 (*EV*+Sham, *shAsb10*+Sham) and 11 (*EV*+TAC, *shAsb10*+TAC).

Two-way ANOVA followed by Turkey’s multiple comparisons test was conducted. Data were represented as Mean ± SEM. **P* < 0.05, ***P* < 0.01, ****P* < 0.001, *****P* < 0.0001, ^ns^*P* > 0.05.
