# Supplementary material for: Asb10 accelerates pathological cardiac remodeling by stabilizing HSP70
Source: Cell Death Dis. 2025 May 22;16(1):409. doi: 10.1038/s41419-025-07735-5 (PMC12095639; doi:10.1038/s41419-025-07735-5)

Figure 1F

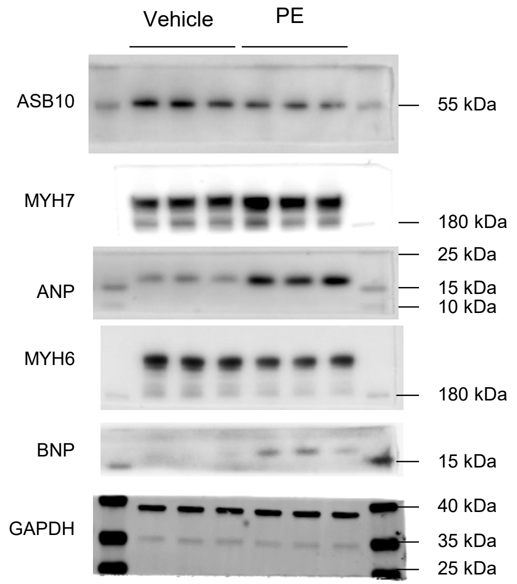

Figure 1I

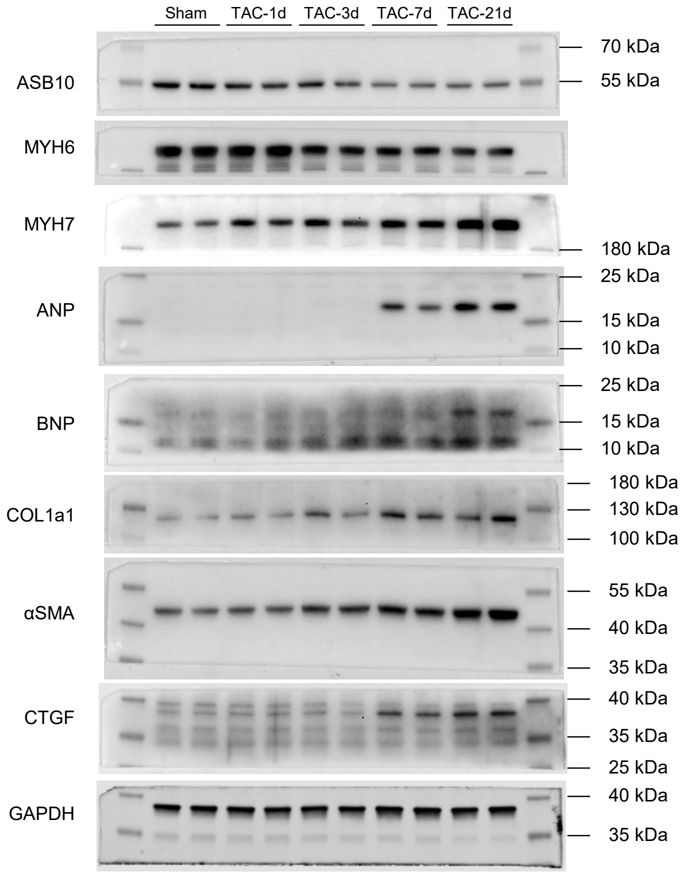

Figure 2A

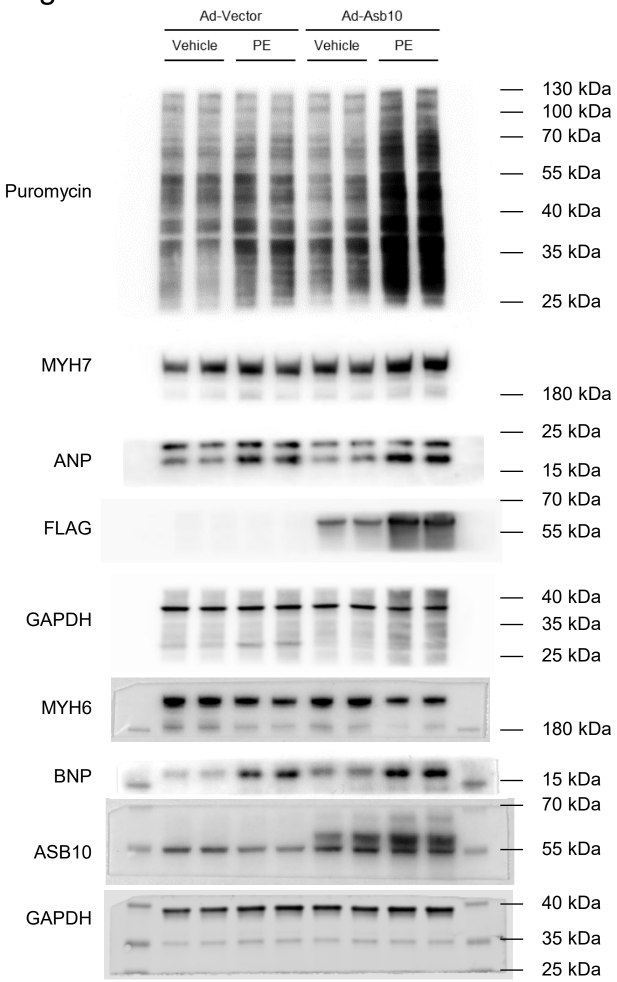

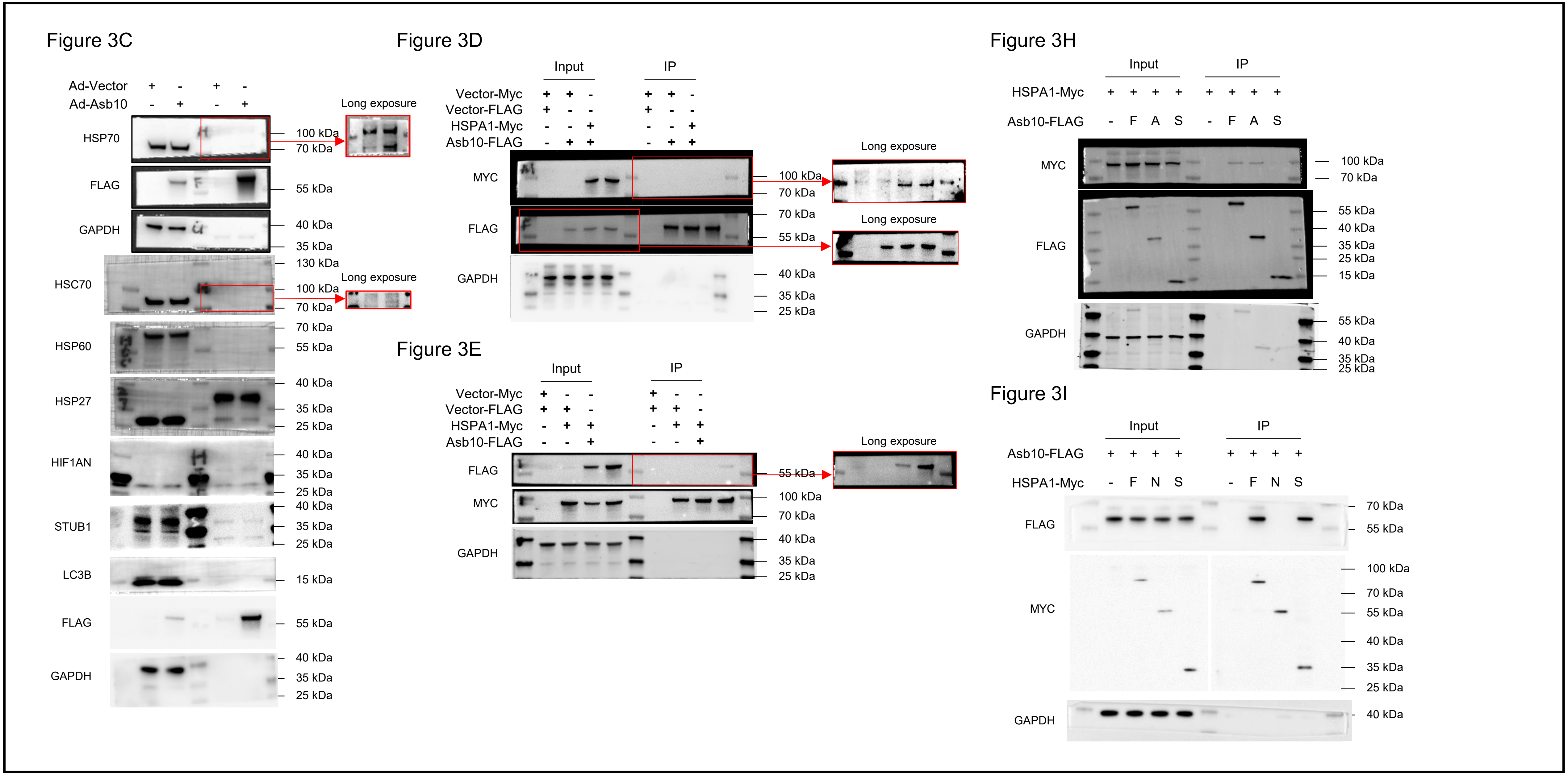

Figure 3C
Ad-Vector + - + -
Ad-Asb10 - + - +
Long exposure
HSP70
100 kDa
70 kDa
FLAG
55 kDa
GAPDH
40 kDa
35 kDa
130 kDa
HSC70
100 kDa
Long exposure
70 kDa
HSP60
70 kDa
55 kDa
HSP27
40 kDa
35 kDa
25 kDa
HIF1AN
40 kDa
35 kDa
25 kDa
STUB1
40 kDa
35 kDa
25 kDa
LC3B
15 kDa
FLAG
55 kDa
GAPDH
40 kDa
35 kDa
25 kDa
Figure 3D
Input
IP
Vector-Myc + + - + + -
Vector-FLAG + - - + - -
HSPA1-Myc - - + - - +
Asb10-FLAG - + + - + +
Long exposure
MYC
100 kDa
70 kDa
FLAG
70 kDa
Long exposure
55 kDa
GAPDH
40 kDa
35 kDa
25 kDa
Figure 3E
Input
IP
Vector-Myc + - - + - -
Vector-FLAG + + - + + -
HSPA1-Myc - + + - + +
Asb10-FLAG - - + - - +
Long exposure
FLAG
55 kDa
MYC
100 kDa
70 kDa
GAPDH
40 kDa
35 kDa
25 kDa
Figure 3H
Input
IP
HSPA1-Myc + + + + + + + +
Asb10-FLAG - F A S - F A S
MYC
100 kDa
70 kDa
FLAG
55 kDa
40 kDa
35 kDa
25 kDa
15 kDa
GAPDH
55 kDa
40 kDa
35 kDa
25 kDa
Figure 3I
Input
IP
Asb10-FLAG + + + + + + + +
HSPA1-Myc - F N S - F N S
FLAG
70 kDa
55 kDa
MYC
100 kDa
70 kDa
55 kDa
40 kDa
35 kDa
25 kDa
GAPDH
40 kDa

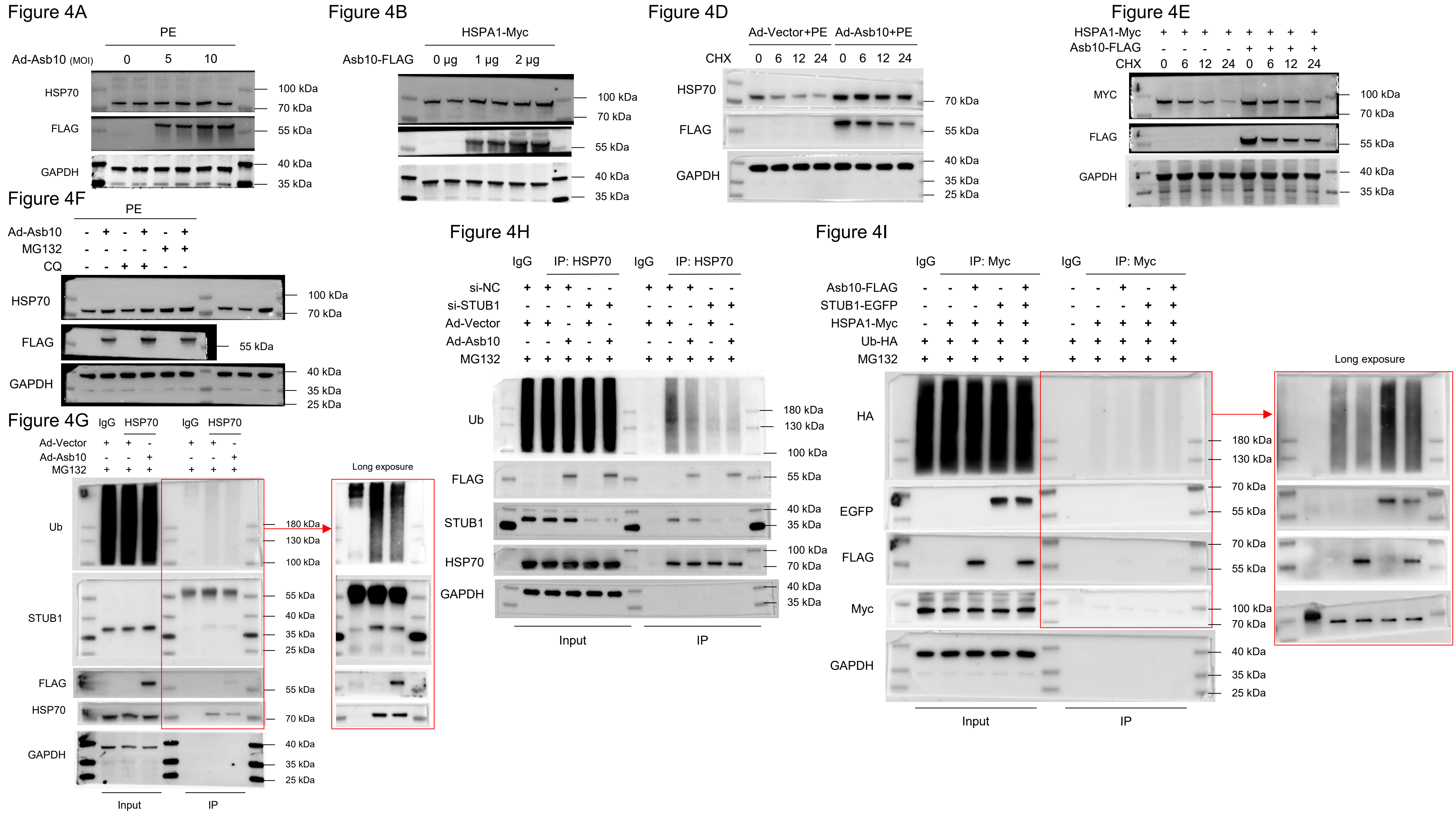

Figure 4A
PE
Ad-Asb10 (MOI) 0 5 10
HSP70
FLAG
GAPDH
100 kDa
70 kDa
55 kDa
40 kDa
35 kDa
Figure 4B
HSPA1-Myc
Asb10-FLAG 0 μg 1 μg 2 μg
100 kDa
70 kDa
55 kDa
40 kDa
35 kDa
Figure 4D
Ad-Vector+PE Ad-Asb10+PE
CHX 0 6 12 24 0 6 12 24
HSP70
FLAG
GAPDH
70 kDa
55 kDa
40 kDa
35 kDa
25 kDa
Figure 4E
HSPA1-Myc + + + + + + + +
Asb10-FLAG - - - - + + + +
CHX 0 6 12 24 0 6 12 24
MYC
FLAG
GAPDH
100 kDa
70 kDa
55 kDa
40 kDa
35 kDa
Figure 4F
PE
Ad-Asb10 - + - + - +
MG132 - - - - + +
CQ - - + + - -
HSP70
FLAG
GAPDH
100 kDa
70 kDa
55 kDa
40 kDa
35 kDa
25 kDa
Figure 4G
IgG HSP70 IgG HSP70
Ad-Vector + + - + + -
Ad-Asb10 - - + - - +
MG132 + + + + + +
Long exposure
Ub
STUB1
FLAG
HSP70
GAPDH
180 kDa
130 kDa
100 kDa
55 kDa
40 kDa
35 kDa
25 kDa
55 kDa
70 kDa
40 kDa
35 kDa
25 kDa
Input IP
Figure 4H
IgG IP: HSP70 IgG IP: HSP70
si-NC + + + - - + + + - -
si-STUB1 - - - + + - - - + +
Ad-Vector + + - + - + + - + -
Ad-Asb10 - - + - + - - + - +
MG132 + + + + + + + + + +
Ub
FLAG
STUB1
HSP70
GAPDH
180 kDa
130 kDa
100 kDa
55 kDa
40 kDa
35 kDa
100 kDa
70 kDa
40 kDa
35 kDa
Input IP
Figure 4I
IgG IP: Myc IgG IP: Myc
Asb10-FLAG - - + - + - - + - +
STUB1-EGFP - - - + + - - - + +
HSPA1-Myc - + + + + - + + + +
Ub-HA + + + + + + + + + +
MG132 + + + + + + + + + +
Long exposure
HA
EGFP
FLAG
Myc
GAPDH
180 kDa
130 kDa
70 kDa
55 kDa
70 kDa
55 kDa
100 kDa
70 kDa
40 kDa
35 kDa
25 kDa
Input IP

Figure 5A

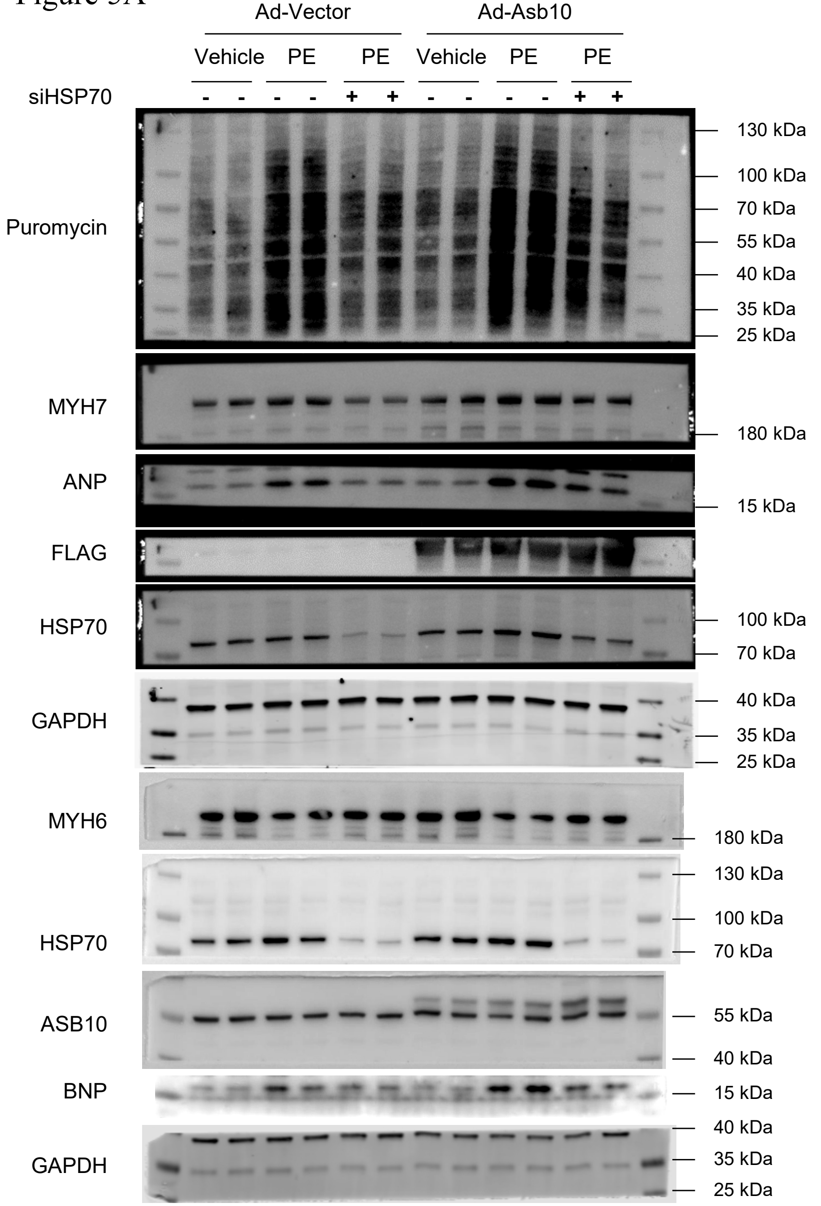

Figure 6H

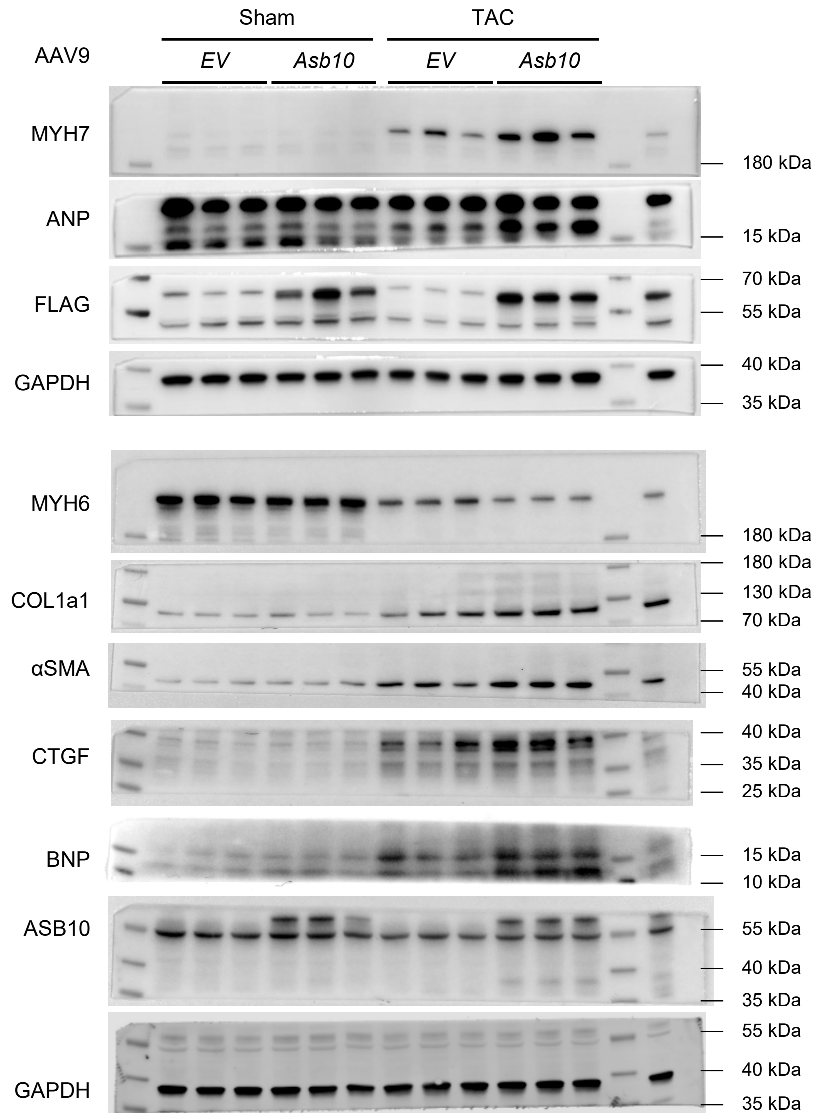

Figure 7E

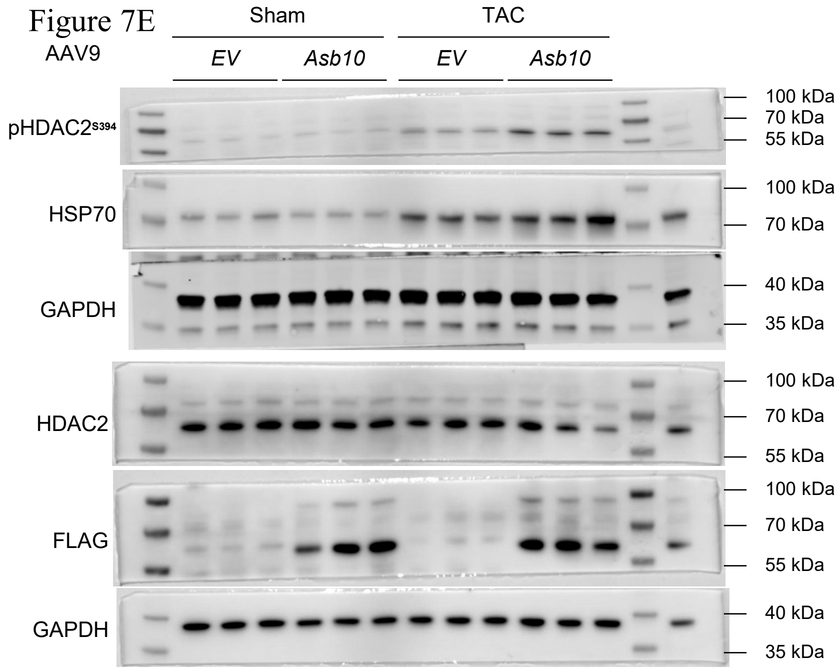

Figure 7F

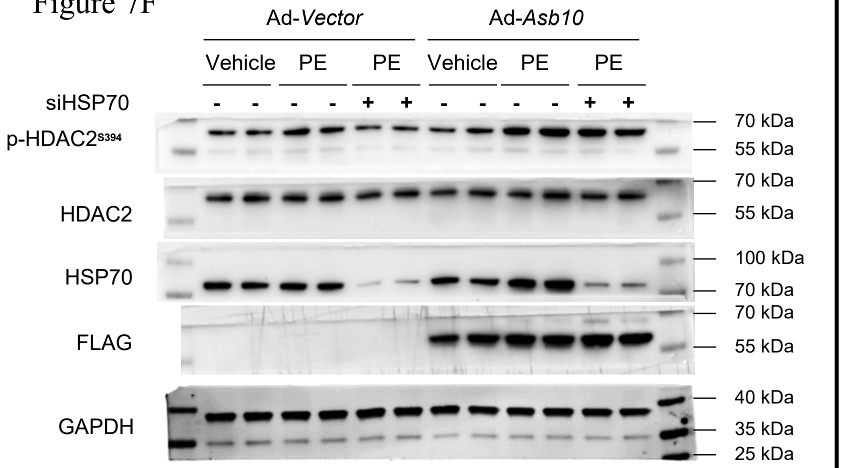

Figure 8H

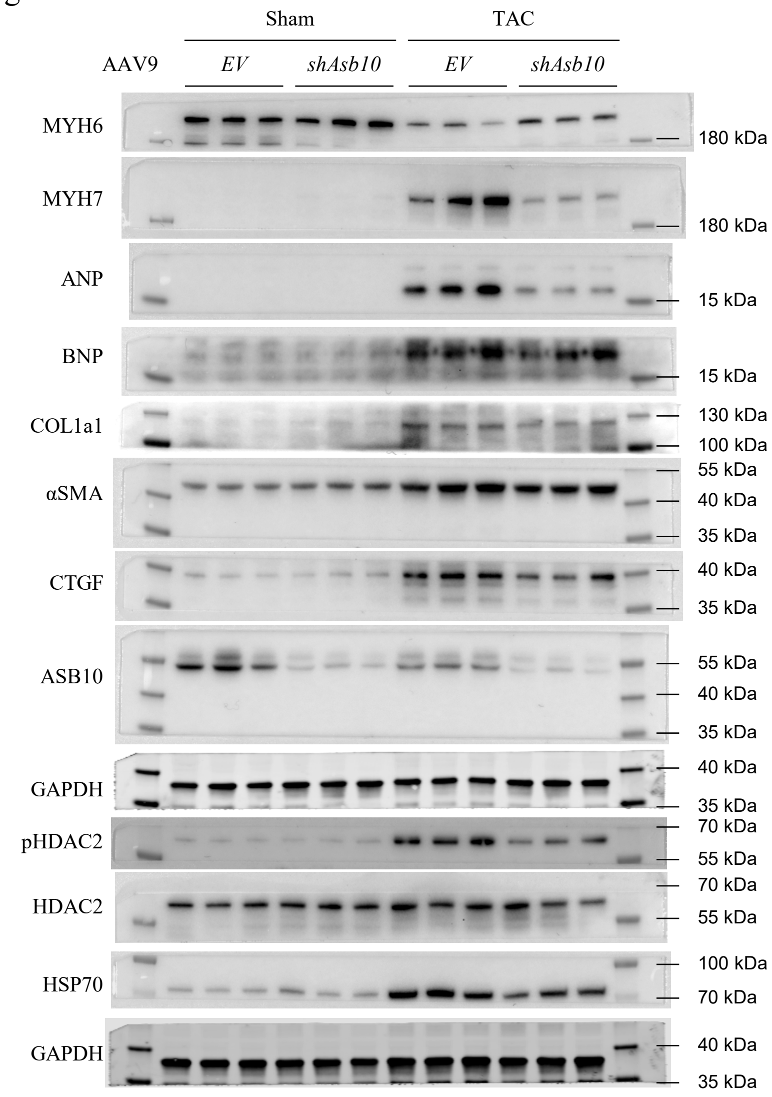

Sham
TAC
AAV9
EV
shAsb10
EV
shAsb10
MYH6
180 kDa
MYH7
180 kDa
ANP
15 kDa
BNP
15 kDa
COL1a1
130 kDa
100 kDa
αSMA
55 kDa
40 kDa
35 kDa
CTGF
40 kDa
35 kDa
ASB10
55 kDa
40 kDa
35 kDa
GAPDH
40 kDa
35 kDa
pHDAC2
70 kDa
55 kDa
HDAC2
70 kDa
55 kDa
HSP70
100 kDa
70 kDa
GAPDH
40 kDa
35 kDa

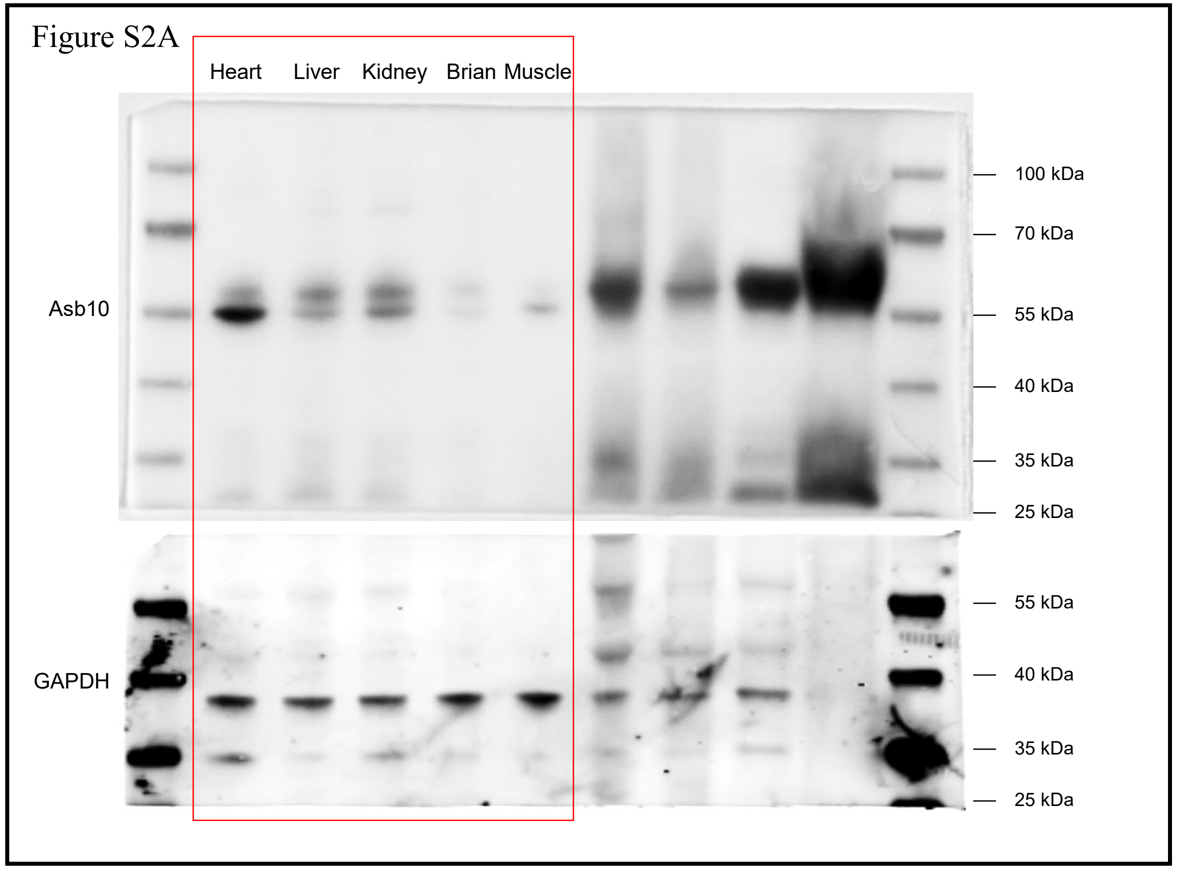
Figure S2A
Heart
Liver
Kidney
Brian
Muscle
Asb10
GAPDH
100 kDa
70 kDa
55 kDa
40 kDa
35 kDa
25 kDa
55 kDa
40 kDa
35 kDa
25 kDa

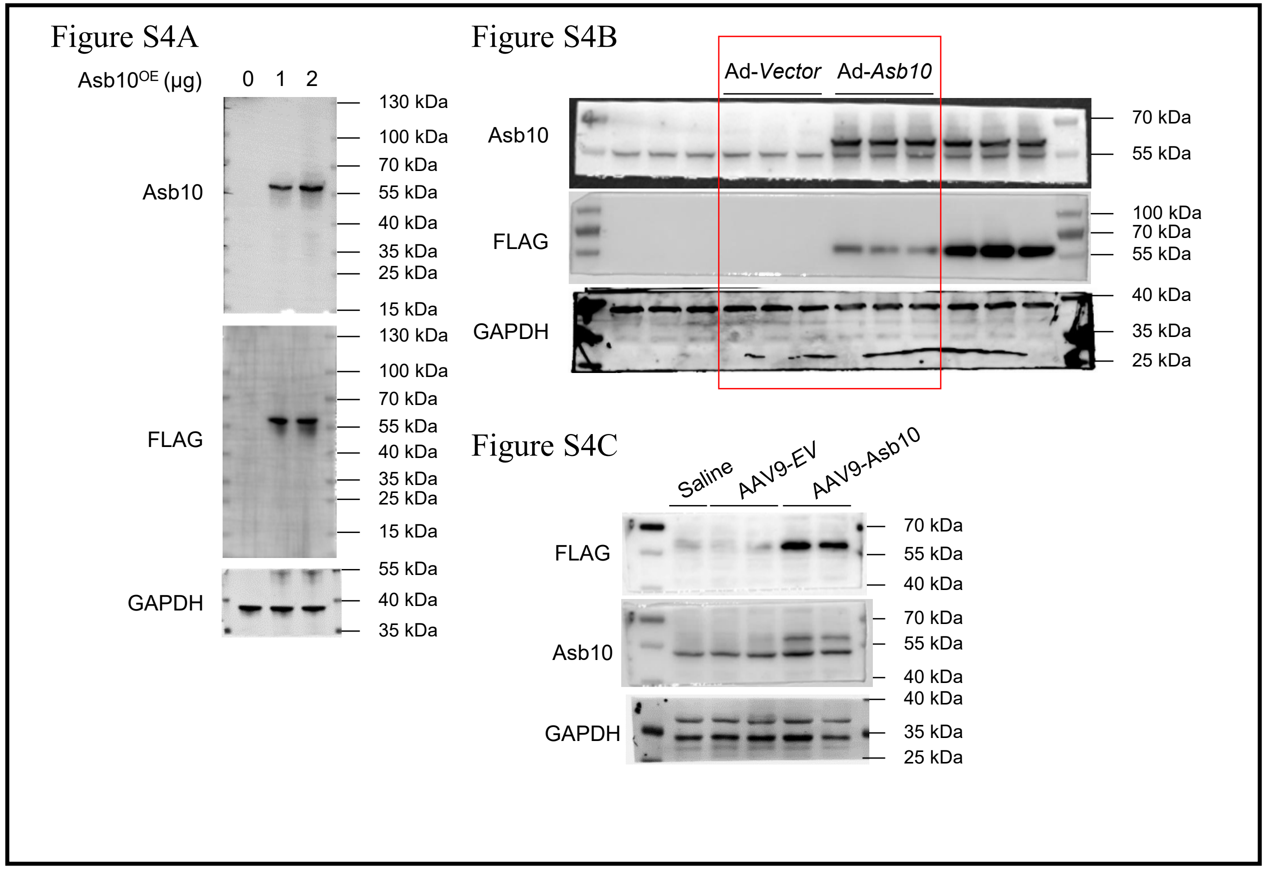
Figure S4A
Asb10OE (μg)
0
1
2
Asb10
FLAG
GAPDH
130 kDa
100 kDa
70 kDa
55 kDa
40 kDa
35 kDa
25 kDa
15 kDa
Figure S4B
Ad-Vector
Ad-Asb10
Asb10
FLAG
GAPDH
70 kDa
55 kDa
100 kDa
40 kDa
35 kDa
25 kDa
Figure S4C
Saline
AAV9-EV
AAV9-Asb10

Figure S5C

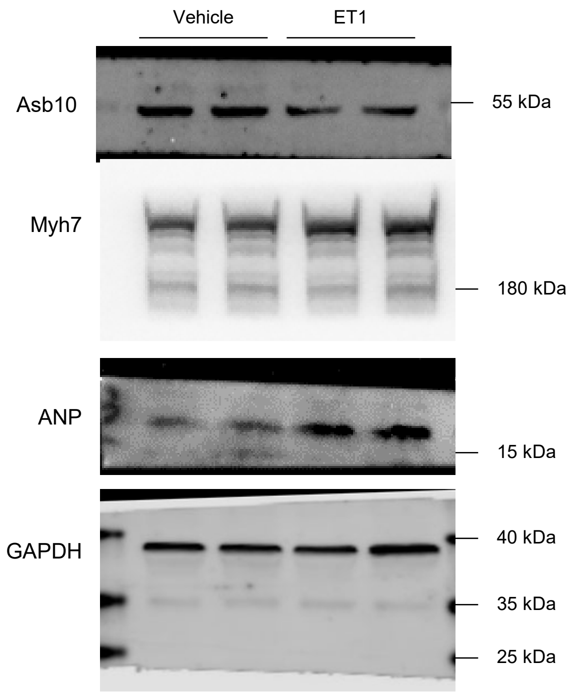

Figure S7A

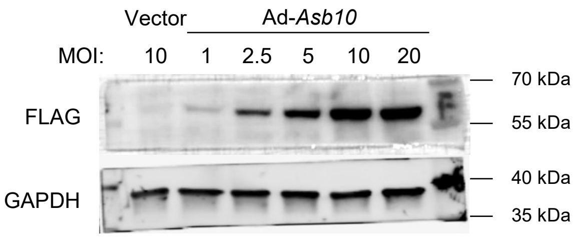

Figure S8A

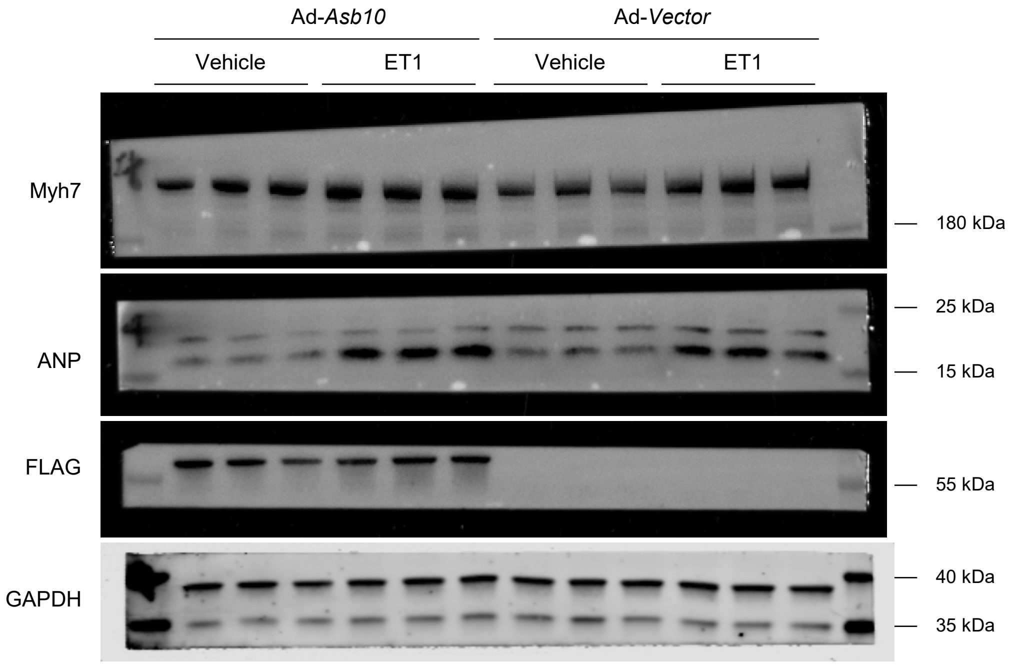

Figure S10B

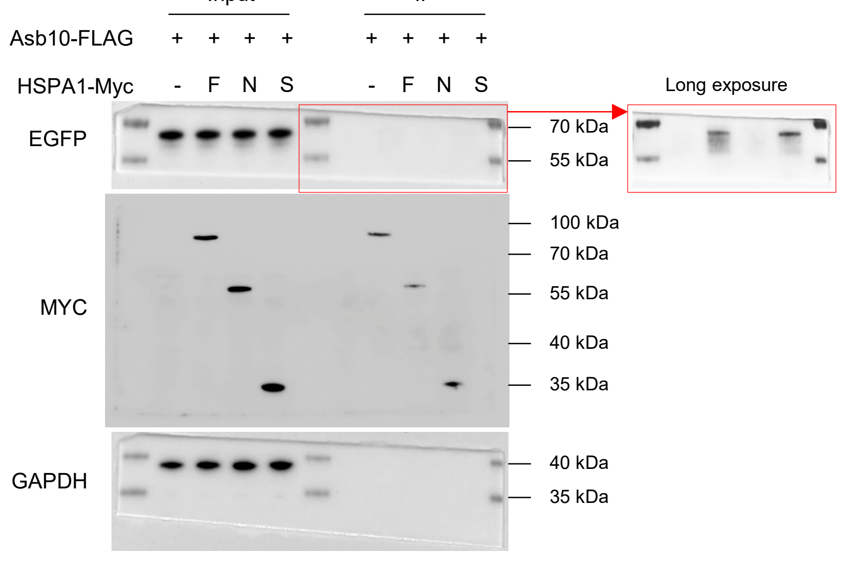

Figure S11A

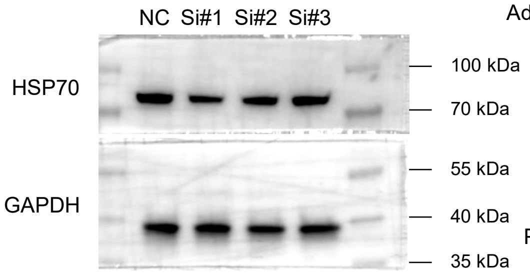

Figure S11B

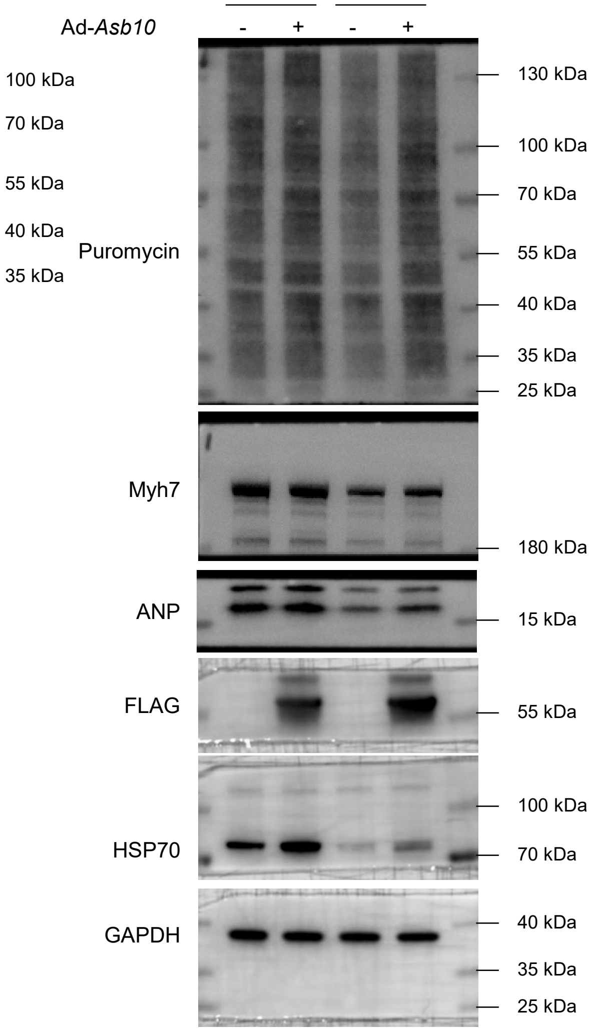

Figure S11C

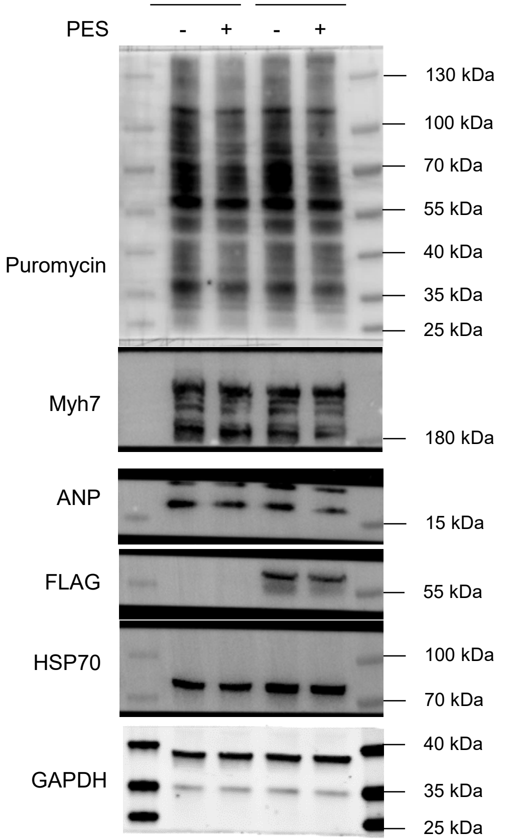

Figure S12A

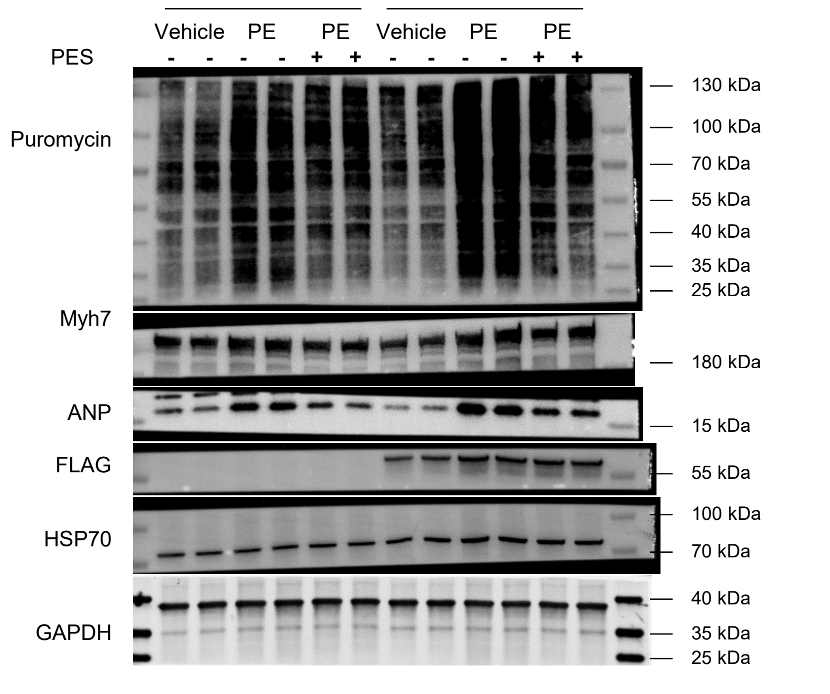

Figure S13B

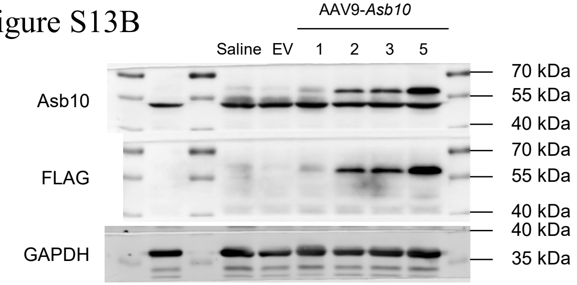

Figure S13C

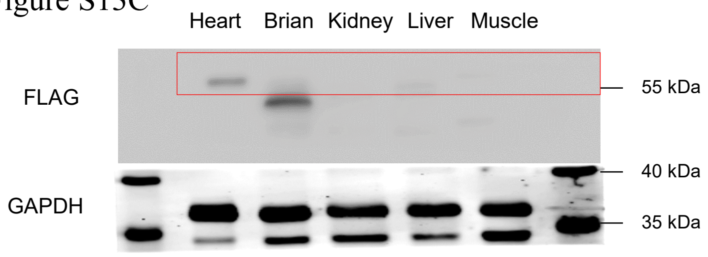

Figure S16A

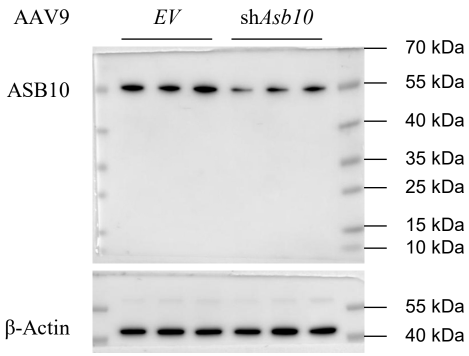

Figure S16B

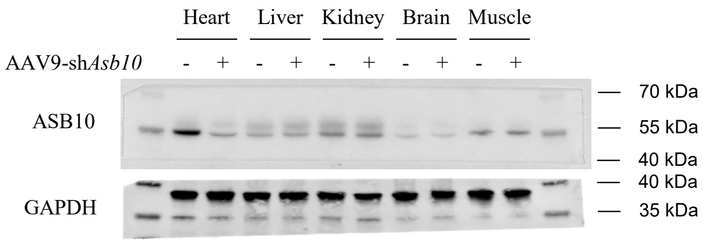

Supplement: Supplementary file 3 — Original Gel Data [file 41419_2025_7735_MOESM3_ESM.pdf]
